# HIV cascade, key indicators, and other epidemiological metrics in Australia (2004–2023): a retrospective analysis

**DOI:** 10.1016/j.lanwpc.2026.101905

**Published:** 2026-06-16

**Authors:** Richard T. Gray, Hamish McManus, Jonathan M. King, Gladymar Pérez Chacón, John S. Rule, Kathy Petoumenos, Andrew E. Grulich, Rebecca J. Guy, Skye McGregor

**Affiliations:** a Kirby Institute, UNSW Sydney, NSW, 2052, Australia; b National Association for People with HIV Australia (NAPWHA), Newtown, NSW, 2042, Australia

**Keywords:** HIV cascade, HIV epidemic trends, High-income country, 95-95-95, Gaps in HIV, Diagnosis and care

## Abstract

**Background:**

We aimed to describe Australia's overall progress towards ending AIDS as a public health threat between 2004 and 2023 using a retrospective analysis of the HIV cascade, key indicators, and other epidemiological metrics.

**Methods:**

We used mathematical modelling, national HIV notifications, cohort, and administrative data to calculate annual estimates for the HIV cascade of care, the number of annual new HIV infections, and related HIV epidemic metrics between 2004 and 2023, for the overall population and males and females. We used piecewise negative binomial regression to determine changes in trajectory and annual rate ratios (ARRs) for each cascade step and metric.

**Findings:**

We estimated there were 30,010 (range: 26,700–35,220) people living with HIV in Australia at the end of 2023 with 25,650 males (85.5%) and 4080 females (13.6%). The percentage diagnosed increased slowly over time and remained below the first UNAIDS 95 target (93.0% of 30,010 people living with HIV in 2023). The percentage diagnosed and on treatment crossed the second UNAIDS 95 target in 2023, reaching 96.6% of 27,650 people diagnosed with HIV, following a large fall in the number untreated between 2010 and 2015. The percentage of people treated with a suppressed viral load first achieved the third UNAIDS 95 target in 2017 (and was 97.5% of 26,700 people on ART in 2023). Changes in the cascade gaps coincided with an increase, stabilisation, and then fall in HIV notifications and estimated new HIV infections in Australia. Most metrics showed substantial improvement over the 2004–2023 period, including during the COVID-19 era. However, there was a large increase in notifications in 2023 following the easing of COVID-19 restrictions, and the number of people living with undiagnosed HIV has remained steady. The metrics for males had very similar trends to the overall population whereas among females the HIV epidemic showed a continuous growth since 2004. Females had a smaller diagnosis gap than males (94.3% vs 91.6%) but a larger treatment gap (94.1% vs 98.2%).

**Interpretation:**

While Australia saw substantial progress in the HIV cascade metrics since 2004, it is not on track to reach all 95-95-95 targets by 2025. Despite large falls in new infections, there are persistent gaps in the cascade related to diagnosis. Further efforts and investments are needed to address barriers to care and ensure the end of HIV as a public health threat in Australia.

**Funding:**

Australian Centre for Disease Control and the Australian 10.13039/501100000925National Health and Medical Research Council (NHMRC).

**Research in context**

**Evidence before this study**

The HIV cascade has become a central tool for monitoring progress towards national and international HIV targets and is a key component of most countries' HIV surveillance systems. Cascade estimates along with prevalence and incidence are part of UNAIDS annual reporting of country level HIV epidemics. HIV cascade estimates predominantly focus on population level cross-sectional snapshots using a combination of data sources for a single year. ‘Longitudinal’ HIV cascades from cohort data describing the flow and transitions through the cascade have become more common. There is an extensive literature on HIV cascades. Using the search string “(HIV OR AIDS) AND (continuum OR cascade OR continua OR spectrum of care) AND (trend OR trends OR longitudinal)” in PubMed without language restrictions or a start date on 22 April 2026 returned 649 publications. HIV cascade estimates are also reported frequently in the grey literature with numerous countries and regions including the cascade in their surveillance and monitoring reports. Several countries have reported the achievement of the 90-90-90 and 95-95-95 UNAIDS targets and progress towards ending AIDS as a public health threat. A number of systematic reviews have provided collated estimates and comparisons of methods and data used to produce cascades. These include reviews of the HIV cascade for key populations such as men who have sex with men in sub-Saharan Africa. Where available, analyses of long-term clinical cohort data have provided trends in HIV cascade estimates at the sub-national level (for example in British Columbia, Canada). Similarly, trends for individual metrics have been published for the national and sub-national level and for key populations using estimates from available data or modelling. Despite the substantial literature and reporting of estimates for the HIV cascade and other epidemiological measures, gaps remain and there is inconsistency in reporting with often only a subset of metrics analysed. Long term trend analyses showing the potential impact of changes in public policy on the cascade gaps and other metrics are rarely presented. The detailed HIV notification and clinical cohort data available in Australia has enabled us to estimate national trends in the HIV cascade, annual new infections, and other epidemiological metrics over a 20-year period to the end of 2023. We believe this is one of the first studies to evaluate the timeseries of a suite of epidemiological metrics, including each HIV cascade step and gap, over a long period and assess trends in progress towards HIV elimination targets.

**Added value of this study**

We provide time series estimates and trend analysis of 19 metrics including each HIV cascade step and gap for the overall population as well as for males and females. While some metrics are related or associated, they provide a comprehensive view of Australia's overall HIV epidemic since the mid-2000s. This period extends from a time when treatment initiation was delayed until CD4 count was <500 cells/mm³, through the period that treatment as prevention and pre-exposure prophylaxis became a focus, and into the early years of the COVID-19 pandemic. Our results show rapid changes in the metrics related to treatment and viral suppression with a large decline in new infections in recent years. These changes generally occured after changes in public health policy and treatment guidelines and suggest there has been substantial progress towards HIV elimination targets. However, some metrics point towards persistent gaps in the response which may hinder further progress.

**Implications of all the available evidence**

Australia is a high-income country with the resources available to achieve the UNAIDS 2030 targets, yet the persistence of gaps related to diagnosis highlight the level of effort required to end the HIV/AIDS epidemic and end AIDS by 2030. Continued efforts are required to ensure progress is sustained and accelerated, close any gaps in the HIV cascade among key populations, and ensure there is no resurgence in new infections. Further analysis of trends in the HIV cascade, new infections and other epidemiological metrics at the national and sub-national level and for each key population could enable a tailored response to end AIDS as a public health threat.

## Introduction

With the widespread availability of highly effective HIV treatment and prevention strategies, aspirational international goals are now focused on ending AIDS as a public health threat by 2030.^1^ UNAIDS has set targets for the diagnosis, care, and treatment of people living with HIV to promote progress towards this aim. Initial targets were to diagnose 90% of all people living with HIV, have 90% of those diagnosed treated with anti-retroviral therapy (ART), and have 90% of those treated achieving viral suppression by 2020.^2^ These “90-90-90” targets were updated in December 2020 to a corresponding set of interim “95-95-95” targets for 2025.^1^ Modelling has estimated that achieving all three 95 targets globally would reduce HIV incidence by almost 90% between 2010 and 2030.^3^

The HIV diagnosis and treatment cascade (HIV cascade) has become the standard reporting tool for tracking progress towards the UNAIDS targets. It consists of estimates for up to five steps in the HIV diagnosis, care and treatment pathway and provides a framework for service providers and policymakers to measure progress in the delivery of HIV testing, care, and treatment.^1^^,^^4^ These steps include the overall number of people living with HIV, the number with HIV previously diagnosed, the number diagnosed and retained in care, the number on ART, and the number achieving viral suppression. Alongside the key epidemiological metrics of new HIV infections and HIV-related deaths, estimates for the HIV cascade steps provide insights into the characteristics of local HIV epidemics.^3^^,^^5^

Numerous studies have published cascade estimates and trends in specific epidemiological metrics at the national and sub-national level and for specific sub-populations.^4^^,^^6^^,^^7^ We are not aware of any studies reporting a comprehensive national analysis of HIV epidemiology using a wide range of metrics (including estimates for all steps of the HIV cascade) to observe long-term trends and potential associations with changes in public health policy. Using surveillance data and other administrative datasets, such an analysis would provide a comprehensive overview of progress towards ending AIDS as a public health threat and the feasibility of achievement. This analysis could also provide insights into the overall change in HIV epidemiology associated with updates to clinical guidelines and the scale-up of interventions such as the early initiation and scale-up of ART for treatment as prevention (TasP),^8^, ^9^, ^10^ the roll-out of oral pre-exposure prophylaxis (PrEP),^11^^,^^12^ and whether interruptions to health services due to the COVID-19 response introduced further obstacles towards achieving HIV targets.^13^^,^^14^ Though the causality of any changes and when they occur would require further investigation.

Australia is a high-income country that provides unrestricted access to ART to all people living with HIV, although out-of-pocket co-payment costs for treatment and monitoring remain in some jurisdictions. Australia has committed to achieving the UNAIDS 95-95-95 targets and to virtually end HIV transmission by 2030, defined as a 90% reduction in new infections compared to a 2010 baseline.^15^ The aim of our study was to conduct an epidemiological retrospective analysis of Australia's HIV epidemic and to assess how it has changed over time. To do this we estimated each step of the HIV cascade, the “gaps” in the cascade, annual new HIV infections, and other epidemiological metrics over the 20-year period from 2004 to 2023 using data from Australia's multi-tiered HIV surveillance system.^16^ We then conducted a trend analysis to determine when changes in trend occurred and the annual rate of change. The analysis was completed for the population overall and for males and females.

Our results show the long-term overall impact of Australia's HIV response during the implementation of biomedical focused prevention strategies such as TasP since 2011, and the roll-out and scale-up of oral PrEP since 2016 primarily among gay, bisexual and men who have sex with men. They also show the impact of the COVID-19 pandemic, when access to health services declined and substantial changes to migration occured following the closure of Australia's national and several interstate borders during 2020–21.

## Methods

Since 2014, estimates for Australia's HIV cascade have been published annually in the HIV, viral hepatitis and sexually transmissible infections in Australia: Annual surveillance report.^16^ The definitions, methods and data sources used in these reports are reviewed and updated annually following consultation with a national stakeholder reference group consisting of representatives of federal and state and territory health departments, peak community organisations, and other researchers. For this study we applied and expanded the methods to produce best estimates and uncertainty ranges for each step of the HIV cascade and the other metrics for each calendar year between 2004 and 2023, as summarised in the following sections. We also describe the methods used to analyse trends. This study followed the STROBE reporting guidelines for observational studies (checklist provided in Supplementary Appendix 6).

The number of people living with HIV who are retained in care (which we define as either on ART or have had one viral load or CD4 count test during the calendar year) is presented for 2023 alone, as robust retention data are only available since 2013.^17^ The analyses were applied to the overall population and males and females separately using the same method and population specific data as described below. We did not produce estimates for trans and gender diverse people because the low number of HIV notifications preclude production of robust estimates (98 HIV notifications among this population during 2004–2023; 6 with unknown or missing gender). Since 2019, gender for national HIV notifications data collection has been a two-step process with sex at birth and gender both collected and used to generate the derived ‘gender’ variable. Further details of the methods and data sources are provided in the Supplementary Appendix and in the latest Australian HIV surveillance report.^16^

We conducted all coding and analyses using R version 4.3.2 with open access code available online.^18^^,^^19^ This code is part of a larger ongoing project on Australian diagnosis and care cascades for blood borne viruses and bacterial sexually transmitted infections and is constantly updated. The specific scripts and release associated with the results in this study are provided in the Supplementary Appendix (p 1 and Section 5, p 93).

### Estimates for number of people living with diagnosed HIV

Annual estimates of the number of people living with HIV who have been diagnosed were obtained by subtracting the number of deaths (estimated using population mortality rates from a previous linkage study and clinical cohort data) and the number of emigrants (people living with HIV who have left Australia) from the cumulative number of unique HIV notifications (using an established statistical method described in the Supplementary Appendix 1.1 p 2).^20^ The only data available for emigration are overseas migration rates for the general population and overall 6-month follow-up data of people notified with HIV in the state of New South Wales (NSW). Given these limited data, we applied a multiplier to the general population emigration rate and calibrated it so that the proportion of people diagnosed and on ART matched the estimate from an ongoing national HIV data linkage study between 2015 and 2020 (provided by authors H. McManus and G. Pérez Chacón).^21^ This multiplier was calibrated separately for the overall population, males, and females (see Supplementary Appendix 1.6 p 8–10). We considered people living with HIV in Australia who have been previously diagnosed overseas to be part of the diagnosed population even if they are not in care locally.

### Estimates for the number of people living with HIV and the number undiagnosed

To estimate the overall number of people living with HIV, both diagnosed and undiagnosed, we used the European Centre for Disease Prevention and Control (ECDC) HIV Modelling Tool (version 1.3.0) to estimate the proportion undiagnosed each year.^22^^,^^23^ We ran 100 simulations (excluding those previously diagnosed overseas and those with evidence of seroconversion), the minimum number recommended when using the tool, to obtain a best estimate and bootstrapped 95% confidence interval (CI; see Supplementary Appendix 1.2 p 5 and 6). We describe the CI as the range for the proportion undiagnosed, to be consistent with uncertainties for the other metrics. Additional simulations did not change the best estimates and range for the proportion undiagnosed. The number of males, females, and people overall living with HIV were then estimated by dividing the corresponding number with diagnosed HIV by one minus the population proportion undiagnosed.

### Number treated and the number with a suppressed viral load

People living with HIV who have received ART at least once during the calendar year are classified as ’on treatment’. This is different to the UNAIDS Indicator Registry definition which uses ‘on ART’ at the end of the year.^24^ We used this alternative definition because people living with HIV in Australia can get multiple scripts during a single clinician visit, which provides treatment for potentially up to 12 months. This means such a person would still be considered on treatment at the end of the calendar year. Viral suppression is defined as a viral load of <200 HIV-1 RNA copies/mL at last test during the calendar year. We used this threshold rather than the 1000 copies/mL threshold used by UNAIDS,^24^ because it was considered by the Australian HIV Diagnosis and Care Cascade National Reference Group to be the most clinically relevant for the care of people living with HIV, and it is the threshold used in Australia's National HIV Strategy.^15^ Using our definition will result in a conservative estimate for the percentage of people on ART with a suppressed viral load.

Between 2004 and 2013, estimates for the number treated were obtained from the Australian HIV Observational Database (AHOD) cohort. After 2014, a full population data set from Australia's Pharmaceutical Benefits Scheme (PBS),^25^ which records all script claims processed by the country's publicly funded universal health care system called Medicare, became available. To the PBS data we added an estimate for the number of temporary residents with HIV who were taking ART. Temporary residents are people who live in Australia but are ineligible for subsidised medicines through Medicare which means their ART use is not recorded in the PBS data. In the past, temporary residents could access ART through pharmaceutical company compassionate access schemes.^26^ They can now access subsidised ART through an Australian Federal Department of Health, Disability and Ageing program established in 2022. Since data from this program is not available by gender, we split the overall number of temporary residents with HIV on ART into males and females using the proportions from the Australian HIV Observational Database Temporary Residents Access Study (ATRAS).^26^

We merged the AHOD and PBS based estimates through curve fitting (see Supplementary Appendix 1.4 p 7 and 8). We applied the proportion on treatment with a suppressed viral load in AHOD to the number treated to estimate the number with a suppressed viral load, using the 95% CI as the range.

### Estimates of new HIV infections and other epidemiological metrics

The ECDC model also provided estimates for the number of new HIV infections each year for each population, the key indicator for ending AIDS as a public health threat. From the cascade estimates we calculated the number of people in the “gaps” (the number undiagnosed, diagnosed but untreated, and treated but with an unsuppressed viral load). For simplicity, we did not estimate the uncertainty in these gaps (which could be substantial) due to their interdependence and because it was not used in the trend analysis.

While the key metrics for understanding HIV epidemics are new HIV infections or incidence, HIV prevalence, the number of HIV related deaths, and the steps of the HIV cascade, other epidemiological metrics can provide additional insights and be useful where data are limited. Using the estimates for the cascade steps and new infections, we also produced estimates for following the epidemiological metrics with further details in Table 1: the Yearly diagnosed fraction (YDF)^29^^,^^30^; the Case detection rate (CDR)^30^; the Incidence prevalence ratio (IPR)^5^^,^^27^; and the Incidence mortality ratio (IMR).^5^^,^^28^

**Table 1:** Definition and interpretation of the other epidemiological metrics.

Metric	Definition	Formula	Interpretation
Yearly diagnosed fraction (YDF)	The number of notifications (excluding cases previously diagnosed overseas) during the year divided by the sum of the number of notifications during the year and the total number undiagnosed at the end of the year.	$\frac{Number\;of\;new\;notifications}{\left(\begin{array}{c} Number\;of\;new\;diagnoses\;+\\ Number\;undiagnosed \end{array}\right)}$	The inverse of the YDF gives the number of years required to diagnose everyone with undiagnosed HIV if there are no new infections and a constant number of people are diagnosed each year. For example, if the YDF = 0.2 and there are 1000 undiagnosed people, then 200 people (1000 ∗ YDF) would need to be diagnosed each year for five (1/YDF) years. Note that the YDF will increase to 100% in the final year in this example.
Case detection rate (CDR)	The ratio of new notifications (excluding cases previously diagnosed overseas) to new infections each year.	$\frac{Number\;of\;new\;notifications}{Number\;of\;new\;infections}$	This metric indicates whether the number of people with undiagnosed HIV is increasing (CDR <1) or declining (CDR >1). Ideally, the CDR should be greater than one and as high as possible to reduce the population living with undiagnosed HIV as quickly as possible.
Incidence prevalence ratio (IPR)	The number of new infections during each year divided by the number living with HIV at the start of each year (reported as a percentage).	$\frac{100\%\;\times \;Number\;of\;new\;infections}{Number\;of\;people\;living\;with\;HIV}$	A declining IPR suggests prevention efforts are contributing to a decline in transmission. Given an estimate for the lifespan of an individual post-HIV acquisition, an IPR benchmark can be estimated below which a self-sustained epidemic cannot occur (and incidence will primarily be through increasing prevalence via the immigration of people already living with HIV). The UNAIDS has set a global target for the IPR of 0.03 based on a global average survival time of 33 years for people living with HIV. For Australia this threshold is lower due to a longer life expectancy and is estimated to be 0.022.^27^
Incidence mortality ratio (IMR)	The number of new infections during each year divided by the estimated number of all cause deaths among people living with HIV during the year.	$\frac{Number\;of\;new\;infections}{\begin{array}{c} Number\;of\;all\;cause\;deaths\;among\;\\ people\;living\;with\;HIV \end{array}}$	Like the IPR, this metric provides an indication of the direction of an epidemic. In a population with high levels of ART coverage and viral suppression, an IMR <1 would seem to suggest a reduction in the number of people living with HIV. However, this reduction could be counteracted by the immigration of people living with HIV. A declining IMR suggests a decline in the contribution of transmission to the number of people living with HIV.^28^

These additional metrics are closely aligned and associated with the estimates for the HIV cascade and new infections, but they can provide an alternative view of the epidemic and emphasise changes and gaps. For a country like Australia where there is a growing number of notifications among people previously diagnosed overseas the trends in the IPR and IMR are more important than the magnitude.

### Statistical analysis

We assessed annual trends for each HIV cascade step, gap, and metric (including notifications with and without people previously diagnosed overseas) for each population by fitting continuous piecewise negative binomial regression models to the best estimates of each metric (excluding the uncertainty ranges for simplicity and interpretability), with year as an independent covariate. For metrics which are a proportion (or percentage) we used the denominator as an offset to ensure the fitted estimate remained below one. Regression models with multiple change points for each metric were produced by selecting the model with the minimum Bayesian information criterion (BIC) after excluding those with change points within four years of each other or 2004 (see Supplementary Appendix 3 p 22). We allowed change points within four years of 2023 given the potential impact of the COVID-19 pandemic. For the selected model, we report estimates with 95% CIs for the time of each change point and the annual rate ratio (ARR) for each segment between change points. The ARR shows the annual increase (value > 1) or decrease (value < 1) of the value of a metric (if the metric is constant then the ARR = 1). It has a multiplicative effect over time so even a small increase or decrease (i.e. a value close to one) can eventually result in a large change.

### Ethics statement

No formal ethical approval was required for this analysis as we used modelling estimates generated through routine surveillance mechanisms. Data custodian and ethical approval details for the national HIV linkage study data are provided in the Supplementary Appendix 1.6, page 9.

### Role of funding source

Sources of external funding did not influence the study design or data collection, analysis, interpretation or writing of the report. The corresponding author had full access to all the data in the study and had final responsibility for the decision to submit for publication.

## Results

The best overall estimates and uncertainty ranges for each cascade step, cascade gap, and each epidemiological metric along with all change points and ARRs between change points obtained by the trend analysis are provided in Table 2 and summarised below. Corresponding tables and figures for males and females are provided in the Supplementary Appendix (Sections 2.2 and 3.1 and Sections 2.3 and 3.2, respectively). The reported percentages for the HIV cascade steps are derived using the corresponding best estimates.

**Table 2:** Summary results for each cascade HIV step and metric for the overall population.

Metric	2004 best estimate (range)	2023 best estimate (range)	Change point estimates (95% CI)	ARR between change points (95% CI)
**HIV cascade steps**				
Number of people living with HIV	15,580 (14,410–16,840)	30,010 (26,700–35,220)	2014.7 (2014.4–2014.9)	1.042 (1.042–1.042)
			2019.5 (2019.3–2019.6)	1.033 (1.032–1.033)
				1.016 (1.015–1.016)
Diagnosed	12,930 (11,890–14,040)	27,650 (26,700–32,390)	2013.6 (2013.3–2013.8)	1.05 (1.05–1.05)
			2018.6 (2018.5–2018.8)	1.039 (1.039–1.04)
				1.021 (1.021–1.022)
Receiving ART	7600 (6000–9300)	26,700 (26,700–27,070)	2009 (2008.4–2009.6)	1.074 (1.072–1.077)
			2015.4 (2015.2–2015.6)	1.097 (1.095–1.1)
				1.036 (1.035–1.037)
Virally suppressed	4570 (3460–5810)	26,040 (25,780–26,650)	2014.6 (2014.4–2014.8)	1.131 (1.129–1.133)
				1.043 (1.041–1.045)
**HIV cascade gaps**				
Number with undiagnosed HIV	2650	2360	2019.6 (2018.5–2020.7)	0.997 (0.996–0.998)
			2021.7 (2021.3–2022)	1.029 (1‒1.059)
				0.888 (0.862–0.915)
Number diagnosed with HIV but untreated	5330	950	2009.5 (2009.3–2009.7)	1.016 (1.011–1.021)
			2016.3 (2015.9–2016.7)	0.874 (0.871–0.878)
			2021.7 (2021.6–2021.9)	0.94 (0.933–0.947)
				0.666 (0.642–0.691)
Number on ART but with an unsuppressed viral load	3030	670	NA	0.908 (0.898–0.918)
Percentage of people living with HIV diagnosed	84.6	93.0	2008.4 (2007.7–2009.1)	1.001 (1‒1.002)
				1.006 (1.006–1.007)
Percentage diagnosed with HIV on ART	58.8%	96.6%	2009 (2008.5–2009.5)	1.021 (1.018–1.025)
			2014.5 (2014.3–2014.8)	1.05 (1.048–1.052)
				1.01 (1.009–1.011)
Percentage on ART virally suppressed	60.1%	97.5%	2010.3 (2009.9–2010.6)	1.058 (1.054–1.061)
				1.009 (1.008–1.01)
Percentage of people living with HIV virally suppressed overall	29.3%	86.8%	2014.4 (2014.1–2014.6)	1.086 (1.084–1.088)
				1.017 (1.015–1.019)
**HIV cascade related metrics**				
Notifications excluding previously diagnosed overseas	894	722	2009.4 (2003.1–2015.7)	1.006 (0.994–1.017)
			2016.7 (2016.3–2017.1)	1.015 (1.006–1.024)
			2021.6 (2021.3–2021.8)	0.859 (0.846–0.873)
				1.259 (1.168–1.356)
Notifications including previously diagnosed overseas	918	1302	2014 (2013.7–2014.3)	1.034 (1.032–1.036)
			2019.2 (2019–2019.3)	0.983 (0.98–0.987)
			2021.3 (2021.2–2021.3)	0.77 (0.751–0.79)
				1.453 (1.421–1.485)
Notifications of HIV previously diagnosed overseas	24	580	2008.2 (2007.6–2008.9)	1.604 (1.466–1.755)
				1.078 (1.063–1.093)
Annual new infections (range = 95% CI)	910 (890–930)	380 (230–540)	2012.5 (2012.3–2012.7)	1.01 (1.009–1.012)
			2019.5 (2019.4–2019.6)	0.951 (0.95–0.953)
				0.841 (0.836–0.846)
Yearly diagnosed fraction (YDF)	0.25 (0.24–0.26)	0.25 (0.22–0.29)	2017.5 (2017.2–2017.9)	1.017 (1.014–1.02)
			2021.7 (2021.4–2021.9)	0.86 (0.841–0.879)
				1.371 (1.275–1.474)
Case detection rate (CDR)	0.95 (0.93–0.98)	2.04 (1.46–3.38)	2019 (2017.5–2020.5)	1.026 (1.02–1.031)
			2021.4 (2021.1–2021.8)	0.871 (0.757–1.001)
				1.665 (1.449–1.913)
Incidence prevalence ratio (IPR; %)	6.1% (5.6‒6.7%)	1.3% (0.7–2.1%)	2012.4 (2012.3–2012.6)	0.968 (0.967–0.969)
			2019.5 (2019.3–2019.6)	0.918 (0.916–0.919)
				0.827 (0.822–0.831)
Incidence mortality ratio (IMR)	7.93 (5.27–12.1)	1.28 (0.54–2.63)	2015 (2013.4–2016.6)	1.004 (0.981–1.028)
				0.835 (0.8–0.872)

Acronyms: ARR, Annual Rate Ratio; CI, Confidence Interval.

The ARR shows the annual increase (value > 1) or decrease (value < 1) of the value of a metric (if the metric is constant then the ARR = 1). Note results that are generated using statistical methods have uncertainties specified by the resulting 95% confidence interval. This includes the annual number of new infections which were obtained using bootstrapping within the ECDC HIV Modelling Tool. Uncertainties for each metric are called ranges because they are generated using a combination of data and calculations. Cascade step, cascade gap and new HIV infection estimates rounded to nearest 10. (Tables S11 and S12 in the Supplementary Appendix provide the results for males and females).

### HIV epidemiology in Australia at the end of 2023

**Fig. 1:**
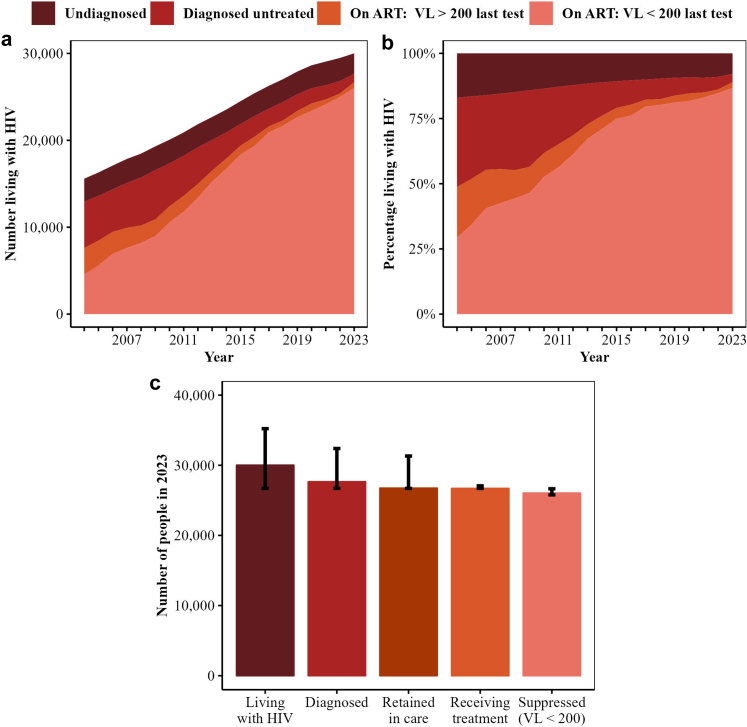
**Australian HIV cascade to the end of 2023 for the overall population**. a) Estimated numbers and b) percentages of people living with HIV in each step of the HIV cascade over 2004–2023. c) HIV cascade best estimates and uncertainty ranges at the end of 2023 including retained in care (note lower range of Diagnosed and Retained in care adjusted to equal lower range of number Receiving treatment). The annual estimates and uncertainty ranges for each step of the overall cascade are in Table 2. HIV cascade estimates for males and females are provided in the Supplementary Appendix (Tables S11 and 12).

During 2023, Australia recorded 722 (620 male and 95 female) notifications with a further 580 (466 male and 111 female) notifications among people previously diagnosed overseas (44.5% of the total 1302 notifications). A summary of Australia's overall HIV epidemiology to the end of 2023 is shown in Fig. 1. By the end of 2023, there were an estimated 30,010 (range: 26,700–35,220) people living with HIV in Australia, of whom 27,650 (range: 26,700–32,390) or 92.1% had been diagnosed (equating to 7.9%, n = 2,360, being undiagnosed). Of people diagnosed, 96.7% (n = 26,740) were retained in care and 96.6% (n = 26,700) were receiving treatment. Of people treated 26,040 (range: 25,780–26,650) or 97.5% had a suppressed viral load (86.8% of all 30,010 people living with HIV).

### Overall trends in HIV cascade steps and gaps

**Fig. 2:**
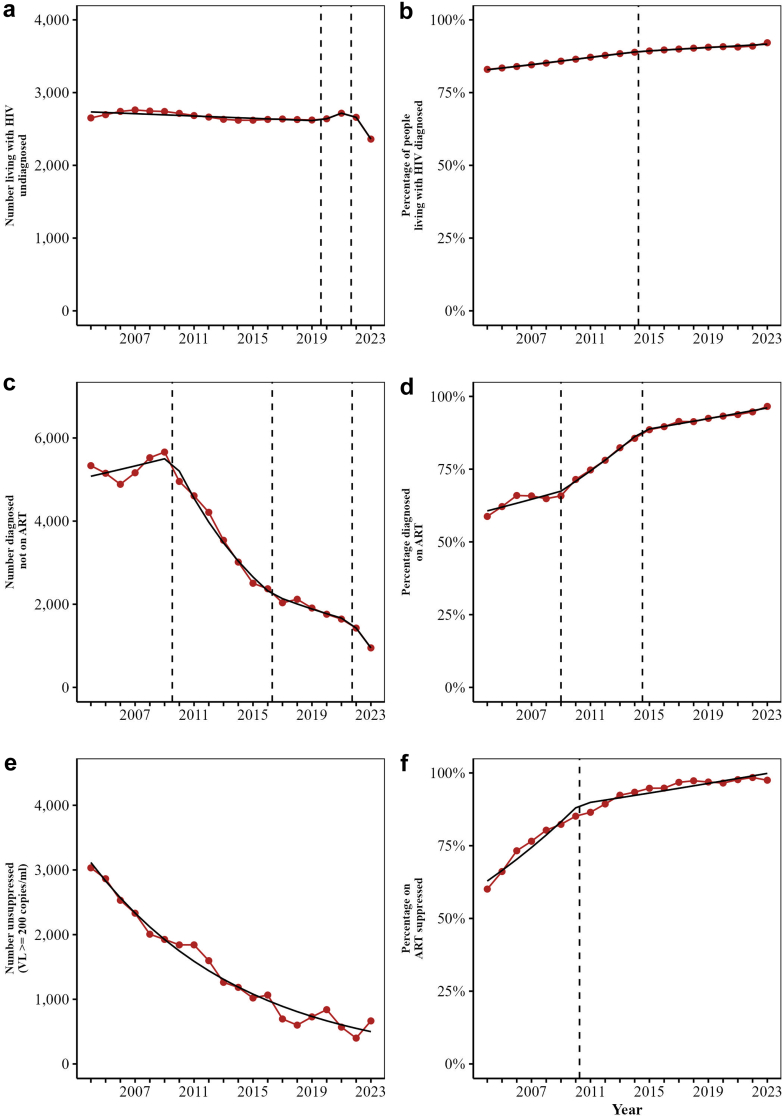
**Estimates and trends for the number(a, c, e)and percentage(b, d, f)of people living with HIV in the gaps of the HIV cascade between 2004 and 2023 for the overall population.** Red lines show the best estimated values from the HIV cascade calculations. Black lines show the best fitting model predictions. Vertical dashed lines show the location of detected change points. No uncertainty ranges were produced for these metrics due to them being calculated from the cascade steps which are interdependent. The estimates and analysis results for the overall population are in Table 2. Corresponding estimates for males and females are provided in the Supplementary Appendix (Tables S11 and 12).

**Fig. 3:**
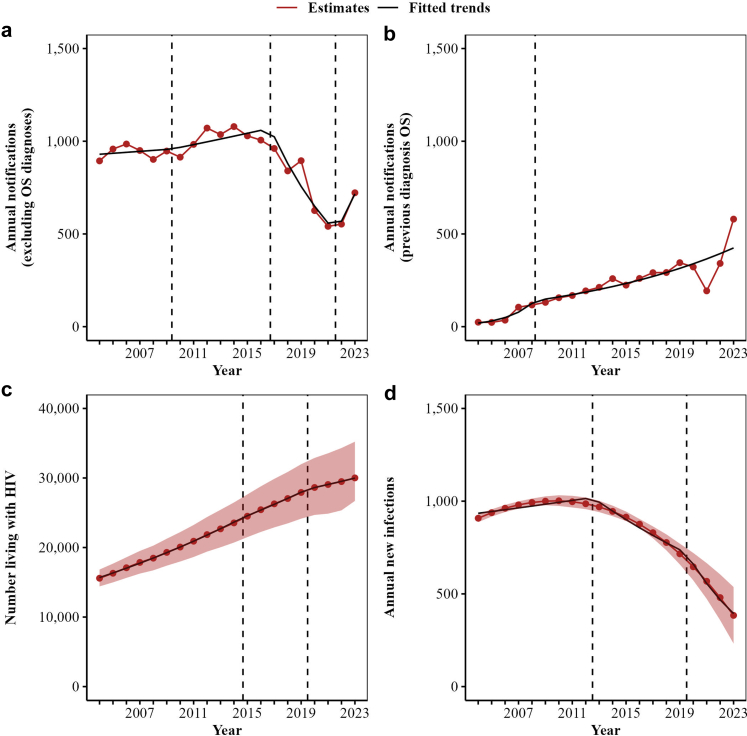
**Trends in notifications(aand b), number of people living with HIV(c),and annual new infections(d)between 2004 and 2023 for the overall population**. Red lines and shading show the best estimated values and ranges from the HIV cascade calculations. Note there is no red shading for annual notifications data as they are directly from the national HIV registry. Black lines show the best fitting piecewise negative binomial model predictions. Vertical dashed lines show the location of detected change points. (OS = overseas). The estimates and analysis results for the overall population are in Table 2. Corresponding estimates for males and females are provided in the Supplementary Appendix (Tables S11 and 12).

The changes in the HIV cascade estimates and gaps are shown in Figs. 1 and 2. Between 2004 and 2023, there were substantial increases in the estimated number and proportion of people diagnosed (n = 12,930, 83.0% to n = 27.60, 92.1%; Fig. 2b), on ART (n = 7600, 58.8% to n = 26,700, 96.6%; Fig. 2d), and with suppressed viral loads (n = 4570, 60.1% to n = 26,040, 97.5%; Fig. 2f). The annual growth rate of people living with HIV decreased after 2015 and then decreased further after 2020 (Figs. 1a and 3c). Similarly, there were falls in the growth rate for people diagnosed (Fig. 1a). Over the whole 2004–2023 period the number treated and virally suppressed increased at a faster rate than the first two steps of the cascade (Table 2). The number of people living with HIV receiving ART each year grew fastest between 2004 and 2009, while the growth in the number with a suppressed viral load slowed after 2009.

As shown in Fig. 2a, the number undiagnosed declined gradually overall from 2004 until 2019, quickly increased and then declined during the COVID-19 period to reach a minimum of 2360 people in 2023. The overall percentage diagnosed remained well above 75% over 2004–2023 with a slow but almost continual increase over the period, crossing the original UNAIDS 90% target in 2018 (Fig. 2b). The number of diagnosed people not on treatment declined rapidly after 2009, from a relatively stable level just below 6000 before a slower decline between 2017 and 2021 and then a more rapid decline to the end of 2023 (noting that the final change point may be spurious given its proximity to 2023; Fig. 2c). The percentage diagnosed on treatment has increased substantially from 58.8% in 2004 to reach 90% in 2017 and 95% in 2023. There was a rapid increase between 2009 and 2014 but only a slow increase since 2014 (Fig. 2d). Finally, the number with an unsuppressed viral load has declined continually (Fig. 2e). The percentage on ART with a suppressed viral load was 60.1% in 2004 but increased rapidly reaching 90% in 2013 and 95% in 2017 (Fig. 2f).

### Overall trends in key epidemiological metrics

The changes in HIV notifications, the number living with HIV and annual new infections are shown in Fig. 3. Annual notifications had different trends depending on whether people previously diagnosed overseas were included or excluded. When including those previously diagnosed overseas, annual notifications steadily increased between 2004 and 2014, before declining slowly between 2014 and 2019 and then rapidly declining and increasing during the COVID-19 period (Table 2). This pattern reflects the closure and re-opening of the national borders of Australia by the national government in response to the COVID-19 pandemic. In contrast, there was a slower and more continual increase in annual notifications excluding those previously diagnosed overseas until 2016 (Fig. 3a). There was then a steady decline until 2021 before a recent increase. Considering annual notifications among people previously diagnosed overseas separately, there has been an almost continual increase in the number since 2008 with only a rapid fall and increase in the number around 2021 (which was too fast for a statistically significant change point to be detected using our methodology; Fig. 3b).

Estimated new infections generally had different trends to notifications (both including and excluding those previously diagnosed overseas). There was an earlier peak of 1002 new infections in 2010 (point estimate; Fig. 3d) with a gradual increase between 2004 and 2012 before a decline between 2013 and 2019 and then a faster decline during the COVID-19 period. This corresponds to a 61.7% decline in new infections since 2010 to 384 (range: 232–537) new infections in 2023.

### Overall trends in other epidemiological metrics

**Fig. 4:**
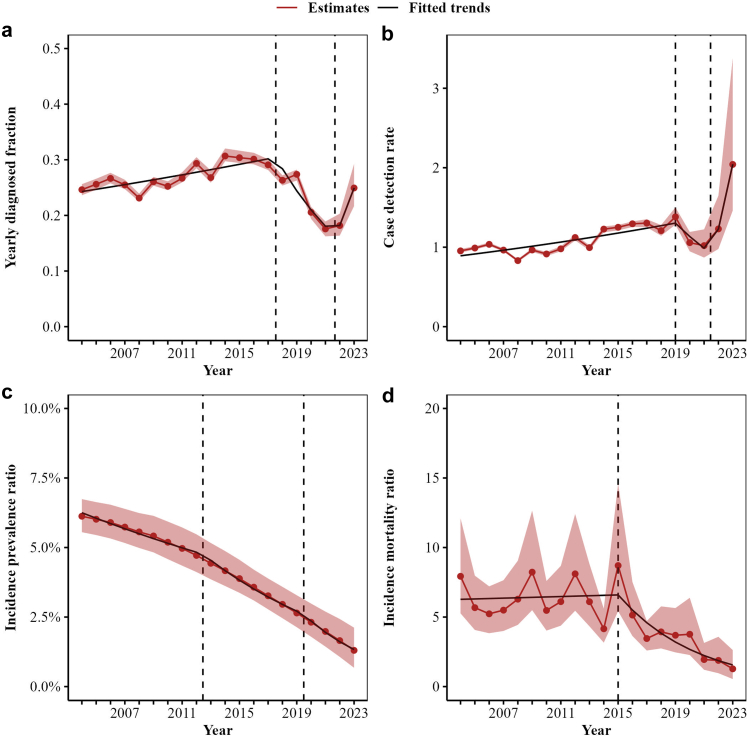
**Trends in other cascade related metrics between 2004 and 2023 for the overall population.** a) YDF, b) CDR, c) IPR and d) IMR. Red lines and shading show the best estimated values and ranges from the HIV cascade calculations. Black lines show the best fitting piecewise negative binomial model predictions. Vertical dashed lines show the location of detected change points. The estimates and analysis results for the overall population are in Table 2. Corresponding estimates for males and females are provided in the Supplementary Appendix (Tables S11 and 12).

The changes in the YDF and CDR since 2004 reflect the changes seen in diagnoses, new infections, and the number undiagnosed but provide more nuance and show contrasting trends (Fig. 4a and b). The YDF initially increased slowly then declined after 2018 before a sharp up tick prior to 2023. The CDR initially had a value close to 1 from 2004 to 2014 with a slightly increasing trend resulting in a value > 1 between 2014 and 2019. During the COVID-19 period the CDR decreased (but remained >1) before increasing rapidly to a value of 2.041 at the end of 2023. This reflects the rapid decline in new infections compared to diagnoses (excluding those previously diagnosed overseas) over this period. The changes in YDF and CDR show the change in new diagnoses in relation to the number undiagnosed between 2004 and 2023 not seen in the first step of the HIV cascade (Fig. 2b). The IPR has decreased continuously since 2004 to 1.3% (range: 0.7–2.1%) in 2023. The IMR fluctuated between 2004 and 2015 with a slightly increasing trend before declining substantially after 2015 to a value of 1.28. This decline in the IMR towards a value of one suggests the number of people living with HIV is stabilising (as seen in Figs. 1a and 3c) and could decline in future if the trend continues, while still taking into consideration migration.

### Trends in the cascade and other epidemiological metrics for males and females

The changes in the estimates for the HIV cascade, cascade gaps, key metrics and other epidemiological metrics for males and females are provided in separate sections of the Supplementary Appendix (Section 2.2 p 14–16, Section 3.1 p 23–29 and Section 2.3 p 18–20, Section 3.2 p 30–35, respectively). By the end of 2023, there were an estimated 25,650 males (85.5% of all 30,010 people living with HIV; range: 23,080–30,950) and 4080 females (13.6% of all 30,010 people living with HIV; range: 3750–4360) living with HIV in Australia. Note the sum of the number of males and females living with HIV does not equal the overall number due to notifications among trans and gender diverse people and because the ECDC modelling tool was run separately for each population.

The HIV cascade for males living with HIV shows 91.6% (n = 23,490) are diagnosed, 98.2% (n = 23,080) diagnosed are on ART, and 97.6% (n = 22,520) on ART have suppressed virus. In comparison, females living with HIV had a higher percentage diagnosed (94.3%, n = 3850) but a lower percentage diagnosed on ART (94.1%, n = 3620), and a slightly lower percentage with a suppressed viral load (96.7%, n = 3510, of those on ART). In terms of trends males had essentially the same trends and change points as for the overall population. This is not surprising given 86% of all notifications between 2004 and 2023 were among males. The metrics for females generally showed continuous trajectories with fewer change points. The number of females living with HIV has shown continuous growth since 2004 but the number with undiagnosed HIV has been declining faster than for males. The number of females diagnosed but not on ART continued to increase until 2015 before declining rapidly (Supplementary Appendix Figure S11c).

## Discussion

Since 2004, Australia has overall made substantial progress towards ending AIDS as a public health threat having achieved all UNAIDS 90-90-90 targets by 2018, surpassing the treatment and suppression 95-95-95 targets in 2023 and 2017, respectively, and achieving large reductions in estimated new infections since 2012. However, according to current trends, Australia is not on track to meet the 95% diagnosed target by 2025 and continuing to meet the other targets is not guaranteed. The number of people living with undiagnosed HIV has only declined slightly and there is a persistent proportion of notifications associated with late diagnosis especially among those born overseas.^16^ This suggests there are persistent gaps in the HIV cascade of care for some population groups which could lead to a resurgence in new infections. Future work will aim to estimate the HIV cascade and other epidemiological metrics for each priority population, including Aboriginal and Torres Strait Islander peoples and overseas born gay and bisexual men. Our cascade estimates and epidemic trends are comparable to those for other high-income countries with concentrated epidemics. For example the United Kingdom, the United States of America, and the Netherlands have all seen falls in diagnoses and new HIV infections overall since the scale-up of TasP and HIV PrEP.^31^^,^^32^ Noting that different definitions need to be taken into consideration when comparing our results with other countries.

The large change in the number diagnosed but untreated between 2011 and 2015 corresponds to changes in clinical guidelines and policy as the benefits of earlier initiation and treatment as prevention became apparent.^9^^,^^33^ The declining trends in notifications, new infections, and the incidence prevalence and incidence mortality ratios that occurred during 2010–2020, and particularly after 2015, also correspond to the early initiation and viral suppression in the TasP era and the rapid PrEP scale-up among gay and bisexual men since 2016.^9^^,^^14^^,^^16^^,^^34^ In contrast, for females the number diagnosed not on treatment increased right up to 2015 before rapidly declining suggesting suboptimal guidelines based care for females.

Overall population level falls in HIV transmission have been associated with PrEP scale-up^12^ among gay and bisexual men which likely contributed to the recent sharp increase in the overall case detection rate. HIV testing among gay and bisexual men has also increased since 2010 due to a range of initiatives.^16^^,^^33^ However, this increase may have had a limited impact as the number of people living with an undiagnosed infection increased and the yearly diagnosed fraction and case detection rate only increased slightly prior to PrEP scale-up. Only 3206 Australian females have taken PrEP since it became available in 2018 (3.3% of people who have taken PrEP) but there has a been similar decline in new infections as for males. This suggests treatment as prevention has been effective in preventing HIV transmission among females with male partners living with HIV and that PrEP use in females has been effectively targeted to those most at-risk of infection.

These outcomes are likely due to the broad estimates masking different trends among priority populations. Diverging epidemics are becoming apparent particularly between Australian-born gay and bisexual men and those born overseas who often experience stigma and discrimination and may be less engaged with HIV education and counselling services and the healthcare system.^16^^,^^30^^,^^35^ We were unable to produce estimates and analyse trends for priority populations because many of the administrative datasets available have incomplete sexual behaviour, risk exposure, or country of birth data limiting their application for these populations.

Our analyses included the impacts of the COVID-19 pandemic. Australia's public health response to the pandemic led to falls in health service access, HIV testing, and PrEP use as well as a reduction in sexual partner numbers^13^^,^^14^^,^^36^^,^^37^ along with substantial changes to migration following the closure of Australia's national and several interstate borders during 2020–21. Many service interruptions were relatively short lived and the continuation of trends in the HIV treatment and suppressed virus steps suggests treatment continued to be effective at the population level in Australia. Following the re-opening of national borders there was a sharp rebound in the number of HIV notifications particularly for people previously diagnosed overseas. It is too early to determine if this increase is part of a return to pre-COVID trends or a shift to higher numbers of notifications, but it does signify the potential for increasing transmission and a growing population of people living with HIV in Australia, for whom ongoing treatment and access to care are crucial. We did estimate a fall in new infections, however given the fall in testing and the reliance on HIV notifications for producing many of the estimates, this trend should be treated with caution especially given the recent trends are based on only four years of data. This is also the period where the outputs from the ECDC HIV Modelling Tool are most uncertain.

Our analyses have limitations that need consideration. We used trends in general population migration data to inform emigration, but these do not necessarily reflect the movement patterns of people living with HIV with any level of granularity. This limitation was mitigated by calibrating the emigration rate to match the treatment coverage from a new HIV data linkage study. This study, currently underway, is linking data from the national HIV registry, Medicare, PBS, and death registries in Australia and will improve future estimates and enable us to conduct similar cascade and metric analyses for key populations (e.g. gay and bisexual men, migrants, people who inject drugs). We did not estimate the uncertainty in the HIV cascade gaps and our trend analysis was relatively simple using year as a single covariate and ignoring the uncertainty in the metric estimates. This was primarily done for simplicity and ease of interpretation while still producing robust results. The sudden change in the number of people diagnosed but untreated around 2011 should be interpreted with caution as this roughly corresponds to the time where the source of treatment data changes from the Australian HIV Observational Database (AHOD) to the PBS. Several metrics use the same underlying inputs, which can result in similar trends and change points, and rely on output from the ECDC model which are sensitive to changes in the underlying data from one year to the next and the underlying fitting parameters used. An advantage of having multiple metrics is they provide different viewpoints and enhance the understanding of the HIV epidemic. Despite these limitations, our Australian HIV Cascade estimates are consistent with other independent data sources. The ECDC model produces national estimates for the percentage undiagnosed that align with repeated bio-behavioural studies in 2014 and 2018.^38^ The cascade estimates for treatment and viral suppression also align closely with data for HIV-positive people attending sexual health clinics^16^ and the percentage of surveyed HIV-positive gay men reporting they are on treatment.^34^

To end HIV as a public health threat in Australia will require strategies to strengthen health promotion, PrEP scale-up, testing, and treatment among all key populations. Reducing late diagnoses and the pool of undiagnosed infections should be a focus, given they disproportionately contribute to new infections,^39^ as well as expanding access to PrEP. Even if the HIV targets are achieved there will be more than 30,000 people living with HIV after 2030 and ongoing immigration, meaning public health programs for HIV will need to be sustained and enhanced to ensure appropriate care and quality of life for people living with HIV and to avoid a reversal in progress.

## Contributors

RTG, RJG, and SM conceived the idea for the study. RTG developed the analysis code, conducted the formal analysis, and manages the online code repository. HM contributed to the statistical analysis. HM and GPC provided estimates from the HIV linkage study. JMK, KP, and SM contributed to the data curation with KP providing the data from the AHOD cohort. RTG, JMK, SM had access to the raw HIV notifications data. RTG and SM contributed to original draft preparation. JSR reviewed the results and the final manuscript and provided insights from a community perspective. RTG, HM, and AEG contributed to methodology. AEG chairs the Australian HIV Diagnosis and Care Cascade reference group. RJG and SM contributed to supervision and funding acquisition. All authors contributed to interpretation/validation, reviewing and editing, and read and approved the final version. RTG and SM verified all data and had the final responsibility for the decision to submit for publication.

## Data sharing statement

Australian State and Territory legislation prohibits the sharing of HIV notifications data used in this study. All cleaned publicly available data, reproducible code, and analysis documentation are available online.^19^

## Declaration of interests

RTG has received funding from Gilead Sciences for a presentation on the Australian HIV cascade. RTG's institution has received funding for his research from The World Bank Group, UNAIDS, NSW Department of Health, and the Australian Government Department of Health, Disability and Ageing. KP's institution has received unrestricted research grants from ViiV healthcare and Gilead Sciences. AEG has received speaker fees from Clinical Care Options and Seqirus, travel support from ViiV, in kind project support from GSK, and is a member of the Governing Council of the International AIDS society. RJG reports funding from Cepheid and SpeeDx towards the Australian Research Council Industrial Transformation Research Program Hub (ITRP) to Combat Antimicrobial Resistance grant (IH190100021). HM, JMK, GPC, JSR, SM have no interests to declare.
