# Supplementary material for: HIV cascade, key indicators, and other epidemiological metrics in Australia (2004–2023): a retrospective analysis
Source: Lancet Reg Health West Pac. 2026 Jun 16;71:101905. doi: 10.1016/j.lanwpc.2026.101905 (PMC13293760; doi:10.1016/j.lanwpc.2026.101905)
Supplement: Supplementary Appendix [file mmc1.docx]

Supplementary Appendix

**HIV cascade, key indicators, and other epidemiological metrics in Australia (2004-2023): a retrospective analysis**

Richard T. Gray PhD^1^, Hamish McManus PhD^1^, Jonathan M. King PhD^1^, Gladymar Pérez Chacón PhD^1^, John S. Rule PhD^2^, Kathy Petoumenos PhD^1^, Andrew E. Grulich PhD^1^, Rebecca J. Guy PhD^1^*, Skye McGregor PhD^1^*

1. Kirby Institute, UNSW Sydney, NSW 2052, Australia

2. National Association for People with HIV Australia (NAPWHA), Newtown NSW 2042 Australia

* Contributed equally as senior authors.

**Contents**

[1. Detailed methods and data sources used to calculate the HIV cascade 2](#_Toc230956842)

[1.1. Number of people living with diagnosed HIV 2](#_Toc230956843)

[1.2. Number of people living with HIV and the number undiagnosed 5](#_Toc230956844)

[1.3. Number of people retained in care 6](#_Toc230956845)

[1.4. Number of people living with HIV on antiretroviral therapy (ART) 6](#_Toc230956846)

[1.5. Number of people with a suppressed viral load 8](#_Toc230956847)

[1.6. Calibration and validation of number with diagnosed HIV 8](#_Toc230956848)

[2. Annual estimates for the Australian HIV cascade and related metrics 11](#_Toc230956849)

[2.1. Overall population estimates 11](#_Toc230956850)

[2.2. Male estimates 14](#_Toc230956851)

[2.3. Female estimates 18](#_Toc230956852)

[3. Statistical analysis of trends 22](#_Toc230956853)

[3.1. Analysis results for males 23](#_Toc230956854)

[3.2. Analysis results for females 30](#_Toc230956855)

[4. Regression model fitting 36](#_Toc230956856)

[4.1. Figures for the overall population 36](#_Toc230956857)

[4.2. Figures for males 55](#_Toc230956858)

[4.3. Figures for females 74](#_Toc230956859)

[5. Software used 93](#_Toc230956860)

[6. STROBE checklist for observational studies 95](#_Toc230956861)

[7. References 105](#_Toc230956862)

This supplementary document provides further data and methodological details for calculating the estimates for the HIV cascade, key indicators, and other epidemiological metrics for each population, as well as additional results from the analysis. All computer code used to produce the results and figures for this study are available online This code is part of a larger code base used to produce annual estimates for Australian diagnosis and care cascades for HIV, hepatitis C virus (HCV), and bacterial sexually transmitted infections (STIs). These estimates are for national surveillance, and the code has been constantly updated over the last 10 years as new data becomes available and based on feedback from a reference group of research, government and community representatives. As noted in the repository README file this code has been used to produce results for multiple reports and papers. Within this repository there is a folder for the HIV cascade code, publicly available data and summary results and figures. A specific release has been generated and linked to the code used for this paper: https://github.com/The-Kirby-Institute/Cascade_calculations/releases/tag/v5.95_HIV_cascade_paper which has then been archived at: https://zenodo.org/records/10324612. Updates to the code and calculation results are available from: https://github.com/The-Kirby-Institute/Cascade_calculations/.^1^ Details of the specific scripts, software, and packages used are provided in Section 5.

# Detailed methods and data sources used to calculate the HIV cascade

The following sections provide additional methodological details for the HIV cascade estimates and the data sources used in the calculations outlined in the main manuscript. The approach taken to develop the HIV diagnosis and care cascade was informed by recommendations from a national stakeholder reference group (see Acknowledgments in the main text). To produce estimates for the HIV cascade for each population we first calculated the estimated number of people living with diagnosed HIV and the number taking antiretroviral therapy (ART). From the estimates for these two steps, we then calculated the number of people living with HIV, the number retained in care, and the number with viral suppression.

## Number of people living with diagnosed HIV

To estimate the number and range of people living with diagnosed HIV each year (shown in Figure S1A) we performed a calculation using the annual notifications, estimated mortality rates and emigration rates data for each population. We estimated the annual number of HIV diagnoses using annual HIV notifications data from Australia’s National HIV Registry. Australia’s HIV surveillance system involves mandatory reporting by doctors and laboratories of all HIV diagnoses, including CD4 count at diagnosis, to state and territory health departments who submit these data to a National HIV Registry.^2^ For each population, we estimated the annual number of people diagnosed using all the notifications recorded in the registry for that population to the end of 2023 including people previously diagnosed overseas. Notifications are incomplete for several key variables used in the cascade calculations. Missing data was filled using multivariate imputation with the MICE R package (version 3.16.0)^3^ and the variables: year of diagnosis (complete), state or territory (complete), region of diagnosis within Australia (26.4% missing overall; 3.3% missing since 2004), age when diagnosed (0.41% missing overall), gender (0.54% missing overall, only n =6 since 2004), mode of exposure group (13.0% missing overall; 3.5% missing since 2004), and country of birth (26.2% missing overall; 1.4% missing since 2004). Twenty imputed data sets were produced for each Australian state and territory separately for each population and then merged to prevent inconsistencies between region of diagnosis and state. The calculation method described in the following paragraphs was then applied to each of the 20 imputed sets with the annual mean and range calculated from each of the sets to produce the final annual estimates for the number of people living with diagnosed HIV for each population.

Due to incomplete or inaccurate recording of name codes the registry contains multiple reports for some individuals, especially during the early stages of the epidemic. To estimate the number of duplicate notifications we used a statistical technique which has previously been applied to Australia's National HIV Registry.^4^ This calculation uses date of birth of notifications and the assumption that multiple reports of the same case had the same date of birth. We estimated the number of duplicate notifications annually up to 2016 resulting in 8.1% of all notifications being duplicates by 2016 with most duplicates occurring early in the epidemic. We assumed all notifications were unique from the beginning of 2017. A detailed analysis of duplicates in Australian notifications using this method is provided in the online code repository (file: https://github.com/The-Kirby-Institute/Cascade_calculations/HIV/0-DuplicateAnalysis.Rmd; associated document: ~/HIV/docs/Duplicate_analysis.docx).^1^ Figure S1B shows the cumulative number of unique notifications for the overall population between 2004–2023. This statistical technique was applied to the notifications of each population separately.

We used two datasets to estimate the number of deaths among people diagnosed with HIV. To estimate the number of deaths up to 2003 we used a linkage study conducted between Australia’s National Death Index and the National HIV Registry for cases to the end of 2003.^5^ That study estimated the number of HIV‑ and AIDS‑related deaths and calculated standardized mortality ratios for people with HIV during different eras of antiretroviral therapy. It identified 8,519 deaths among people diagnosed with HIV or AIDS by the end of 2003. Of these deaths, 6,900 were already recorded in the National HIV Registry, meaning that 19% of all deaths were missing from the registry. Due to the backdating of deaths in the National HIV Registry after 2003, we inflated the number of recorded deaths in the registry until the end of 2003 by 19% [inflating the 7,102 deaths recorded to the end of 2003 to 8,768 = 7,102/(1-0.19) deaths overall] and estimated the overall average mortality rate for diagnosed people living with HIV prior to 2003. The number of annual deaths was then estimated by inflating the recorded deaths in the registry each year using the estimated annual mortality rate to track the change over time and ensure the total number of deaths matched the 8,768 estimated by the end of 2003. After 2003 we calculated crude annual mortality rates and their 95% confidence intervals (CIs) using data from the Australian HIV Observational Database (AHOD).^6^ Between 2004 and 2023, similar annual mortality rates were estimated for the AHOD cohort regardless of whether people were retained, lost or returned to follow‑up. We applied the annual mortality rates from AHOD for the period 2004–**–**2023 (using the estimate and 95% CI as a range) to calculate the annual number of deaths among diagnosed people living with HIV (overall population estimated shown in Figure S1C with the estimated mortality rate shown in Figure S1D). Separate mortality rates for males and females were estimate applying the relative ratio of the general population standardised death rates for males and females to the overall mortality rate.We also considered the impact of migration. Some people living with HIV in Australia will have previously received an HIV diagnosis in another country. These people only enter the National HIV Registry and become officially notified when they receive a confirmatory diagnosis in Australia. Confirmation of HIV status is routinely performed at point of entry into clinical care, and the notification record includes information on previous diagnosis overseas—which is primarily obtained through self-report. We assumed people living with HIV in Australia who have been previously diagnosed overseas are aware of their HIV status and hence part of the diagnosed population (even if they are not in care or have not been notified within Australia). People who enter Australia with undiagnosed HIV are part of the undiagnosed population. As people are not included in the National HIV Registry until they have been diagnosed in Australia (even if they have been diagnosed previously overseas) we are unable to include the entry of all people living with diagnosed HIV. This limitation will result in only a small underestimate of the number living with diagnosed HIV given most people previously diagnosed overseas will enter care in Australia quickly to be able to access treatment.

The level of emigration of people living with diagnosed HIV is highly uncertain as there are no data in Australia informing the number of people living with HIV who emigrate each year—other than follow‑up data of people recently diagnosed in the Australian state of New South Wales (NSW) since 2013.^7^ NSW Health has followed up all people diagnosed with HIV during 2013–2022 and reported up to 5% of people moved overseas soon after their diagnosis with most of these cases occurring in people born overseas. Up until the end of 2019 the post-diagnosis emigration percentage remained relatively constant, and we assumed it has been constant over time since the start of the epidemic. To the end of 2019 we reduced the number of new diagnoses by a weighted percentage using the cumulative proportion of notifications in Australian born versus overseas born people and the associated percentage from the NSW data. There was a sharp change in emigration patterns over 2020–2022 due to the closure and re-opening of national borders in response to the COVID-19 pandemic. We calculated and applied separate post-diagnosis emigration rates from the NSW health data for the 2020–2021 and 2022–2023 periods. We assumed males and females had the same post-diagnosis emigrations as for the overall population as the NSW data was not stratified by sex.

In terms of emigration of people living with HIV over the long term, we estimated an emigration rate for diagnosed people living with HIV using data for the overall population from the Australian Bureau of Statistics (ABS). As there are likely to be people living with HIV who leave temporarily and then return to Australia (some of whom may still receive care and treatment while overseas), we used data on the annual number of people in the overall population who permanently leave Australia (provided by the ABS between 1976–2016 in the archived release for June 2017; data series 340102, Table 2)^8–10^ and the estimated resident population from the ABS (previously data series 3101059)^11^ to calculate an overall annual emigration rate. Since 1981 this rate has risen from around 0.1% to 0.4% of the resident population leaving Australia permanently. Since June 2017, the ABS no longer records permanent removals due to the removal of the physical green card form from the customs process upon leaving Australia. To estimate the permanent removal rate, we used Net Overseas Migration (NOM) emigration data from the ABS (previously data series 34120d0001; now available from the ABS Data Cube).^9,10^ A comparison between NOM emigration for Australian Citizens and permanent removal numbers for the years 2010–2016 showed a close alignment (see Figure S2). We therefore used the Australian citizen NOM departures data for the years 2017–2023 to estimate permanent removals and the migration rate for people living with diagnosed HIV for this period. The resulting overall population migration rate was further adjusted to reflect the different emigration rates for males and females older than 15 years in the general population given most people living with HIV in Australia are adult males. The adult male and female migration rates were used for the male and female HIV cascade calculations respectively.

Given the uncertainty in the emigration rate for people living with diagnosed HIV, we made a final adjustment to the overall permanent rate of departure for each population by applying a multiplier to the emigration rate. This multiplier was manually calibrated so that the estimated HIV treatment coverage in 2015 from the cascade calculations aligned with the 2015 treatment percentage from recently available HIV linkage data (provided by author H. McManus and G. Pérez Chacón). We used the 2015 estimates as they precede the HIV pre-exposure prophylaxis (PrEP) scale-up and the COVID-19 pandemic. We then compared the percentage retained in care and the percentage on ART from the cascade estimates between 2014––2023 to the corresponding linkage data for validation. See Section 1.6 below for further details of the linkage data and the calibration and validation process.

The permanent rate of departure represents the lower bound of the overall rate at which Australian residents leave the country for more than 12 months. However, diagnosed people living with HIV require ongoing care and treatment which is not subsidised in many countries, so we assumed the trends in the permanent rate of departure are a reasonable proxy for the trends in emigration of diagnosed people living with HIV. Overall, we assumed a range in the annual emigration rate between zero and double the calibrated rate.

Our overall estimate of the number of people diagnosed with HIV in Australia each year is obtained by adding the number of unique notifications to the previous year’s estimate and subtracting the number of deaths and emigrants using the mortality and migration rates applied to the previous year’s estimate.

**Figure S1: Calculation of number of people living with diagnosed HIV.** Trends for each part of the calculation of the overall number of people living with diagnosed HIV in Australia between 2004– 2023. A) Estimated number of diagnosed people in the Australian. B) Estimated number of cumulative unique HIV notifications at the end of each year. C) Cumulative number of deaths among people with diagnosed HIV. D) The estimated mortality rate for people living with HIV. E) The cumulative number of people living with HIV who have emigrated at the end of each year. F) The estimated emigration rate for people living with HIV. The red line shows the estimate with the shading showing the range.


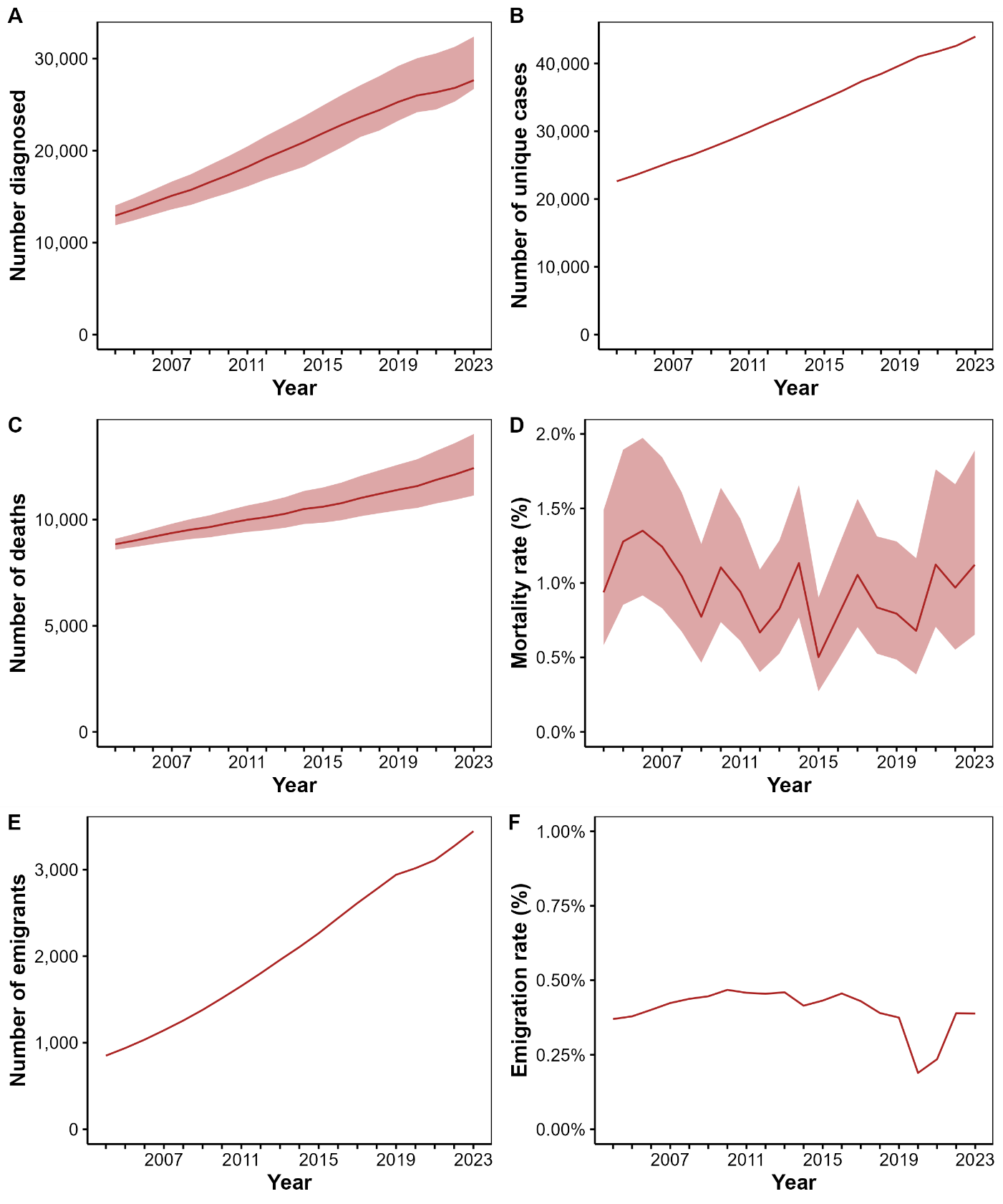


**Figure S2:** **Data for estimating the overall baseline emigration rate of people living with HIV.** Comparison of annual permanent removals and Australian citizen net overseas migration (NOM) departures between 2004–2023.

**
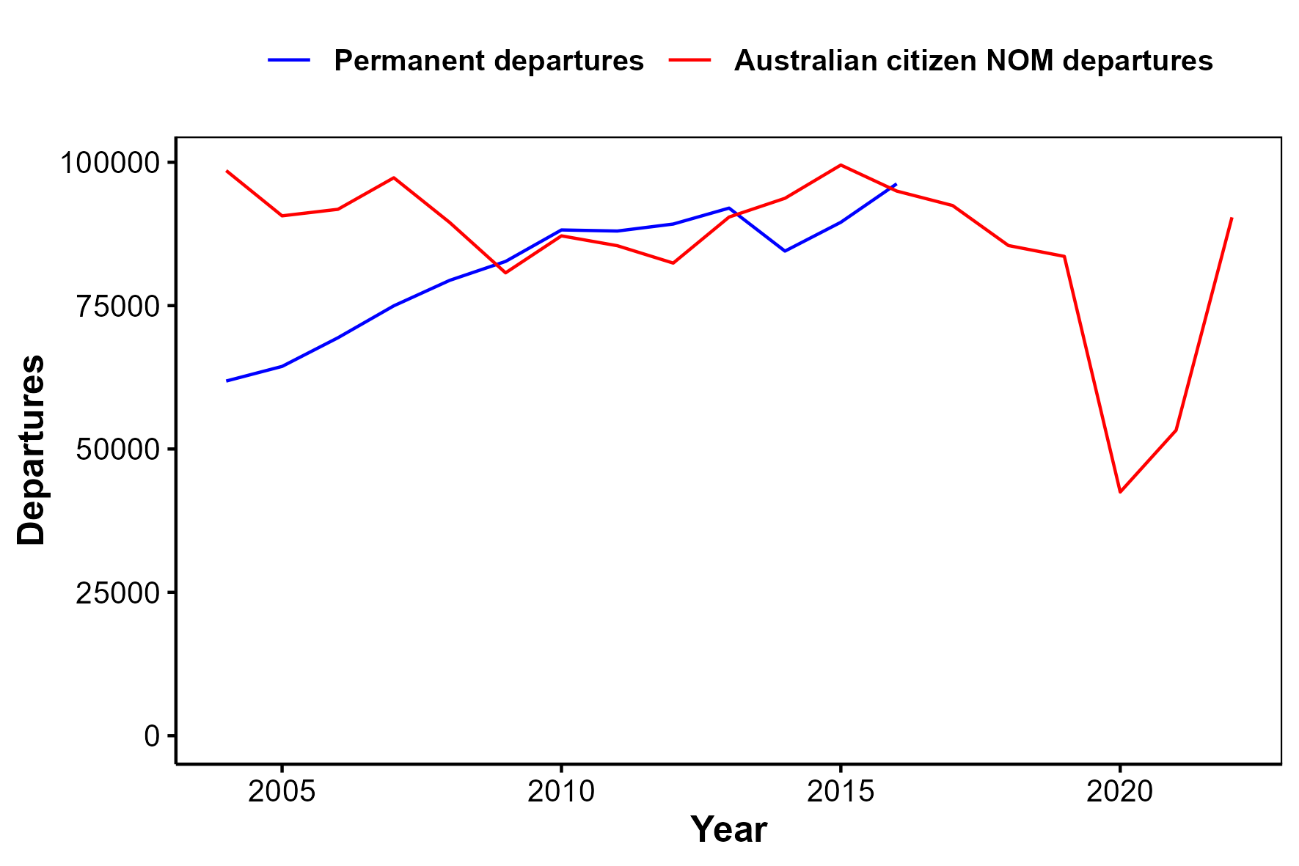
**

## Number of people living with HIV and the number undiagnosed

To estimate the overall number of people living with HIV, both diagnosed and undiagnosed, in each population we used the European Centre for Disease Prevention and Control (ECDC) HIV Modelling Tool (version 1.3.0) to estimate the proportion of people with HIV who are undiagnosed.^12,13^ While the ECDC tool also estimates the number living with diagnosed HIV we used our calculated estimate because it is based directly on empirical data rather than being estimated through the ECDC tool’s fitting procedure.

The ECDC tool is a multi‑state back‑calculation model using notifications data and estimates for the rate of CD4+ cell count decline to fit notification rates over time, producing estimates for HIV incidence, time between infection and diagnosis, and the undiagnosed population by CD4+ cell count strata, using surveillance data on new HIV and AIDS notifications. People living with HIV in Australia who previously received an HIV diagnosis overseas only enter the National HIV Registry and become officially notified when they receive a confirmatory diagnosis in Australia. This routinely occurs at the point of entry into clinical care. The notification record includes information (usually self-reported) on previous diagnosis overseas.^2^ We assumed people living with HIV in Australia previously diagnosed overseas are aware of their HIV status and hence part of the diagnosed population. To estimate annual new infections and the proportion diagnosed we used the ECDC tool with previously diagnosed overseas excluded from diagnoses. We also excluded people who had evidence of seroconversion during the previous 12 months as we assumed they acquired HIV and were diagnosed within the year. This approach could still overestimate new infections if a substantial proportion of people who acquired HIV overseas are first diagnosed in Australia and categorized as acquiring HIV in Australia.^14^

To run the model, notifications data for each population (excluding those previously diagnosed overseas, excluding those with evidence of seroconversion, and adjusted for duplicates) were divided into those attributed to male-to-male sex (representing gay and bisexual men), heterosexual contact, injecting drug use, and ‘other’ risk exposures. Notifications were then categorised by CD4+ cell count strata, whether the person had AIDS at the time of diagnosis to produce the required input files for the tool. For the death and emigration inputs we used the estimated number of deaths and emigrants from our calculations of the number of diagnosed people living with HIV. Deaths were distributed across the exposure groups in proportion with the number of notifications for the population. The tool's diagnosis rate options were adjusted for each population to best fit the CD4 count at diagnosis data (determined by visual inspection) using the default time intervals up to 2020 and separate intervals for the 2020–2022 and 2023 periods to reflect potential changes in testing during the acute phase and post-acute phase of the COVID-19 pandemic. We assumed diagnosis rates were continuous, different by CD4 count category and could change during the time interval. Other ECDC tool options and parameters were left at their default settings except for the incidence curve knot count. We set this parameter to three to produce a more gradual change in the number of new infections which we deemed to be more realistic.

Estimates and uncertainty ranges (given by 95% CIs used as the range) for the proportion undiagnosed were obtained using 100 bootstrapped fits produced by the ECDC Tool. The prevalence of HIV in Australia among each population was then estimated by inflating the calculated number of people living with diagnosed infection by the estimated level of undiagnosed infection. The number of people with HIV and the number undiagnosed were then obtained by applying the proportion undiagnosed to the estimated number living with diagnosed HIV for each year. The lower and upper values of the proportion undiagnosed were applied to the lower and upper range for the number living with diagnosed HIV to obtain the uncertainty range in the number undiagnosed. For validation we compared the model estimates for undiagnosed gay and bisexual men with empirical data from the COUNT study.^15^ This study was conducted alongside routine behavioural surveillance surveys of gay and homosexually active men from Sydney, Melbourne, Canberra, and Perth recruited from gay community sites in 2013–2014. In that study, 8.9% of participants were previously undiagnosed with HIV (95% CI 5.8–13.5%). This estimate and range overlap the estimated percentage undiagnosed in 2014 for gay and bisexual men of 11.1% (range: 9.3–13.2%) from the ECDC HIV Modelling Tool.

## Number of people retained in care

For annual surveillance an estimate for the number of people living with HIV retained in care is also reported. The estimates for this step have only been produced since 2013 and are calculated using the results from a clinical study and its follow-up study. This means we were unable to analyse the trend for this step of the HIV cascade and only reported the 2023 estimate in the main text. Here we describe the methods to estimate this step and provide estimates since 2013 (see Table S1).

To estimate the number of people living with HIV retained in care, we used available clinical data on the proportion of HIV-positive people attending a clinic who receive an annual CD4 or viral load test. An issue with clinic data is people can appear to be lost to follow-up, and hence not in care, when they have just transferred to another clinic. A study conducted during 2011–2013 in a network of the six main HIV clinical care sites in the Australian state of Victoria estimated 91.4–98.8% of people living with HIV were retained in care^16^. This estimate was obtained by cross-referencing of clinical data between sites and phone tracing individuals who had accessed care between February 2011 and June 2013, but who had not accessed care between June 2013 and February 2014. Follow-up studies were conducted during 2016–2017 and they obtained results agreeing with the earlier study with approximately 96% of people retained in care.^17^ We assumed these results are broadly representative of people living with diagnosed HIV in Australia and assumed a best estimate of 96% retained in care with a range equal to 93% to 99% for each population. A limitation of these studies for estimating retention in care is that it only considers people who were already in care at some point in the past and potentially misses those who have not been in care since diagnosis.

## Number of people living with HIV on antiretroviral therapy (ART)

People living with HIV who have received ART at least once during the calendar year are classified as on treatment. This is different to the UNAIDS Indicator Registry definition which uses on ART at the end of the year.^18^ Our definition will produce robust estimates for the number people on treatment at the end of the year as people living with HIV in Australia can get multiple scripts during a single clinician visit which provide treatment for potentially up to 12 months meaning such a person would be on treatment at the end of the calendar year.

The Australian government provides fully subsidised ART to people living with HIV eligible for a Medicare (Australia’s publicly funded universal health care system) card through the Pharmaceutical Benefits Scheme (PBS)^19^—which records all script claims processed by the health scheme within Australia. To estimate the number of people on ART between 2004–2023 we combined estimates from multiple data sources as PBS data were not available prior to 2014. For 2004–2013, estimates for the number of unique patients who received treatment for HIV were estimated from the AHOD cohort (which includes temporary residents ineligible for Medicare) and were reported in the Annual Surveillance Report^2^. For the period since 2014, we estimated the number of people receiving antiretroviral therapy using a 100% longitudinal dataset of PBS patient‑level script claims data provided by the Australian Department of Health and Aged Care. It includes all PBS‑listed drugs with HIV indications and is stratified by sex. A challenge with the PBS data is that five drugs licenced for treating HIV are also used for hepatitis B, HIV PrEP, and HIV post-exposure prophylaxis (nPEP) which need to be separated from HIV treatment. For our estimates we excluded all people who are only prescribed TDF/FTC, TDF or 3TC. In addition, we excluded people who were only prescribed DOL or RAL with one of TDF/FTC, TDF or 3TC less than two times per year. Our resulting estimate is the number of unique patients in the remaining PBS data who filled in at least one script for HIV treatment in the 12 months prior to the end of December each year. This 12-month window potentially misses some people on treatment due to the timing of the filling of scripts. Given this uncertainty we estimated an upper bound for the number on ART in an 18-month window period.

To the PBS estimate we added an estimate for the number of people living with HIV in Australia ineligible for Medicare and taking ART. As these people are ineligible for Medicare, they cannot access government subsidised ART and any HIV treatment they obtain outside Medicare is not recorded in the PBS data. In the past a major source of HIV treatment for people ineligible for Medicare are pharmaceutical company compassionate access schemes. Collation of data from these schemes provided annual estimates for the number of people living with HIV ineligible for Medicare in Australia taking ART up to the end of 2020.^20,21^ In 2022, a new program administered by national and state/territory health departments was established to provide all people living with HIV ineligible for Medicare access to subsidised ART. Data provided by the Australian Department of Health estimates 1500––1600 people have enrolled in the program since its initiation by June 2023. This increased to a reported 2501 people by June 2024. We assumed there were 1550 people overall living with HIV ineligible for Medicare taking ART at the end of 2022 and 2501 at the end of 2023. We used the average of the 2020 and 2022 estimates for 2021. The final estimates used are shown in Figure S3. This data for people ineligible for Medicare is not stratified by sex. To estimate the number of males and females ineligible for Medicare on ART, we used the proportions of participants in the ATRAS study who were male (73.9%) and female (26%).^22^

To merge and validate the estimates from AHOD and the 100% PBS plus ineligible for Medicare data for the overall population we first obtained a separate estimate for the number treated in 2013 (the year between the AHOD and 100% PBS data) using a 10% longitudinal sample of the PBS prescription claims data provided to the Kirby Institute by the company Prospection <http://www.prospection.com.au/>.^23^ This data is a randomised individual level, de-identified script claims data set. The data had over 170 million script claims and over 3 million patients. The data became available from 2006 but has only been complete since June 2013. It includes all PBS listed drugs with HIV indications. Our estimate for 2013 is then the number of unique patients in the PBS data who filled in at least one script in the 12 months prior to the end of December each year multiplied by 10. We assumed that 10% of the Australian population were sampled to estimate the uncertainty range as a 95% confidence interval (which equates to approximately $\pm$ 5%). We found the 10% longitudinal sample was within 2% of the full sample.

**Figure S3: Overall number of people living with HIV on ART who are Medicare ineligible.** Estimates for the number of people ineligible for Medicare on ART between 2013–2023. Note 2021 estimate is the average of the 2020 and 2022 estimates because there is no data for 2021.


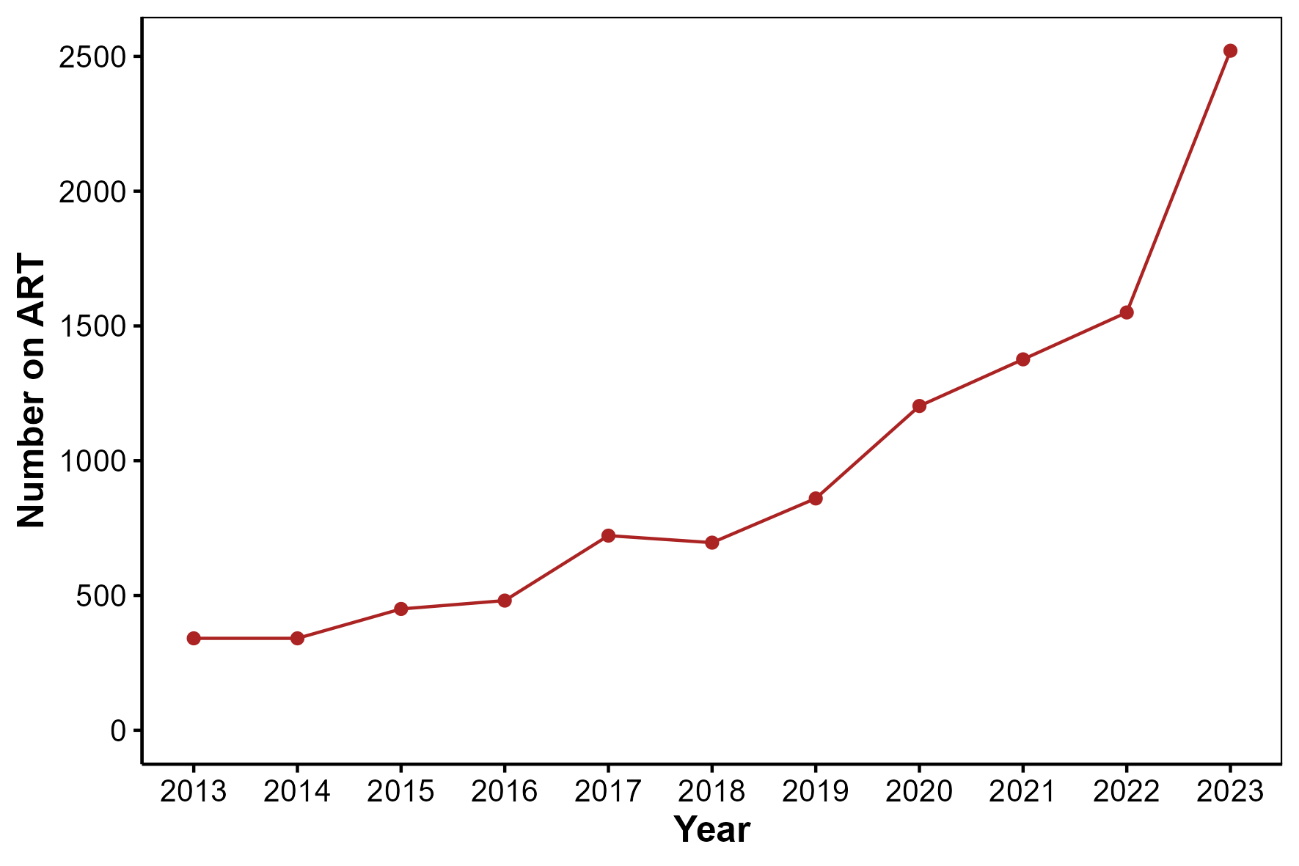


We joined the annual PBS and AHOD derived estimates for the number on ART by exploring various fits to the treatment estimates between 2000–2020 as shown in Figure S4. A detailed description of the ART fitting methods is provided in the online code repository (code file: ~/HIV/0-ArtAnalysis.Rmd; associated document: ~/HIV/docs/ART_analysis.docx).^1^ A logistic curve fit using weighted non-linear least squares favouring the 2014–2020 PBS estimates (which are assumed to be more accurate) produced the best fit and showed the treatment estimates from AHOD aligned with the PBS derived estimates except for the years 2011–2013. The AHOD estimates for this period are likely underestimates due to the migration to an online reporting system resulting in inconsistences in the data and an inability to make robust adjustments.^2^ For the final cascade estimates, we used the AHOD data up to 2010 and the PBS estimates from 2013 with the fitted estimates used for 2011 and 2012. To obtain a lower and upper bound for the number on ART prior to 2013 we produced 10,000 samples from the fitted logistic curve parameter ranges (shown in Figure S4C).

**Figure S4: Number of people living with HIV on ART merging of datasets.** Fitting to ART trends over time. A) Various linear regression fits to the AHOD and PBS ART estimates for 2000–2014. B) Exponential versus logistic fit to the data weighted towards the PBS data. C) Ensemble of logistic fits sampled from the parameter 95% CIs of the best fitted curved used to estimate the number on HIV treatment between 2011–2013. Note the shaded 95% interval and IQR in the legend are the sampled 95% uncertainty range and interquartile range for the sampled curves.


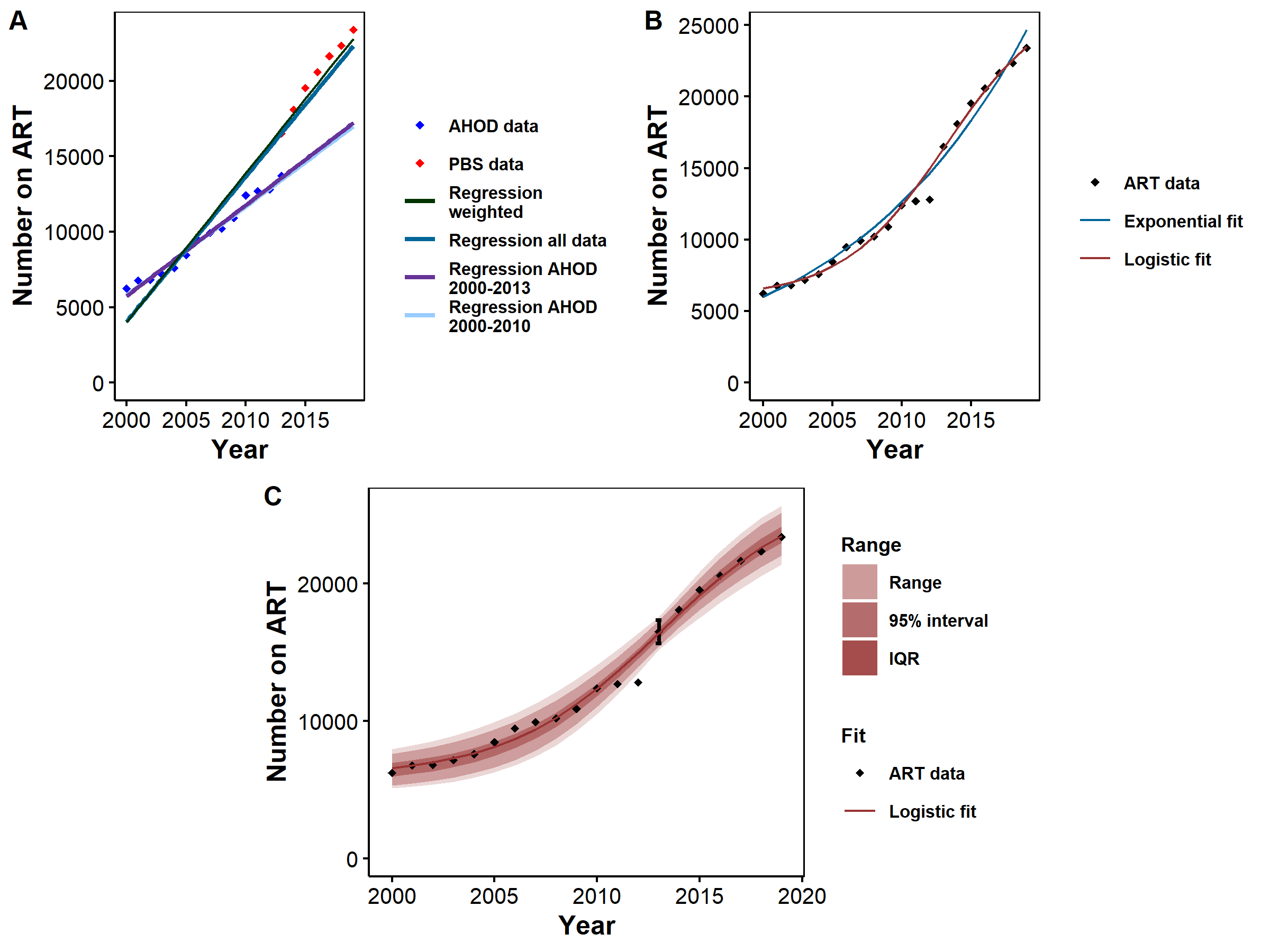


## Number of people with a suppressed viral load

We defined virological suppression of HIV as less than 200 viral copies per ml. We used this threshold rather than a threshold of < 50 or < 1000 copies/ml (as used by UNAIDS)^18^ because it was considered by our Australian HIV Diagnosis and Care Cascade National Reference Group to be the most clinically relevant threshold for the care of people living with HIV and is the threshold used in Australia’s National HIV strategy.

We took the proportion of people in each population on ART with viral suppression to be the proportion of patients recorded in AHOD each year who had less than 200 copies per mL at their last viral load test during the year. Uncertainty bounds were estimated by calculating the 95% confidence interval for this proportion. Multiplying this proportion by the number on ART produced the estimated number of treated people with a suppressed viral load.

## Calibration and validation of number with diagnosed HIV

A recently initiated HIV linkage study was able to provide data to calibrate the emigration rate multiplier for the population overall, males, and females used in the calculation of the number of people living with diagnosed HIV. As described in section 1.1 this multiplier was applied to the corresponding general population emigration rate. This study linking HIV notifications, PBS data, Medicare Benefits Scheme (MBS) data, and National Death Registry is still being conducted but was able to provide overall summary data for the overall population and males and females diagnosed with HIV and on treatment during the period 1997–2020. HIV notifications since 1997 form the foundation or spine of the linkage and people notified with HIV are assumed to still be living in Australia at the end of each year if they have not died prior to the current calendar year and who have recorded activity in any of the linked data sets in the current or preceding year. Linked individuals are assumed to be retained in care if there is recorded MBS or PBS activity in the current or preceding year and assumed to be on ART if there is recorded PBS activity for HIV drugs in the current or preceding year. When extracting this data, a broad definition of ART was used (in line with the definition for HIV treatment in Section S1.4) with ART regimens only excluded if they are indicated for HIV PrEP based on PBS codes. To estimate the percentage retained in care and on treatment we only considered people in the dataset with a high linkage strength. The aggregate numbers for each year between 2013–2020 are shown in Table S1 below. These were provided by authors H. McManus and G. Pérez Chacón with approval from the data custodians of the HIV linkage study—the Australian Department of Health and Aged Care and the Australian Institute of Health and Welfare. Ethics approvals for the HIV linkage study were obtained from the Australian Institute of Health and Welfare Ethics (EO2019/2/1031), UNSW Sydney (HC180815), NSW Population and Health Services Research Committee (2019/ETH13288), Aboriginal Health and Medical Research Council (1473/18), and the ACT Health Human Research Ethics Committee (HC180815).

**Table S1: HIV linkage study data for calibration of emigration rate.** Aggregate data from the HIV linkage study (provided by authors H. McManus and G. Pérez Chacón) used for calibration and validation of the HIV cascade estimates*.

| **Year** | **Number linked** | **Number with MBS or PBS activity (retained in care)** | **Number with PBS activity (on ART)** | **Percent retained** | **Percent on ART** |
| --- | --- | --- | --- | --- | --- |
| **Overall** |  |  |  |  |  |
| 2015 | 14,665 | 13,717 | 12,890 | 93·5% | 87·9% |
| 2016 | 15,499 | 14,618 | 13,849 | 94·3% | 89·4% |
| 2017 | 16,242 | 15,391 | 14,558 | 94·8% | 89·6% |
| 2018 | 16,864 | 16,072 | 15,001 | 95·3% | 89·0% |
| 2019 | 17,526 | 16,785 | 16,105 | 95·8% | 91·9% |
| 2020 | 17,776 | 17,206 | 16,673 | 96·8% | 93·8% |
| **Males** |  |  |  |  |  |
| 2015 | 12,870 | 12,118 | 11,390 | 94·2% | 88·5% |
| 2016 | 13,599 | 12,908 | 12,240 | 94·9% | 90·0% |
| 2017 | 14,224 | 13,539 | 12,824 | 95·2% | 90·2% |
| 2018 | 14,763 | 14,116 | 13,183 | 95·6% | 89·3% |
| 2019 | 15,310 | 14,712 | 14,133 | 96·1% | 92·3% |
| 2020 | 15,501 | 15,046 | 14,604 | 97·1% | 94·2% |
| **Females** |  |  |  |  |  |
| 2015 | 1,768 | 1,577 | 1,481 | 89·2% | 83·8% |
| 2016 | 1,870 | 1,686 | 1,589 | 90·2% | 85·0% |
| 2017 | 1,980 | 1,822 | 1,709 | 92·0% | 86·3% |
| 2018 | 2,064 | 1,924 | 1,792 | 93·2% | 86·8% |
| 2019 | 2,173 | 2,037 | 1,940 | 93·7% | 89·3% |
| 2020 | 2,230 | 2,120 | 2,034 | 95·1% | 91·2% |

* Methods presented at the 2025 Australian Society of HIV Medicine (ASHM) Conference: Miller et al, National Data Linkage for HIV Surveillance: Enhancing routine surveillance systems to inform progress with national strategy indicators. Poster presentation. ASHM HIV & AIDS Conference, Adelaide, 15th-18th September 2025.

This emigration rate multiplier was manually calibrated so that the estimated HIV treatment coverage between 2015–2020 from the cascade calculations aligned with the 2015–2020 treatment percentage from the HIV linkage data. We used the data from 2015 because it is after the first year of 100% PBS data (2014). In 2015, the linkage data estimates 88.7% of all people diagnosed in Australia were on ART. To match this estimate, the overall population emigration rate needed to be multiplied by a constant factor of 1.46. This means the estimated emigration rate for people living with HIV is relatively 46% higher than the permanent emigration rate for the general population. For 2023, this corresponds to a change in the percentage of people who emigrate from ~0.34% to ~0.50% which equates to ~103 people to ~150 people moving overseas). We repeated this process for the male and female population living with HIV. To fit the linkage data, we had to allow the multiplier to vary over time. We assumed a linear change from 2015 to 2020. For males the multiplier increased from 1.1 to 3.3, suggesting emigration increased during this period. In contrast for females, the multiplier decreased substantially from 2 to -5, suggesting more females living with HIV entered Australia than left (resulting in a negative emigration rate). While the changes in the emigration rate were quite large, we obtained good fits to the linkage treatment data (as shown in the column of Figure S5). The remaining data for percentage retained in care between 2014–2020 and percentage on ART between 2021–2023 was used to validate the cascade calculation estimates. Note the linkage data only includes Medicare eligible people whereas our cascade calculation estimates for diagnosed people and those on ART includes people ineligible for Medicare. Calibrating to the linkage data implicitly assume that people ineligible for Medicare have the same ART percentage as Australian residents. It is likely that people ineligible for Medicare have a lower ART coverage, however, they only make up a small proportion of all people living with HIV in Australia and hence this assumption will only result in a small error, well within the uncertainty range for the number diagnosed overall.

**Figure S5:** **HIV linkage study data for calibration of emigration rate**. Comparison of ART coverage and retention in from the overall cascade estimates (A and B), male cascade estimates (C and D), and female cascade estimates (E and F) and the HIV linkage study (Table S1). The HIV linkage estimates represent the calibration target for the emigration rate multiplier which adjusts the overall population emigration rate as described in Section 1.6. The two black points and whiskers in B, D, and F represent the overall point estimates and 95% confidence intervals from the clinical follow-up studies described in section 1.3. Note sub-figures D and F show the overall retained data rather than male and female specific data.

| A | 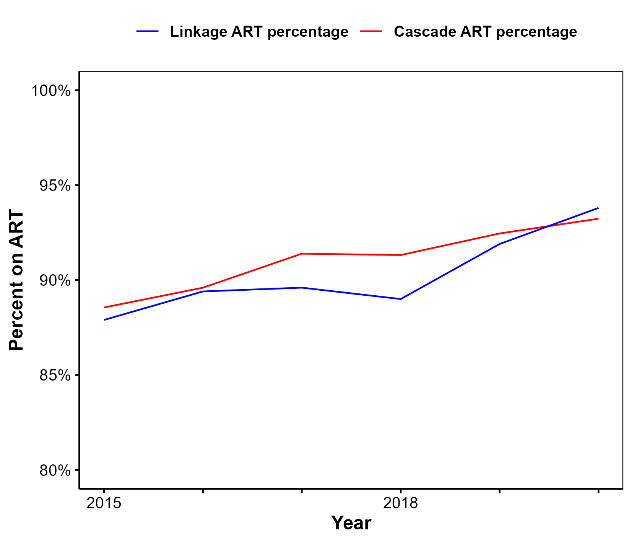 | B | 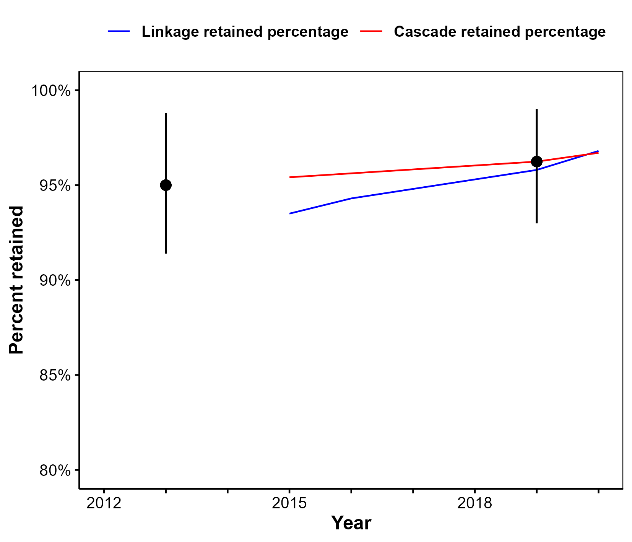 |
| --- | --- | --- | --- |
| C | 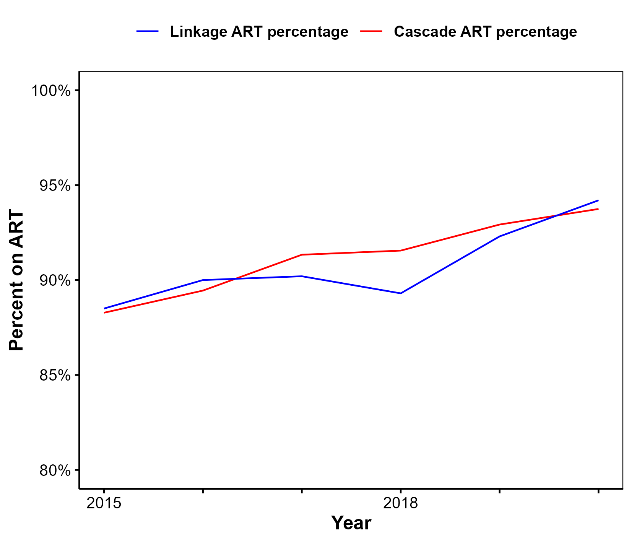 | D | 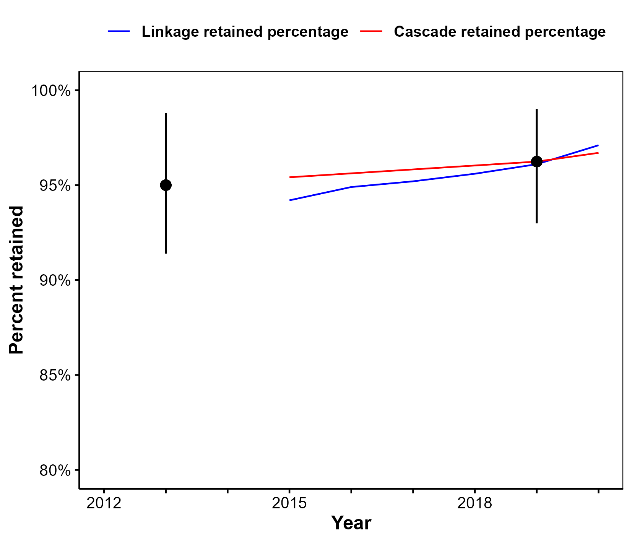 |
| E | 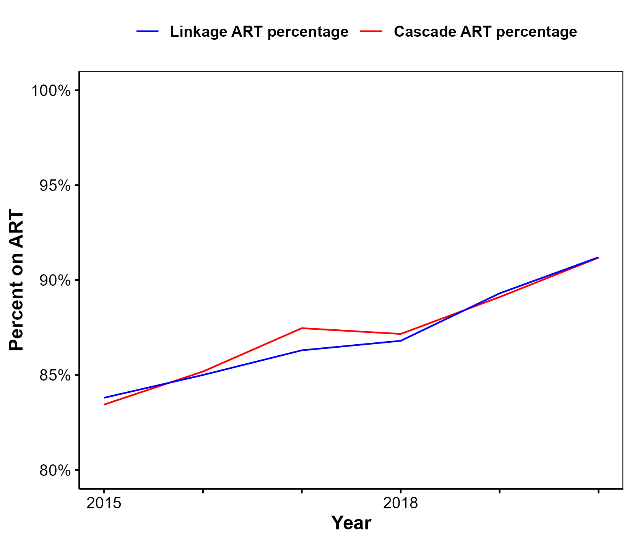 | F | 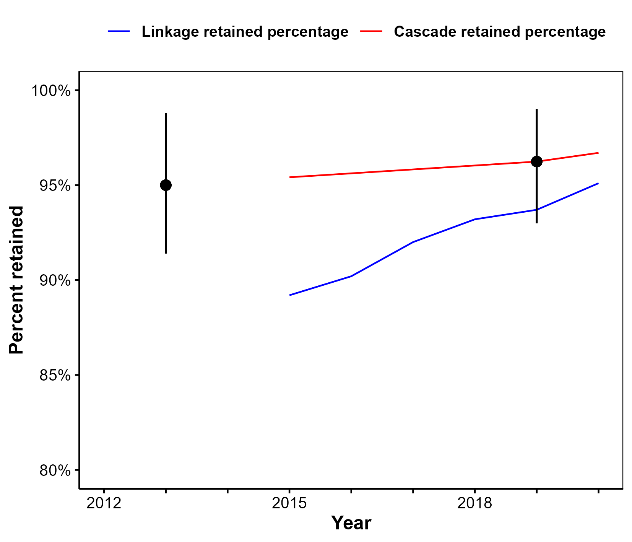 |

# Annual estimates for the Australian HIV cascade and related metrics

The following supplementary tables shows all the estimates and ranges for the HIV cascade steps the cascade gaps and other cascade related metrics for each year for the overall population, males, and females between 2004 and 2023.

## Overall population estimates

**Table S2:** **Annual estimates for each step of the HIV cascade for the overall population between 2004**–**2023**. Best estimates and the range calculated using the definitions and methods described in Section 1. Numerical values are rounded to the nearest 10.

| **Year** | **Living with HIV** | **Diagnosed with HIV** | **Retained in care** | **Receiving ART** | **Virally suppressed** |
| --- | --- | --- | --- | --- | --- |
| 2004 | 15,580  (14,410‒16,840) | 12,930  (11,890‒14,040) | NA | 7,600  (6,000‒9,300) | 4,570  (3,460‒5,810) |
| 2005 | 16,300  (14,990‒17,680) | 13,600  (12,430‒14,840) | NA | 8,450  (6,380‒9,830) | 5,590  (4,070‒6,730) |
| 2006 | 17,090  (15,630‒18,600) | 14,350  (13,030‒15,730) | NA | 9,460  (6,890‒10,460) | 6,930  (4,900‒7,880) |
| 2007 | 17,860  (16,250‒19,510) | 15,100  (13,620‒16,630) | NA | 9,930  (7,530‒11,180) | 7,600  (5,610‒8,780) |
| 2008 | 18,470  (16,720‒20,270) | 15,730  (14,090‒17,420) | NA | 10,200  (8,350‒12,010) | 8,190  (6,550‒9,870) |
| 2009 | 19,300  (17,390‒21,260) | 16,560  (14,770‒18,420) | NA | 10,900  (9,370‒12,940) | 8,970  (7,540‒10,890) |
| 2010 | 20,070  (17,990‒22,220) | 17,350  (15,390‒19,400) | NA | 12,400  (10,580‒13,970) | 10,560  (8,840‒12,130) |
| 2011 | 20,910  (18,640‒23,240) | 18,220  (16,090‒20,450) | NA | 13,620  (11,990‒15,100) | 11,770  (10,190‒13,280) |
| 2012 | 21,860  (19,410‒24,390) | 19,190  (16,890‒21,610) | NA | 14,980  (13,560‒16,310) | 13,380  (11,930‒14,790) |
| 2013 | 22,680  (20,030‒25,420) | 20,050  (17,560‒22,660) | 19,050  (16,050–22,390) | 16,510  (15,660‒17,370) | 15,250  (14,280‒16,250) |
| 2014 | 23,540  (20,710‒26,480) | 20,920  (18,250‒23,740) | 19,920  (17,850–23,460) | 17,900  (17,850‒18,210) | 16,720  (16,470‒17,210) |
| 2015 | 24,500  (21,500‒27,640) | 21,880  (19,310‒24,900) | 20,880  (19,310–24,620) | 19,380  (19,310‒19,700) | 18,360  (18,090‒18,870) |
| 2016 | 25,440  (22,240‒28,800) | 22,800  (20,360‒26,050) | 21,810  (20,360 –25,750) | 20,430  (20,360‒20,820) | 19,370  (19,090‒19,940) |
| 2017 | 26,280  (22,890‒29,880) | 23,640  (21,500‒27,110) | 22,660  (21,500–26,830) | 21,610  (21,500‒21,990) | 20,910  (20,630‒21,460) |
| 2018 | 27,040  (23,470‒30,870) | 24,410  (22,190‒28,100) | 23,450  (22,190–27,810) | 22,300  (22,190‒22,720) | 21,690  (21,420‒22,280) |
| 2019 | 27,920  (24,150‒31,980) | 25,300  (23,260‒29,210) | 24,350  (23,260–28,920) | 23,390  (23,260‒23,800) | 22,660  (22,340‒23,260) |
| 2020 | 28,640  (24,690‒32,890) | 26,000  (24,180‒30,040) | 25,140  (24,180–29,050) | 24,240  (24,180‒24,640) | 23,400  (23,100‒24,030) |
| 2021 | 29,060  (24,870‒33,550) | 26,350  (24,470‒30,560) | 25,480  (24,470–29,560) | 24,700  (24,470‒24,890) | 24,130  (23,690‒24,540) |
| 2022 | 29,480  (25,340‒34,310) | 26,820  (25,340‒31,290) | 25,940  (25,350–30,260) | 25,400  (25,340‒25,780) | 25,000  (24,760‒25,560) |
| 2023 | 30,010  (26,700‒35,220) | 27,650  (26,700‒32,390) | 26,740  (26,700–31,320) | 26,700  (26,700‒27,070) | 26,040  (25,780‒26,650) |

**Table S3:** **Annual estimates for the overall HIV cascade gaps and associated percentages between 2004–2023**. Best estimates presented with numerical values rounded to the nearest 10. The numbers and percentages calculated by taking the difference and quotient of the HIV cascade step estimates (presented in Figure S2) as described in the Methods section “Estimates of new HIV infections and other epidemic metrics” in the main text.

| **Year** | **Number undiagnosed** | **Number diagnosed but untreated** | **Number on ART but unsuppressed** | **Percentage of people with HIV diagnosed** | **Percentage diagnosed on ART** | **Percentage on ART suppressed** | **Percentage of people with HIV suppressed overall** |
| --- | --- | --- | --- | --- | --- | --- | --- |
| 2004 | 2,650 | 5,330 | 3,030 | 83% | 58·8% | 60·1% | 29·3% |
| 2005 | 2,700 | 5,150 | 2,860 | 83·5% | 62·1% | 66·1% | 34·3% |
| 2006 | 2,740 | 4,890 | 2,530 | 84% | 66% | 73·3% | 40·6% |
| 2007 | 2,760 | 5,160 | 2,330 | 84·5% | 65·8% | 76·5% | 42·6% |
| 2008 | 2,750 | 5,530 | 2,010 | 85·1% | 64·9% | 80·3% | 44·4% |
| 2009 | 2,740 | 5,660 | 1,930 | 85·8% | 65·8% | 82·3% | 46·5% |
| 2010 | 2,710 | 4,950 | 1,840 | 86·5% | 71·5% | 85·1% | 52·6% |
| 2011 | 2,680 | 4,610 | 1,840 | 87·2% | 74·7% | 86·5% | 56·3% |
| 2012 | 2,660 | 4,210 | 1,600 | 87·8% | 78·1% | 89·3% | 61·2% |
| 2013 | 2,630 | 3,540 | 1,260 | 88·4% | 82·4% | 92·4% | 67·2% |
| 2014 | 2,620 | 3,010 | 1,180 | 88·9% | 85·6% | 93·4% | 71% |
| 2015 | 2,620 | 2,500 | 1,020 | 89·3% | 88·6% | 94·7% | 74·9% |
| 2016 | 2,630 | 2,370 | 1,070 | 89·7% | 89·6% | 94·8% | 76·1% |
| 2017 | 2,640 | 2,040 | 700 | 90% | 91·4% | 96·8% | 79·6% |
| 2018 | 2,630 | 2,120 | 600 | 90·3% | 91·3% | 97·3% | 80·2% |
| 2019 | 2,620 | 1,910 | 730 | 90·6% | 92·5% | 96·9% | 81·2% |
| 2020 | 2,640 | 1,760 | 840 | 90·8% | 93·2% | 96·5% | 81·7% |
| 2021 | 2,720 | 1,640 | 570 | 90·7% | 93·8% | 97·7% | 83% |
| 2022 | 2,660 | 1,430 | 400 | 91% | 94·7% | 98·4% | 84·8% |
| 2023 | 2,360 | 950 | 670 | 92·1% | 96·6% | 97·5% | 86·8% |

**Table S4:** **Annual estimates for HIV epidemiological metrics for the overall population between 2004–2023**. Best estimates and the range presented. For the number of new infections, the range is given by the 95% confidence interval produced by the ECDC HIV Modelling Tool. The YDF, CDR, IPR, and IMR were calculated from the annual estimates of new infections and each step of the HIV cascade as described in the Methods section “Estimates of new HIV infections and other epidemic metrics” of the main text.

| **Year** | **Notifications excluding overseas diagnoses*** | **Notifications including overseas diagnoses*** | **Notifications previously diagnosed overseas*** | **Annual new infections** | **Yearly diagnosed fraction (YDF)** | **Case detection rate (CDR)** | **Incidence prevalence ratio (IPR)** | **Incidence mortality ratio (IMR)** |
| --- | --- | --- | --- | --- | --- | --- | --- | --- |
| 2004 | 894 | 918 | 24 | 910  (890‒930) | 0·25  (0·24‒0·26) | 0·95  (0·93‒0·98) | 6·1%  (5·6‒6·7%) | 7·93  (5·27‒12·1) |
| 2005 | 958 | 981 | 23 | 940  (920‒960) | 0·26  (0·25‒0·27) | 0·99  (0·97‒1·01) | 6%  (5·4‒6·6%) | 5·67  (4·07‒7·98) |
| 2006 | 985 | 1020 | 35 | 960  (940‒980) | 0·27  (0·26‒0·28) | 1·04  (1·02‒1·06) | 5·9%  (5·3‒6·5%) | 5·23  (3·84‒7·21) |
| 2007 | 950 | 1055 | 105 | 980  (960‒1,000) | 0·25  (0·25‒0·26) | 0·96  (0·95‒0·98) | 5·7%  (5·2‒6·4%) | 5·5  (4‒7·65) |
| 2008 | 902 | 1019 | 114 | 990  (970‒1,010) | 0·23  (0·22‒0·24) | 0·83  (0·81‒0·85) | 5·6%  (5‒6·2%) | 6·29  (4·44‒9·05) |
| 2009 | 947 | 1078 | 131 | 1,000  (980‒1,030) | 0·26  (0·25‒0·27) | 0·96  (0·94‒0·99) | 5·4%  (4·8‒6·1%) | 8·23  (5·5‒12·65) |
| 2010 | 914 | 1070 | 156 | 1,000  (970‒1,030) | 0·25  (0·25‒0·26) | 0·91  (0·89‒0·94) | 5·2%  (4·6‒5·9%) | 5·48  (4·03‒7·59) |
| 2011 | 983 | 1151 | 168 | 1,000  (970‒1,030) | 0·27  (0·26‒0·28) | 0·98  (0·95‒1·01) | 5%  (4·3‒5·7%) | 6·11  (4·38‒8·68) |
| 2012 | 1071 | 1264 | 193 | 990  (960‒1,020) | 0·29  (0·28‒0·3) | 1·12  (1·08‒1·16) | 4·7%  (4·1‒5·5%) | 8·11  (5·45‒12·41) |
| 2013 | 1036 | 1248 | 212 | 970  (940‒1,000) | 0·27  (0·26‒0·28) | 0·99  (0·96‒1·02) | 4·4%  (3·9‒5·2%) | 6·1  (4·33‒8·82) |
| 2014 | 1079 | 1338 | 259 | 940  (920‒980) | 0·31  (0·3‒0·32) | 1·23  (1·19‒1·26) | 4·2%  (3·6‒4·9%) | 4·16  (3·16‒5·6) |
| 2015 | 1029 | 1253 | 224 | 910  (890‒940) | 0·3  (0·29‒0·32) | 1·25  (1·21‒1·28) | 3·9%  (3·4‒4·6%) | 8·7  (5·42‒14·61) |
| 2016 | 1006 | 1266 | 260 | 880  (860‒900) | 0·3  (0·29‒0·31) | 1·29  (1·25‒1·32) | 3·6%  (3·1‒4·2%) | 5·14  (3·64‒7·52) |
| 2017 | 961 | 1252 | 291 | 830  (800‒860) | 0·29  (0·28‒0·3) | 1·3  (1·25‒1·35) | 3·3%  (2·8‒3·9%) | 3·46  (2·59‒4·72) |
| 2018 | 840 | 1132 | 292 | 780  (740‒820) | 0·26  (0·25‒0·27) | 1·21  (1·14‒1·28) | 3%  (2·5‒3·6%) | 3·93  (2·75‒5·77) |
| 2019 | 895 | 1240 | 345 | 720  (660‒770) | 0·27  (0·26‒0·28) | 1·38  (1·28‒1·5) | 2·6%  (2·1‒3·3%) | 3·69  (2·46‒5·65) |
| 2020 | 626 | 948 | 322 | 650  (570‒720) | 0·21  (0·19‒0·22) | 1·06  (0·95‒1·19) | 2·3%  (1·8‒3%) | 3·76  (2·27‒6·38) |
| 2021 | 541 | 734 | 193 | 570  (470‒670) | 0·18  (0·16‒0·19) | 1·02  (0·87‒1·22) | 2%  (1·4‒2·7%) | 1·94 (1·21‒3·14) |
| 2022 | 553 | 894 | 341 | 480  (360‒600) | 0·18  (0·16‒0·2) | 1·23  (0·98‒1·65) | 1·7%  (1·1‒2·4%) | 1·88  (0·96‒3·58) |
| 2023 | 722 | 1302 | 580 | 380  (230‒540) | 0·25  (0·22‒0·29) | 2·04  (1·46‒3·38) | 1·3%  (0·7‒2·1%) | 1·28  (0·54‒2·63) |

* Not adjusted for multiple reporting.

## Male estimates

**Table S5:** **Annual estimates for each step of the HIV cascade for males between 2004**–**2023**. Best estimates and the range calculated using the definitions and methods described in Section 1. Numerical values are rounded to the nearest 10.

| **Year** | **Living with HIV** | **Diagnosed with HIV** | **Retained in care** | **Receiving ART** | **Virally suppressed** |
| --- | --- | --- | --- | --- | --- |
| 2004 | 14,140  (13,110‒15,240) | 11,820  (10,920‒12,770) | NA | 6,560  (5,180‒8,030) | 3,970  (3,010‒5,060) |
| 2005 | 14,820  (13,680‒16,030) | 12,450  (11,440‒13,510) | NA | 7,300  (5,510‒8,490) | 4,860  (3,540‒5,860) |
| 2006 | 15,490  (14,240‒16,810) | 13,090  (11,960‒14,270) | NA | 8,170  (5,950‒9,030) | 6,020  (4,250‒6,850) |
| 2007 | 16,160  (14,790‒17,600) | 13,740  (12,480‒15,040) | NA | 8,580  (6,500‒9,650) | 6,580  (4,850‒7,610) |
| 2008 | 16,680  (15,180‒18,230) | 14,280  (12,880‒15,700) | NA | 8,810  (7,210‒10,370) | 7,090  (5,660‒8,550) |
| 2009 | 17,400  (15,750‒19,050) | 15,000  (13,480‒16,560) | NA | 9,410  (8,090‒11,170) | 7,790  (6,540‒9,450) |
| 2010 | 18,090  (16,290‒19,880) | 15,700  (14,040‒17,400) | NA | 10,710  (9,140‒12,060) | 9,130  (7,630‒10,490) |
| 2011 | 18,800  (16,860‒20,730) | 16,440  (14,630‒18,280) | NA | 11,760  (10,360‒13,040) | 10,240  (8,860‒11,560) |
| 2012 | 19,600  (17,510‒21,700) | 17,260  (15,310‒19,250) | NA | 12,940  (11,710‒14,080) | 11,610  (10,350‒12,830) |
| 2013 | 20,290  (18,040‒22,560) | 17,970  (15,880‒20,110) | 17,070  (14,510‒19,870) | 14,720  (13,930‒15,520) | 13,650  (12,740‒14,580) |
| 2014 | 21,020  (18,620‒23,450) | 18,700  (16,460‒21,000) | 17,810  (15,960‒20,760) | 16,000  (15,960‒16,260) | 14,950  (14,720‒15,380) |
| 2015 | 21,870  (19,330‒24,430) | 19,550  (17,210‒22,000) | 18,650  (17,210‒21,750) | 17,260  (17,210‒17,530) | 16,370  (16,130‒16,810) |
| 2016 | 22,630  (19,920‒25,400) | 20,300  (18,100‒22,960) | 19,410  (18,100‒22,710) | 18,160  (18,100‒18,480) | 17,220  (16,980‒17,730) |
| 2017 | 23,290  (20,350‒26,330) | 20,950  (19,050‒23,870) | 20,080  (19,050‒23,620) | 19,130  (19,050‒19,440) | 18,500  (18,250‒18,970) |
| 2018 | 23,860  (20,690‒27,180) | 21,520  (19,630‒24,720) | 20,670  (19,630‒24,470) | 19,700  (19,630‒20,050) | 19,180  (18,940‒19,680) |
| 2019 | 24,480  (21,030‒28,130) | 22,150  (20,480‒25,660) | 21,310  (20,480‒25,410) | 20,580  (20,480‒20,910) | 19,950  (19,680‒20,460) |
| 2020 | 24,980  (21,320‒28,870) | 22,630 (  21,170‒26,340) | 21,880  (21,170‒25,470) | 21,210  (21,170‒21,560) | 20,450  (20,190‒21,020) |
| 2021 | 25,230  (21,410‒29,450) | 22,790  (21,410‒26,760) | 22,040  (21,410‒25,880) | 21,580  (21,410‒21,750) | 21,110  (20,750‒21,470) |
| 2022 | 25,390  (22,070‒30,130) | 22,980  (22,070‒27,340) | 22,230  (22,070‒26,440) | 22,110  (22,070‒22,420) | 21,760  (21,550‒22,230) |
| 2023 | 25,650  (23,080‒30,950) | 23,490  (23,080‒28,240) | 23,080  (23,080‒27,310) | 23,080  (23,080‒23,380) | 22,520  (22,300‒23,050) |

**Table S6:** **Annual estimates for the male HIV cascade gaps and associated percentages between 2004–2023**. Best estimates presented with numerical values rounded to the nearest 10. The numbers and percentages calculated by taking the difference and quotient of the HIV cascade step estimates (presented in Figure S2) as described in the Methods section “Estimates of new HIV infections and other epidemic metrics” in the main text.

| **Year** | **Number undiagnosed** | **Number diagnosed but untreated** | **Number on ART but unsuppressed** | **Percentage of people with HIV diagnosed** | **Percentage diagnosed on ART** | **Percentage on ART suppressed** | **Percentage of people with HIV suppressed overall** |
| --- | --- | --- | --- | --- | --- | --- | --- |
| 2004 | 2,320 | 5,260 | 2,590 | 83·6% | 55·5% | 60·5% | 28·1% |
| 2005 | 2,360 | 5,160 | 2,440 | 84·1% | 58·6% | 66·6% | 32·8% |
| 2006 | 2,400 | 4,920 | 2,160 | 84·5% | 62·4% | 73·6% | 38·8% |
| 2007 | 2,420 | 5,160 | 2,000 | 85% | 62·4% | 76·7% | 40·7% |
| 2008 | 2,410 | 5,470 | 1,720 | 85·6% | 61·7% | 80·4% | 42·5% |
| 2009 | 2,400 | 5,590 | 1,630 | 86·2% | 62·7% | 82·7% | 44·7% |
| 2010 | 2,380 | 5,000 | 1,580 | 86·8% | 68·2% | 85·2% | 50·5% |
| 2011 | 2,360 | 4,680 | 1,510 | 87·4% | 71·5% | 87·1% | 54·5% |
| 2012 | 2,340 | 4,320 | 1,330 | 88·1% | 75% | 89·7% | 59·2% |
| 2013 | 2,320 | 3,250 | 1,080 | 88·6% | 81·9% | 92·7% | 67·3% |
| 2014 | 2,310 | 2,710 | 1,050 | 89% | 85·5% | 93·4% | 71·1% |
| 2015 | 2,320 | 2,290 | 890 | 89·4% | 88·3% | 94·8% | 74·8% |
| 2016 | 2,340 | 2,140 | 930 | 89·7% | 89·4% | 94·9% | 76·1% |
| 2017 | 2,340 | 1,820 | 640 | 89·9% | 91·3% | 96·7% | 79·4% |
| 2018 | 2,340 | 1,820 | 530 | 90·2% | 91·6% | 97·3% | 80·4% |
| 2019 | 2,330 | 1,570 | 630 | 90·5% | 92·9% | 97% | 81·5% |
| 2020 | 2,350 | 1,420 | 760 | 90·6% | 93·7% | 96·4% | 81·9% |
| 2021 | 2,440 | 1,220 | 470 | 90·3% | 94·7% | 97·8% | 83·7% |
| 2022 | 2,400 | 880 | 350 | 90·5% | 96·2% | 98·4% | 85·7% |
| 2023 | 2,160 | 410 | 550 | 91·6% | 98·2% | 97·6% | 87·8% |

**Table S7:** **Annual estimates for HIV epidemiological metrics for males between 2004–2023**. Best estimates and the range presented. For the number of new infections, the range is given by the 95% confidence interval produced by the ECDC HIV Modelling Tool. The YDF, CDR, IPR, and IMR were calculated from the annual estimates of new infections and each step of the HIV cascade as described in the Methods section “Estimates of new HIV infections and other epidemic metrics” of the main text.

| **Year** | **Notifications excluding overseas diagnoses*** | **Notifications including overseas diagnoses*** | **Notifications previously diagnosed overseas*** | **Annual new infections** | **Yearly diagnosed fraction (YDF)** | **Case detection rate (CDR)** | **Incidence prevalence ratio (IPR)** | **Incidence mortality ratio (IMR)** |
| --- | --- | --- | --- | --- | --- | --- | --- | --- |
| 2004 | 775 | 790 | 15 | 800  (780‒820) | 0·25  (0·23‒0·26) | 0·94  (0·91‒0·97) | 5·9%  (5·3‒6·6%) | 7·42  (4·87‒11·43) |
| 2005 | 864 | 883 | 19 | 830  (800‒850) | 0·27  (0·25‒0·28) | 1·03  (1·01‒1·06) | 5·9%  (5·3‒6·5%) | 5·36  (3·8‒7·6) |
| 2006 | 845 | 866 | 21 | 850  (830‒870) | 0·26  (0·25‒0·27) | 1·01  (0·98‒1·04) | 5·7%  (5·2‒6·4%) | 4·94  (3·57‒6·87) |
| 2007 | 843 | 915 | 72 | 870  (840‒890) | 0·26  (0·25‒0·27) | 0·98  (0·95‒1·01) | 5·6%  (5‒6·2%) | 5·21  (3·73‒7·32) |
| 2008 | 790 | 872 | 82 | 880  (850‒900) | 0·23  (0·22‒0·24) | 0·82  (0·8‒0·85) | 5·4%  (4·8‒6·1%) | 5·97  (4·14‒8·71) |
| 2009 | 827 | 929 | 102 | 890  (860‒910) | 0·26  (0·25‒0·27) | 0·95  (0·92‒0·98) | 5·3%  (4·7‒6.0%) | 7·84  (5·14‒12·17) |
| 2010 | 801 | 909 | 108 | 890  (860‒920) | 0·26  (0·25‒0·27) | 0·94  (0·91‒0·98) | 5·1%  (4·5‒5·8%) | 5·22  (3·79‒7·3) |
| 2011 | 881 | 1005 | 124 | 880  (850‒910) | 0·27  (0·26‒0·28) | 0·97  (0·94‒1·01) | 4·9%  (4·3‒5·6%) | 5·83  (4·14‒8·36) |
| 2012 | 965 | 1101 | 136 | 880  (840‒900) | 0·29  (0·28‒0·3) | 1·09  (1·06‒1·13) | 4·7%  (4·1‒5·4%) | 7·77  (5·16‒11·98) |
| 2013 | 927 | 1082 | 155 | 860  (830‒890) | 0·27  (0·26‒0·28) | 0·99 (0·96‒1·03) | 4·4%  (3·8‒5·1%) | 5·86  (4·11‒8·54) |
| 2014 | 974 | 1151 | 177 | 840  (810‒870) | 0·31  (0·3‒0·33) | 1·26  (1·21‒1·31) | 4·1%  (3·6‒4·8%) | 4·01  (2·99‒5·48) |
| 2015 | 919 | 1083 | 164 | 810  (780‒840) | 0·31  (0·3‒0·32) | 1·27  (1·22‒1·31) | 3·9%  (3·3‒4·5%) | 8·42  (5·15‒14·34) |
| 2016 | 913 | 1099 | 186 | 780  (760‒810) | 0·31  (0·3‒0·32) | 1·33  (1·28‒1·38) | 3·6%  (3·1‒4·2%) | 4·99  (3·46‒7·41) |
| 2017 | 845 | 1074 | 229 | 740  (710‒770) | 0·3  (0·29‒0·31) | 1·33  (1·28‒1·38) | 3·3%  (2·8‒3·9%) | 3·38  (2·51‒4·64) |
| 2018 | 755 | 978 | 223 | 700  (650‒740) | 0·27  (0·26‒0·28) | 1·25  (1·17‒1·33) | 3%  (2·5‒3·6%) | 3·87  (2·66‒5·76) |
| 2019 | 791 | 1060 | 269 | 640  (600‒710) | 0·28  (0·27‒0·29) | 1·39  (1·25‒1·49) | 2·7%  (2·2‒3·4%) | 3·66  (2·46‒5·76) |
| 2020 | 540 | 780 | 240 | 580  (510‒670) | 0·2  (0·19‒0·22) | 1·03  (0·89‒1·17) | 2·4%  (1·8‒3·2%) | 3·78  (2·28‒6·62) |
| 2021 | 475 | 610 | 135 | 520  (420‒630) | 0·18  (0·16‒0·19) | 1·0  (0·83‒1·24) | 2·1%  (1·5‒2·9%) | 1·98  (1·21‒3·28) |
| 2022 | 460 | 727 | 267 | 440  (310‒570) | 0·17  (0·15‒0·19) | 1·14  (0·88‒1·6) | 1·8%  (1·1‒2·7%) | 1·95  (0·96‒3·77) |
| 2023 | 619 | 1086 | 467 | 360  (200‒510) | 0·24  (0·2‒0·29) | 1·91  (1·34‒3·47) | 1·4%  (0·7‒2·3%) | 1·36  (0·54‒2·8) |

* Not adjusted for multiple reporting.

## Female estimates

**Table S8:** **Annual estimates for each step of the HIV cascade for females between 2004**–**2023**. Best estimates and the range calculated using the definitions and methods described in Section 1. Numerical values are rounded to the nearest 10.

| **Year** | **Living with HIV** | **Diagnosed with HIV** | **Retained in care** | **Receiving ART** | **Virally suppressed** |
| --- | --- | --- | --- | --- | --- |
| 2004 | 1,600  (1,460‒1,740) | 1,220  (1,130‒1,320) | NA | 920  (720‒1,120) | 500  (330‒720) |
| 2005 | 1,670  (1,530‒1,830) | 1,290  (1,190‒1,410) | NA | 1,020  (770‒1,190) | 590  (370‒810) |
| 2006 | 1,800  (1,650‒1,970) | 1,410  (1,300‒1,540) | NA | 1,140  (830‒1,260) | 780  (490‒970) |
| 2007 | 1,920  (1,750‒2,100) | 1,530  (1,390‒1,670) | NA | 1,200  (910‒1,350) | 880  (590‒1,100) |
| 2008 | 2,030  (1,840‒2,230) | 1,640  (1,490‒1,800) | NA | 1,230  (1,010‒1,450) | 970  (720‒1,250) |
| 2009 | 2,150  (1,940‒2,370) | 1,760  (1,590‒1,940) | NA | 1,320  (1,130‒1,560) | 1,020  (790‒1,330) |
| 2010 | 2,260  (2,030‒2,500) | 1,880  (1,690‒2,080) | NA | 1,500  (1,280‒1,690) | 1,250  (980‒1,520) |
| 2011 | 2,370  (2,120‒2,630) | 2,000  (1,790‒2,220) | NA | 1,640  (1,450‒1,820) | 1,290  (1,030‒1,550) |
| 2012 | 2,480  (2,210‒2,780) | 2,120  (1,890‒2,360) | NA | 1,810  (1,640‒1,970) | 1,530  (1,290‒1,780) |
| 2013 | 2,610  (2,320‒2,920) | 2,250  (2,000‒2,520) | 2,140  (1,830–2,490) | 1,790  (1,510‒2,070) | 1,590  (1,270‒1,940) |
| 2014 | 2,750  (2,430‒3,090) | 2,400  (2,120‒2,690) | 2,280  (1,950–2,660) | 1,900  (1,890‒1,950) | 1,770  (1,680‒1,880) |
| 2015 | 2,890  (2,550‒3,250) | 2,540  (2,240‒2,860) | 2,430  (2,100–2,820) | 2,120  (2,100‒2,170) | 1,980  (1,890‒2,120) |
| 2016 | 3,010  (2,660‒3,380) | 2,670  (2,360‒3,000) | 2,550  (2,260–2,970) | 2,270  (2,260‒2,330) | 2,140  (2,040‒2,280) |
| 2017 | 3,150  (2,800‒3,530) | 2,820  (2,500‒3,150) | 2,700  (2,440–3,120) | 2,470  (2,440‒2,540) | 2,410  (2,330‒2,540) |
| 2018 | 3,300  (2,940‒3,650) | 2,970  (2,660‒3,290) | 2,850  (2,560–3,260) | 2,590  (2,560‒2,660) | 2,510  (2,420‒2,650) |
| 2019 | 3,470  (3,120‒3,810) | 3,150  (2,850‒3,450) | 3,030  (2,770–3,410) | 2,810  (2,770‒2,880) | 2,700  (2,600‒2,860) |
| 2020 | 3,630  (3,290‒3,960) | 3,320  (3,030‒3,600) | 3,210  (3,010–3,480) | 3,020  (3,010‒3,080) | 2,960  (2,880‒3,090) |
| 2021 | 3,750  (3,400‒4,080) | 3,440  (3,160‒3,690) | 3,320  (3,070–3,570) | 3,130  (3,070‒3,140) | 3,040  (2,890‒3,150) |
| 2022 | 3,910  (3,570‒4,210) | 3,620  (3,370‒3,830) | 3,500  (3,280–3,710) | 3,290  (3,270‒3,360) | 3,240  (3,160‒3,380) |
| 2023 | 4,080  (3,750‒4,360) | 3,850  (3,630‒4,020) | 3,720  (3,620–3,890) | 3,620  (3,620‒3,690) | 3,510  (3,390‒3,680) |

**Table S9:** **Annual estimates for the female HIV cascade gaps and associated percentages between 2004–2022**. Best estimates presented with numerical values rounded to the nearest 10. The numbers and percentages calculated by taking the difference and quotient of the HIV cascade step estimates (presented in Figure S2) as described in the Methods section “Estimates of new HIV infections and other epidemic metrics” in the main text.

| **Year** | **Number undiagnosed** | **Number diagnosed but untreated** | **Number on ART but unsuppressed** | **Percentage of people with HIV diagnosed** | **Percentage diagnosed on ART** | **Percentage on ART suppressed** | **Percentage of people with HIV suppressed overall** |
| --- | --- | --- | --- | --- | --- | --- | --- |
| 2004 | 380 | 300 | 410 | 76·5% | 75·1% | 54·9% | 31·6% |
| 2005 | 380 | 270 | 430 | 77·4% | 78·8% | 58·2% | 35·5% |
| 2006 | 390 | 270 | 360 | 78·5% | 80·7% | 68·3% | 43·3% |
| 2007 | 390 | 330 | 320 | 79·7% | 78·4% | 73·1% | 45·7% |
| 2008 | 390 | 410 | 260 | 80·9% | 74·9% | 78·9% | 47·8% |
| 2009 | 390 | 450 | 300 | 82·1% | 74·6% | 77·6% | 47·5% |
| 2010 | 380 | 390 | 250 | 83·2% | 79·5% | 83·3% | 55·1% |
| 2011 | 370 | 350 | 360 | 84·3% | 82·3% | 78·4% | 54·4% |
| 2012 | 360 | 310 | 280 | 85·3% | 85·3% | 84·7% | 61·6% |
| 2013 | 360 | 460 | 200 | 86·3% | 79·4% | 88·8% | 60·9% |
| 2014 | 350 | 500 | 140 | 87·2% | 79·3% | 92·8% | 64·2% |
| 2015 | 350 | 420 | 140 | 88% | 83·4% | 93·6% | 68·7% |
| 2016 | 340 | 400 | 140 | 88·7% | 85·2% | 93·9% | 71% |
| 2017 | 330 | 350 | 50 | 89·4% | 87·5% | 97·8% | 76·4% |
| 2018 | 330 | 380 | 70 | 90·1% | 87·2% | 97·2% | 76·3% |
| 2019 | 320 | 340 | 100 | 90·7% | 89·1% | 96·3% | 77·9% |
| 2020 | 320 | 290 | 60 | 91·3% | 91·2% | 97·9% | 81·5% |
| 2021 | 310 | 310 | 80 | 91·6% | 91% | 97·3% | 81·2% |
| 2022 | 290 | 330 | 50 | 92·6% | 90·8% | 98·5% | 82·9% |
| 2023 | 230 | 230 | 120 | 94·3% | 94·1% | 96·7% | 85·8% |

**Table S10:** **Annual estimates for HIV epidemiological metrics for females between 2004–2022**. Best estimates and the range presented. For the number of new infections, the range is given by the 95% confidence interval produced by the ECDC HIV Modelling Tool. The YDF, CDR, IPR, and IMR were calculated from the annual estimates of new infections and each step of the HIV cascade as described in the Methods section “Estimates of new HIV infections and other epidemic metrics” of the main text.

| **Year** | **Notifications excluding overseas diagnoses*** | **Notifications including overseas diagnoses*** | **Notifications previously diagnosed overseas*** | **Annual new infections** | **Yearly diagnosed fraction (YDF)** | **Case detection rate (CDR)** | **Incidence prevalence ratio (IPR)** | **Incidence mortality ratio (IMR)** |
| --- | --- | --- | --- | --- | --- | --- | --- | --- |
| 2004 | 117 | 126 | 9 | 100  (100‒110) | 0·23  (0·21‒0·26) | 1·1  (1·03‒1·18) | 7%  (6·1‒8·1%) | 14·61  (9·31‒23·11) |
| 2005 | 93 | 97 | 4 | 100  (100‒110) | 0·19  (0·17‒0·21) | 0·83  (0·78‒0·89) | 6·6%  (5·7‒7·6%) | 9·85  (6·75‒14·52) |
| 2006 | 137 | 151 | 14 | 110  (100‒110) | 0·25  (0·23‒0·26) | 1·19  (1·1‒1·27) | 6·3%  (5·4‒7·4%) | 8·79  (6·14‒12·78) |
| 2007 | 106 | 139 | 33 | 110  (100‒110) | 0·21  (0·19‒0·22) | 0·95  (0·89‒1·01) | 5·9%  (5·1‒6·9%) | 8·72  (6·04‒12·88) |
| 2008 | 111 | 146 | 35 | 110  (100‒120) | 0·21  (0·19‒0·23) | 0·97  (0·89‒1·03) | 5·5%  (4·8‒6·6%) | 9·52  (6·4‒14·64) |
| 2009 | 117 | 146 | 29 | 100  (100‒120) | 0·24  (0·22‒0·26) | 1·13  (1·04‒1·21) | 5·2%  (4·4‒6·2%) | 11·83  (7·5‒19·45) |
| 2010 | 110 | 156 | 46 | 100  (100‒110) | 0·22  (0·2‒0·24) | 1  (0·9‒1·08) | 4·9%  (4·1‒5·9%) | 7·59  (5·3‒11·24) |
| 2011 | 101 | 145 | 44 | 100  (100‒110) | 0·23  (0·22‒0·26) | 1·11  (1·02‒1·18) | 4·5%  (3·8‒5·5%) | 8·16  (5·58‒12·28) |
| 2012 | 105 | 161 | 56 | 100  (90‒110) | 0·22  (0·2‒0·25) | 1·05  (0·97‒1·13) | 4·2%  (3·6‒5·1%) | 10·53 (6·73‒17·04) |
| 2013 | 106 | 162 | 56 | 100  (90‒110) | 0·23  (0·2‒0·25) | 1·07  (0·99‒1·15) | 3·9%  (3·3‒4·8%) | 7·73  (5·19‒11·81) |
| 2014 | 102 | 183 | 81 | 90  (90‒100) | 0·52  (0·48‒0·55) | 4·01  (3·73‒4·36) | 3·6%  (3‒4·4%) | 5·09  (3·61‒7·22) |
| 2015 | 108 | 168 | 50 | 90  (80‒100) | 0·52  (0·49‒0·56) | 4·25  (3·91‒4·7) | 3·3%  (2·6‒4.0%) | 10·25  (5·83‒18·36) |
| 2016 | 88 | 161 | 73 | 80  (80‒100) | 0·48  (0·45‒0·51) | 3·7  (3·29‒4·17) | 3.0%  (2·3‒3·8%) | 5·89  (3·72‒9·5) |
| 2017 | 107 | 167 | 60 | 80  (70‒90) | 0·5  (0·47‒0·54) | 4·22  (3·69‒4·88) | 2·7%  (2‒3·4%) | 3·86  (2·54‒5·87) |
| 2018 | 81 | 150 | 69 | 70  (60‒90) | 0·45  (0·43‒0·49) | 3·68  (3·17‒4·4) | 2·4%  (1·8‒3·1%) | 4·25  (2·54‒7·02) |
| 2019 | 94 | 170 | 76 | 70  (50‒80) | 0·46  (0·44‒0·51) | 4·11  (3·42‒5·16) | 2·1%  (1·5‒2·8%) | 3·84  (2·13‒6·8) |
| 2020 | 78 | 159 | 81 | 60  (40‒80) | 0·37  (0·34‒0·42) | 3·1  (2·45‒4·3) | 1·7%  (1·1‒2·5%) | 3·77  (1·74‒7·65) |
| 2021 | 61 | 115 | 54 | 50  (30‒70) | 0·3  (0·26‒0·36) | 2·53  (1·81‒4·14) | 1·5%  (0·8‒2·2%) | 1·87  (0·8‒3·82) |
| 2022 | 85 | 156 | 71 | 40  (20‒70) | 0·36  (0·3‒0·45) | 3·65  (2·28‒8·1) | 1·2%  (0·5‒2·1%) | 1·75  (0·5‒4·56) |
| 2023 | 96 | 206 | 110 | 40  (10‒70) | 0·48  (0·39‒0·65) | 6·12  (3·19‒31·37) | 0·9%  (0·2‒1·9%) | 1·14  (0·14‒3·54) |

* Not adjusted for multiple reporting.

# Statistical analysis of trends

This section provides additional details of the trend analysis for each step of the HIV cascade and associated metrics estimated in the study. The file ~/HIV/4-HivCascadeTrends.Rmd within the online code repository contains all the code used to conduct the exploration of trends and the final trend analysis.^1^

To assess annual trends for each HIV metric we fitted continuous piecewise negative binomial regression models to the best estimates with time (year) as an independent covariate. These models were used to produce estimates for the average annual rate of change (given by the annual rate ratio or ARR) and the time when a change in trend occurred (called a ‘change point’). For metrics which are a proportion (or percentage) we used the denominator as an offset to ensure the fitted estimate remained below one. Starting from the overall (zero change point) model, we produced regression models with one, two, three, and four change points in the best estimates using the iterative algorithm contained in the R package ‘segmented’ (version 2.0-2).^24,25^ The algorithm does not assume that change points exists or that they occur at a specific time. We assumed a maximum of five change points given there were only 20 years of estimates and to ensure a minimum gap of four years between change points.

The algorithm in the segmented package requires an initial estimate for the location of the specified number of change points. We assumed the initial change point estimates were distributed equally between 2004–2023 when running the algorithm. We found varying the initial values had no impact on the final location of any detected change points. If the algorithm failed to find the specified number of change points (producing an error) we replaced the corresponding segmented model with the overall regression model for analysis purposes. The final model was selected by choosing the one with the minimum Bayesian information criterion (BIC) excluding models with change points within four years of each other or within four years after 2004. We allowed change points within four years of 2023 given the potential impact of the COVID-19 pandemic.

## Analysis results for males

This section provides tables and figures of results for males corresponding to those for the overall population presented in the main text.

**Table S11: Summary results for each cascade HIV step and metric for males.** Cascade step, cascade gap and new HIV infection estimates rounded to nearest 10.

| **Metric** | **2004 best estimate**  **(range)** | **2023 best estimate**  **(range)** | **Change point**  **Estimates (95% CI)** | **ARR between change points**  **(95% CI)** |
| --- | --- | --- | --- | --- |
| **HIV cascade steps** |  |  |  |  |
| Number of people living with HIV | 14,140  (13,110‒15,240) | 25,650  (23,080‒30,950) | 2015·4  (2015·2‒2015·6) | 1·04  (1·04‒1·04) |
|  |  |  | 2019·7  (2019·5‒2019·8) | 1·026  (1·025‒1·027) |
|  |  |  |  | 1·009  (1·008‒1·009) |
| Diagnosed | 11,820  (10,920‒12,770) | 23,490  (23,080‒28,240) | 2014·6  (2014·4‒2014·8) | 1·047  (1·047‒1·047) |
|  |  |  | 2018·8  (2018·7‒2019) | 1·033  (1·032‒1·033) |
|  |  |  |  | 1·013  (1·013‒1·014) |
| Receiving ART | 6,560  (5,180‒8,030) | 23,080  (23,080‒23,380) | 2009  (2008·6‒2009·4) | 1·073  (1·07‒1·076) |
|  |  |  | 2015·3  (2015·2‒2015·5) | 1·105  (1·103‒1·106) |
|  |  |  |  | 1·032  (1·031‒1·033) |
| Virally suppressed | 3,970  (3,010‒5,060) | 22,520  (22,300‒23,050) | 2014·8  (2014·6‒2014·9) | 1·134  (1·132‒1·136) |
|  |  |  |  | 1·039  (1·037‒1·041) |
| **HIV cascade gaps** |  |  |  |  |
| Number with undiagnosed HIV | 2,320 | 2,160 | 2019·6  (2018·6‒2020·7) | 0·998  (0·997‒1) |
|  |  |  | 2021·7  (2021·3‒2022·1) | 1·035  (1·002‒1·069) |
|  |  |  |  | 0·9  (0·871‒0·93) |
| Number diagnosed with HIV but untreated | 5,260 | 410 | 2010·6  (2010·5‒2010·7) | 1·004  (1·002‒1·007) |
|  |  |  | 2014·6  (2014·4‒2014·9) | 0·824  (0·819‒0·83) |
|  |  |  | 2021·6  (2021·6‒2021·7) | 0·902  (0·9‒0·905) |
|  |  |  |  | 0·47  (0·456‒0·485) |
| Number on ART but with an unsuppressed viral load | 2,590 | 550 | NA | 0·909  (0·9‒0·918) |
| Percentage of people living with HIV diagnosed | 83·6% | 91·6% | 2014·5  (2013·4‒2015·6) | 1·007  (1·006‒1·007) |
|  |  |  |  | 1·002  (1·002‒1·003) |
| Percentage diagnosed with HIV on ART | 55·5% | 98·2% | 2009  (2008·7‒2009·4) | 1·022  (1·02‒1·024) |
|  |  |  | 2014·4  (2014·2‒2014·6) | 1·061  (1·058‒1·063) |
|  |  |  |  | 1·012  (1·011‒1·013) |
| Percentage on ART virally suppressed | 60·5% | 97·6% | 2009·5  (2009·2‒2009·8) | 1·064  (1·059‒1·068) |
|  |  |  |  | 1·01  (1·009‒1·011) |
| Percentage of people living with HIV virally suppressed overall | 28·1% | 87·8% | 2014·5  (2014·2‒2014·7) | 1·09  (1·089‒1·092) |
|  |  |  |  | 1·019  (1·017‒1·021) |
| **HIV cascade related metrics** |  |  |  |  |
| Notifications excluding previously diagnosed overseas | 780 | 620 | 2015·3  (2014·1‒2016·4) | 1·016  (1·006‒1·026) |
|  |  |  |  | 0·917  (0·901‒0·934) |
| Notifications including previously diagnosed overseas | 790 | 1,090 | 2010·9  (2009·5‒2012·4) | 1·036  (1·029‒1·043) |
|  |  |  | 2018·8  (2018·6‒2019) | 1·008  (1·003‒1·013) |
|  |  |  | 2021  (2020·8‒2021·2) | 0·753  (0·717‒0·79) |
|  |  |  |  | 1·334  (1·301‒1·368) |
| Notifications of HIV previously diagnosed overseas | 20 | 470 | 2008·3  (2007·5‒2009) | 1·622  (1·463‒1·797) |
|  |  |  |  | 1·085  (1·069‒1·101) |
| Annual new infections (range = 95% CI) | 800  (780‒820) | 360  (200‒510) | 2012·4  (2012·2‒2012·6) | 1·011  (1·01‒1·012) |
|  |  |  | 2019·5  (2019·3‒2019·6) | 0·953  (0·951‒0·955) |
|  |  |  |  | 0·852  (0·847‒0·858) |
| Yearly diagnosed fraction (YDF) | 0·25  (0·23‒0·26) | 0·24  (0·2‒0·29) | 2016  (2014·3‒2017·7) | 1·018  (1·008‒1·029) |
|  |  |  |  | 0·935  (0·911‒0·96) |
| Case detection rate (CDR) | 0·94  (0·91‒0·97) | 1·91  (1·34‒3·47) | 2008  (2007·1‒2009) | 0·968  (0·948‒0·988) |
|  |  |  | 2018·7  (2018·2‒2019·1) | 1·044  (1·037‒1·051) |
|  |  |  | 2021·5  (2021·3‒2021·7) | 0·848  (0·809‒0·89) |
|  |  |  |  | 1·678  (1·522‒1·849) |
| Incidence prevalence ratio (IPR; %) | 5·9% (5·3‒6·6%) | 1·4% (0·7‒2·3%) | 2012·4 (2012·2‒2012·6) | 0·97  (0·969‒0·972) |
|  | 0% (0‒0%) | 0% (0‒0%) | 2019·5 (2019·3‒2019·7) | 0·922  (0·92‒0·924) |
|  | 0% (0‒0%) | 0% (0‒0%) | 0 (0‒0) | 0·842  (0·837‒0·847) |
| Incidence mortality ratio (IMR) | 7·42  (4·87‒11·43) | 1·36  (0·54‒2·8) | 2015  (2013·3‒2016·7) | 1·007  (0·983‒1·031) |
|  |  |  |  | 0·843  (0·807‒0·881) |

Acronyms: ARR = Annual Rate Ratio; CI = Confidence Interval.

The ARR shows the annual increase (value > 1) or decrease (value < 1) of the value of a metric (if the metric is constant then the ARR = 1). Note results that are generated using statistical methods have uncertainties specified by the resulting 95% confidence interval. This includes the annual number of new infections which were obtained using bootstrapping within the ECDC HIV Modelling Tool. Uncertainties for each metric are called ranges because they are generated using a combination of data and calculations.


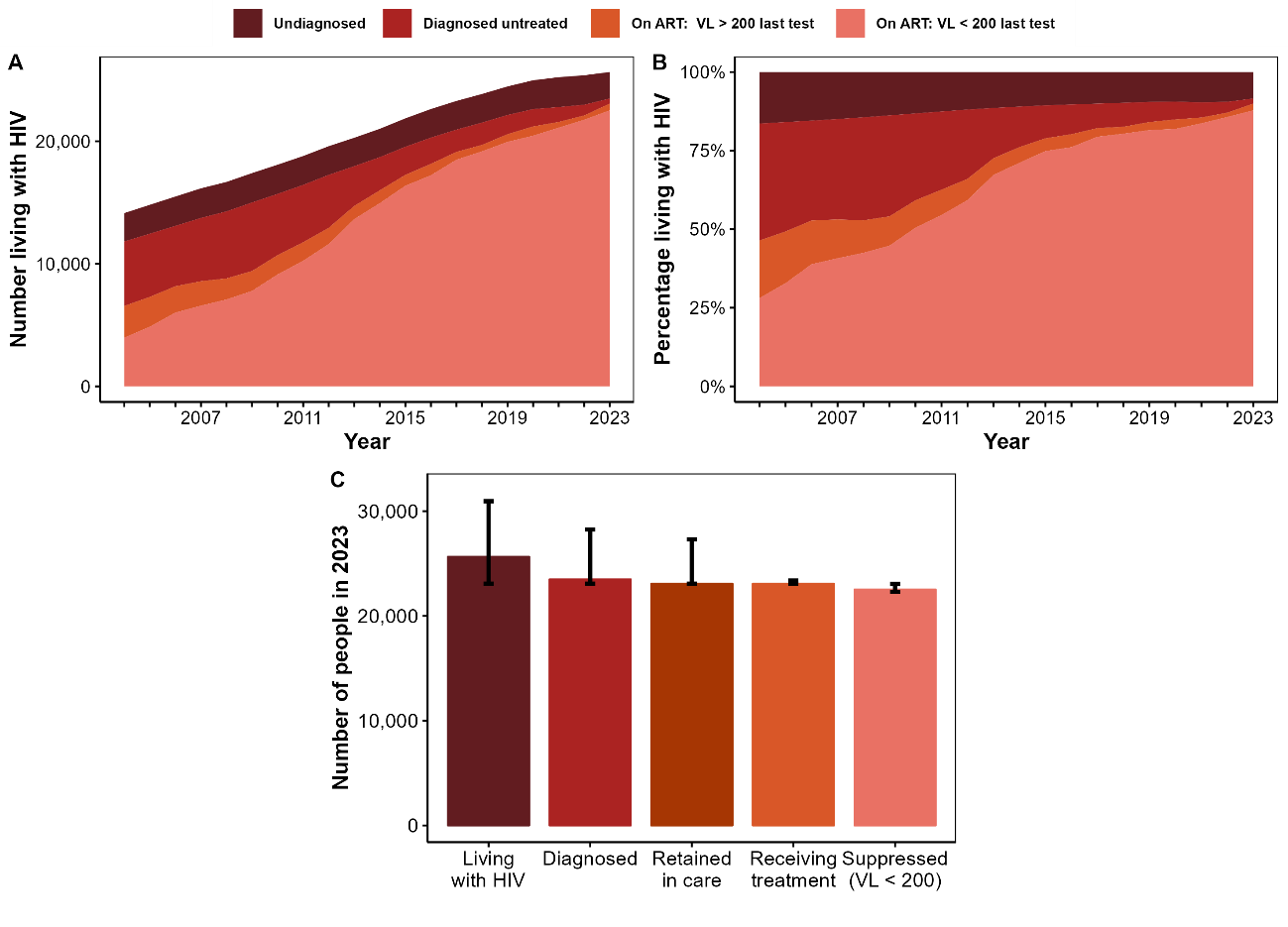


**Figure S6:** **HIV cascade to the end of 2023** **for males**. A) Estimated numbers and B) percentages of people living with HIV in each step of the HIV cascade over 2004–2023. C) HIV cascade best estimates and uncertainty ranges at the end of 2023 including retained in care (note lower range of Diagnosed and Retained in care adjusted to equal lower range of number Receiving treatment). The annual estimates and uncertainty ranges for each step of the male HIV cascade are provided in Table S5.


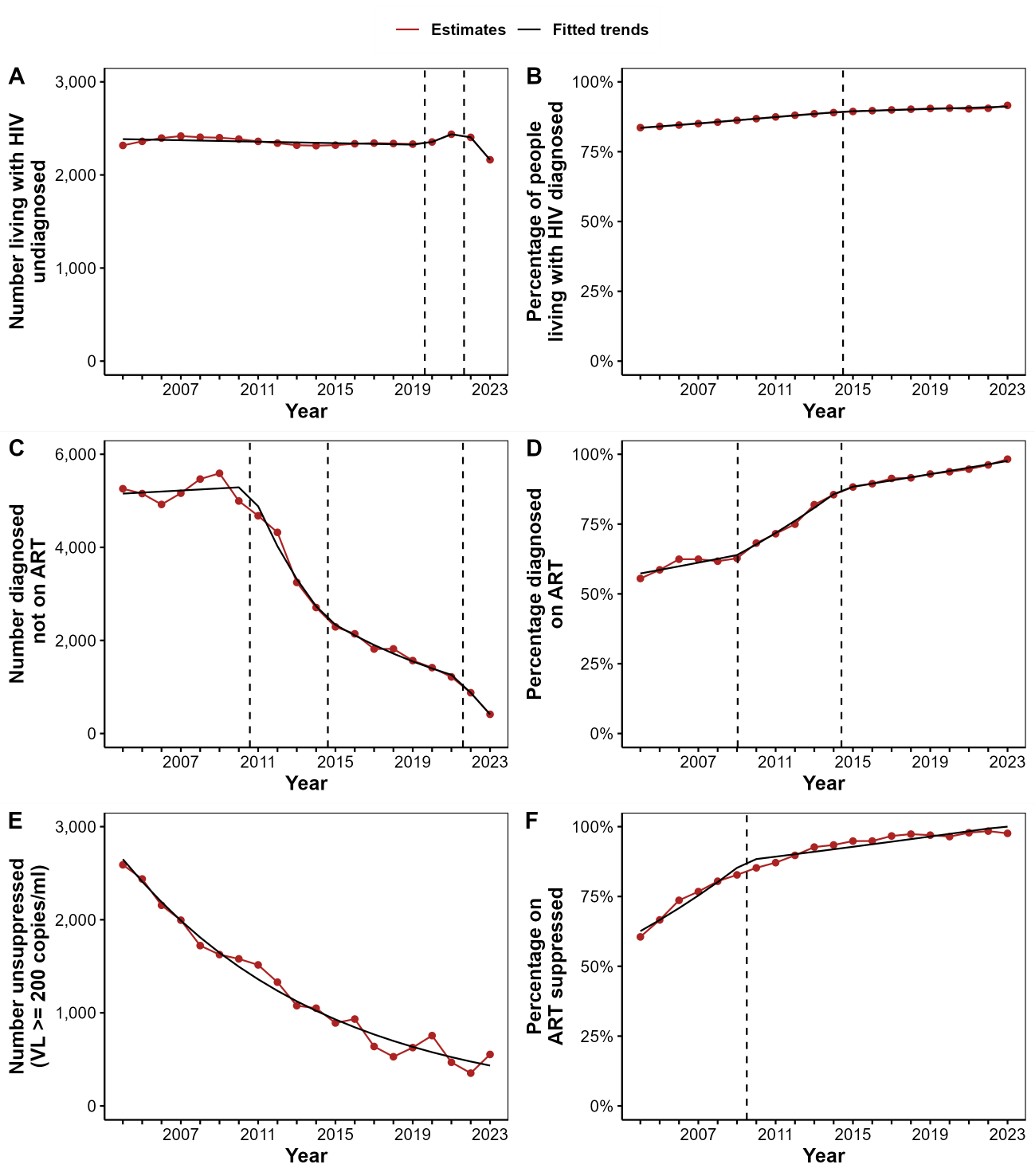


**Figure S7: Estimates and trends for the number (A, C, E) and percentage (B, D, F) of people living with HIV in the gaps of the male HIV cascade between 2004–2023.** Red lines show the best estimated values from the HIV cascade calculations. Black lines show the best fitting model predictions. Vertical dashed lines show the location of detected change points. No uncertainty ranges were produced for these metrics due to them being calculated from the cascade steps which are interdependent. The estimates for each gap estimate and percentage for the male HIV cascade are provided in Table S6.


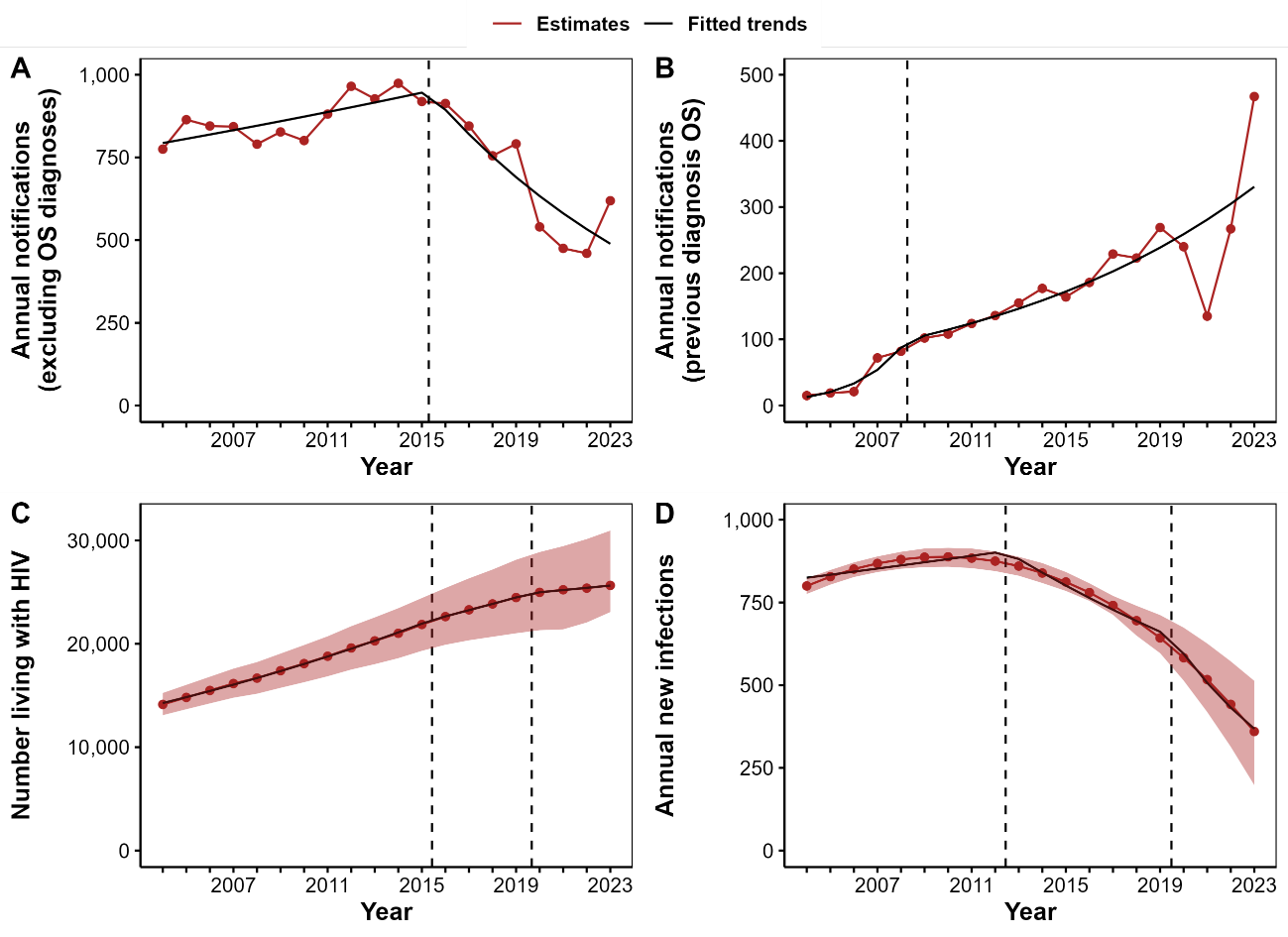


**Figure S8: Trends in notifications (A and B), number of people living with HIV (C) and annual new infections (D) between 2004-2023 for males.** Red lines and shading show the best estimated values and ranges from the HIV cascade calculations. Note there is no red shading for annual notifications data as they are directly from the national HIV registry. Black lines show the best fitting piecewise negative binomial model predictions. Vertical dashed lines show the location of detected change points. (OS = overseas). The estimates for males are provided in Table S7.


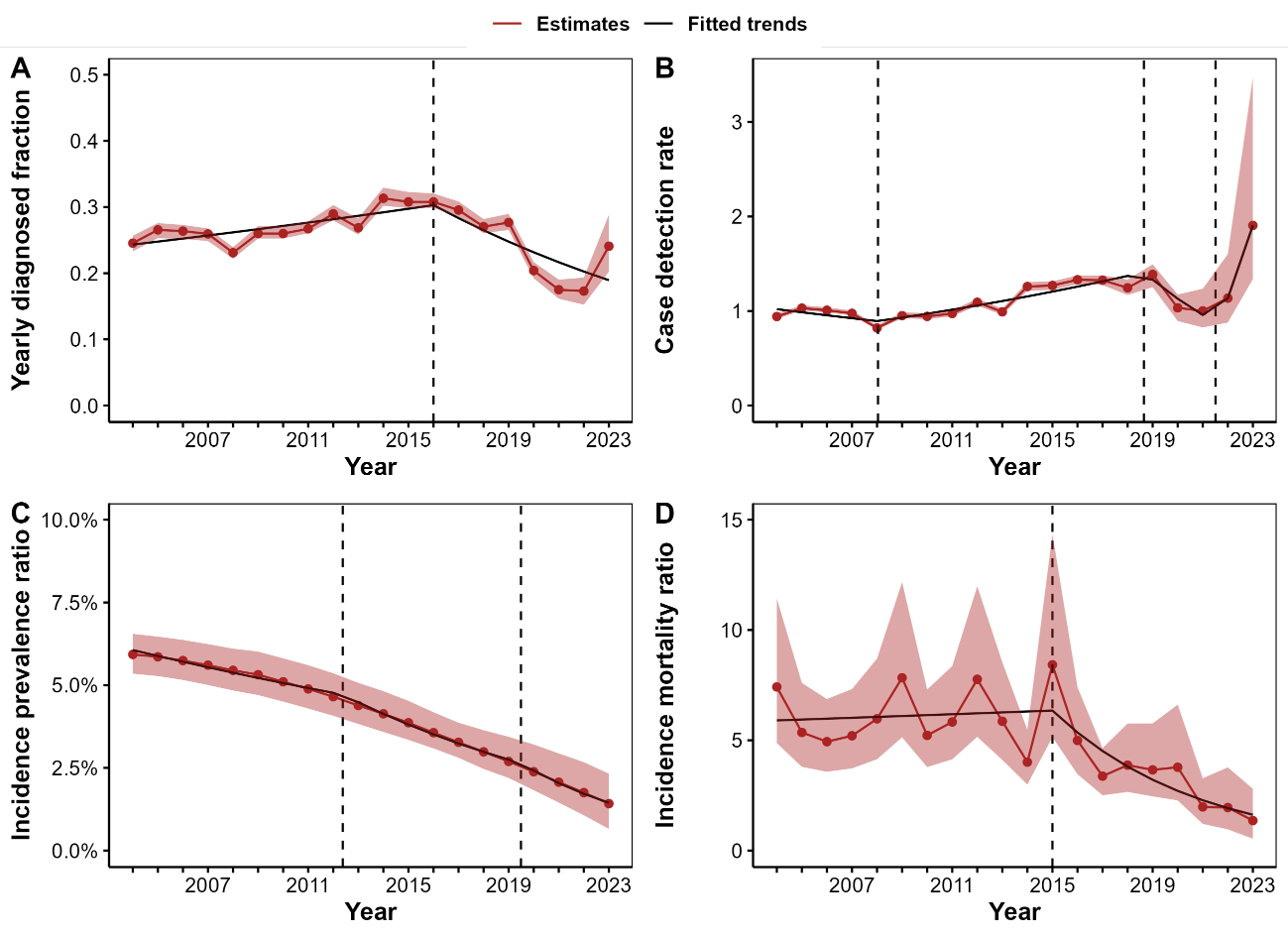


**Figure S9: Trends in other cascade related metrics between 2004-2023 for males.** A) YDF, B) CDR, C) IPR, and D) IMR. Red lines and shading show the best estimated values and ranges from the HIV cascade calculations. Black lines show the best fitting piecewise negative binomial model predictions. Vertical dashed lines show the location of detected change points. The estimates for males are provided in Table S7.

## Analysis results for females

This section provides tables and figures of results for males corresponding to those for the overall population presented in the main text.

**Table S12: Summary results for each cascade HIV step and metric for females.** Cascade step, cascade gap and new HIV infection estimates rounded to nearest 10.

| **Metric** | **2004 best estimate**  **(range)** | **2023 best estimate**  **(range)** | **Change point**  **Estimates (95% CI)** | **ARR between change points**  **(95% CI)** |
| --- | --- | --- | --- | --- |
| **HIV cascade steps** |  |  |  |  |
| Number of people living with HIV | 1,600  (1,460‒1,740) | 4,080  (3,750‒4,360) | 2010·3  (2009·2‒2011·5) | 1·061  (1·058‒1·065) |
|  |  |  |  | 1·046  (1·045‒1·047) |
| Diagnosed | 1,220  (1,130‒1,320) | 3,850  (3,630‒4,020) | 2011·5  (2010·7‒2012·4) | 1·074  (1·071‒1·078) |
|  |  |  |  | 1·054  (1·053‒1·056) |
| Receiving ART | 920  (720‒1,120) | 3,620  (3,620‒3,690) | NA | 1·073  (1·07‒1·075) |
| Virally suppressed | 500  (330‒720) | 3,510  (3,390‒3,680) | 2010·3  (2009·4‒2011·1) | 1·153  (1·141‒1·166) |
|  |  |  |  | 1·084  (1·08‒1·088) |
| **HIV cascade gaps** |  |  |  |  |
| Number with undiagnosed HIV | 380 | 230 | 2021·6  (2021·1‒2022) | 0·987  (0·985‒0·989) |
|  |  |  |  | 0·806  (0·741‒0·877) |
| Number diagnosed with HIV but untreated | 300 | 230 | 2014  (2012·5‒2015·5) | 1·046  (1·028‒1·064) |
|  |  |  |  | 0·942  (0·925‒0·959) |
| Number on ART but with an unsuppressed viral load | 410 | 120 | 2012  (2010·1‒2013·9) | 0·951  (0·906‒0·999) |
|  |  |  | 2017·1  (2015·5‒2018·8) | 0·781  (0·72‒0·847) |
|  |  |  |  | 1·026  (0·942‒1·117) |
| Percentage of people living with HIV diagnosed | 76·5% | 94·3% (0‒0%) | NA | 1·01  (1·009‒1·012) |
| Percentage diagnosed with HIV on ART | 75·1% | 94·1% | NA | 1·012  (1·01‒1·014) |
| Percentage on ART virally suppressed | 54·9% | 96·7% | NA | 1·026  (1·02‒1·031) |
| Percentage of people living with HIV virally suppressed overall | 31·6% | 85·8% | 2010·3  (2009·2‒2011·5) | 1·086  (1·074‒1·099) |
|  |  |  |  | 1·036  (1·032‒1·039) |
| **HIV cascade related metrics** |  |  |  |  |
| Notifications excluding previously diagnosed overseas | 120 | 100 | NA | 0·98  (0·972‒0·989) |
| Notifications including previously diagnosed overseas | 130 | 210 | 2014·4  (2012·7‒2016·1) | 1·038  (1·025‒1·051) |
|  |  |  | 2021·5  (2020·8‒2022·3) | 0·963  (0·94‒0·986) |
|  |  |  |  | 1·321  (1·12‒1·558) |
| Notifications of HIV previously diagnosed overseas | 10 | 110 | NA | 1·099  (1·067‒1·132) |
| Annual new infections (range = 95% CI) | 100  (100‒110) | 40  (10‒70) | 2015·7  (2015‒2016·4) | 0·988  (0·982‒0·994) |
|  |  |  |  | 0·889  (0·877‒0·901) |
| Yearly diagnosed fraction (YDF) | 0·23  (0·21‒0·26) | 0·48  (0·39‒0·65) | 2016·9  (2013·4‒2020·3) | 1·078  (1·047‒1·11) |
|  |  |  |  | 0·968  (0·9‒1·041) |
| Case detection rate (CDR) | 1·1  (1·03‒1·18) | 6·12  (3·19‒31·37) | NA | 1·107  (1·076‒1·138) |
| Incidence prevalence ratio (IPR; %) | 7%  (6·1‒8·1%) | 0·9%  (0·2‒1·9%) | 2016·6  (2015·9‒2017·3) | 0·932  (0·928‒0·936) |
|  |  |  |  | 0·842  (0·828‒0·855) |
| Incidence mortality ratio (IMR) | 14·61  (9·31‒23·11) | 1·14  (0·14‒3·54) | 2015  (2013·2‒2016·8) | 0·963  (0·939‒0·986) |
|  |  |  |  | 0·813  (0·775‒0·853) |

Acronyms: ARR = Annual Rate Ratio; CI = Confidence Interval.

The ARR shows the annual increase (value > 1) or decrease (value < 1) of the value of a metric (if the metric is constant then the ARR = 1). Note results that are generated using statistical methods have uncertainties specified by the resulting 95% confidence interval. This includes the annual number of new infections which were obtained using bootstrapping within the ECDC HIV Modelling Tool. Uncertainties for each metric are called ranges because they are generated using a combination of data and calculations.


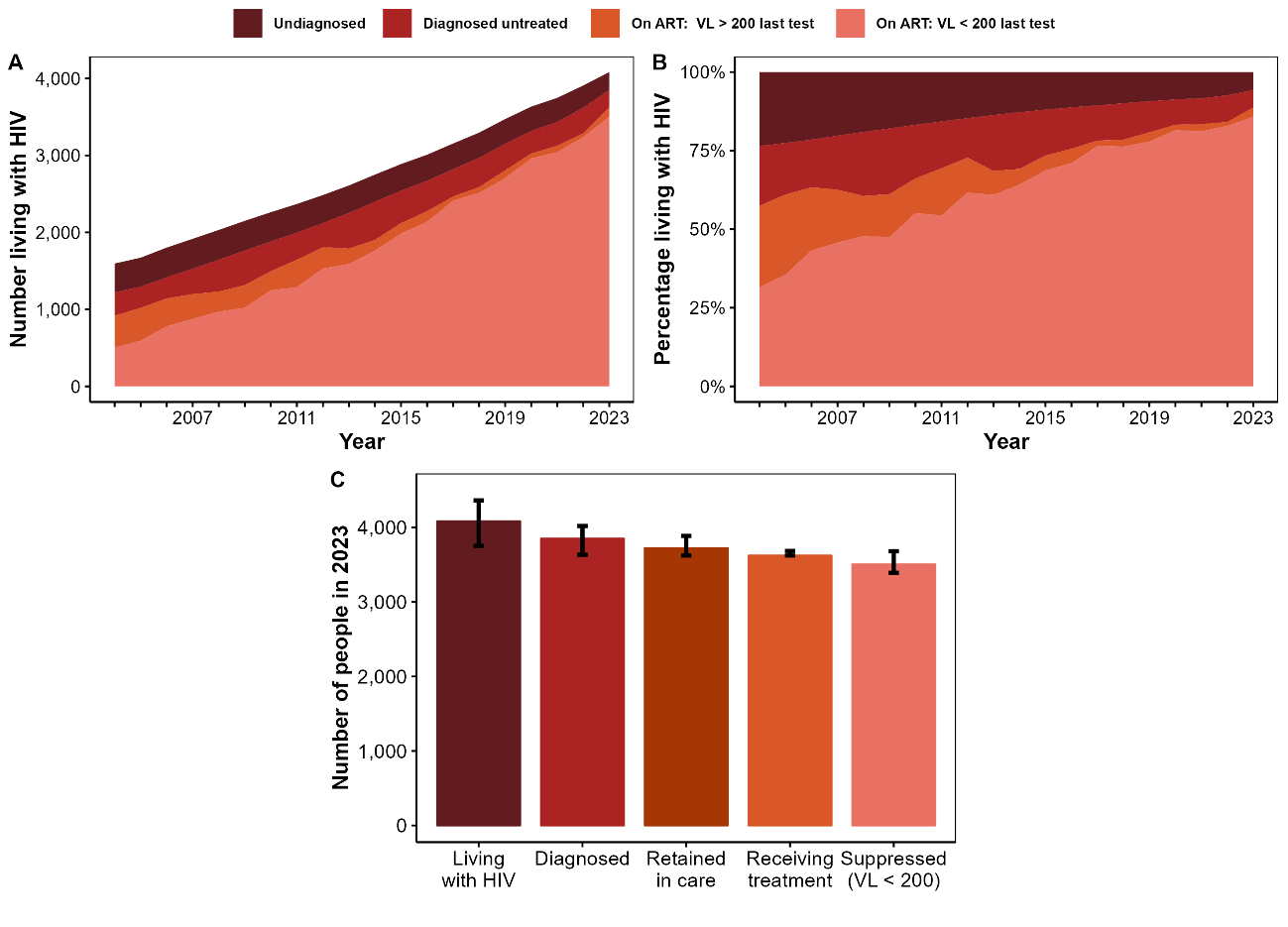


**Figure S10:** **HIV cascade to the end of 2023** **for females**. A) Estimated numbers and B) percentages of people living with HIV in each step of the HIV cascade over 2004–2023. C) HIV cascade best estimates and uncertainty ranges at the end of 2023 including retained in care (note lower range of Diagnosed and Retained in care adjusted to equal lower range of number Receiving treatment). The annual estimates and uncertainty ranges for each step of the female HIV cascade are provided in Table S8.


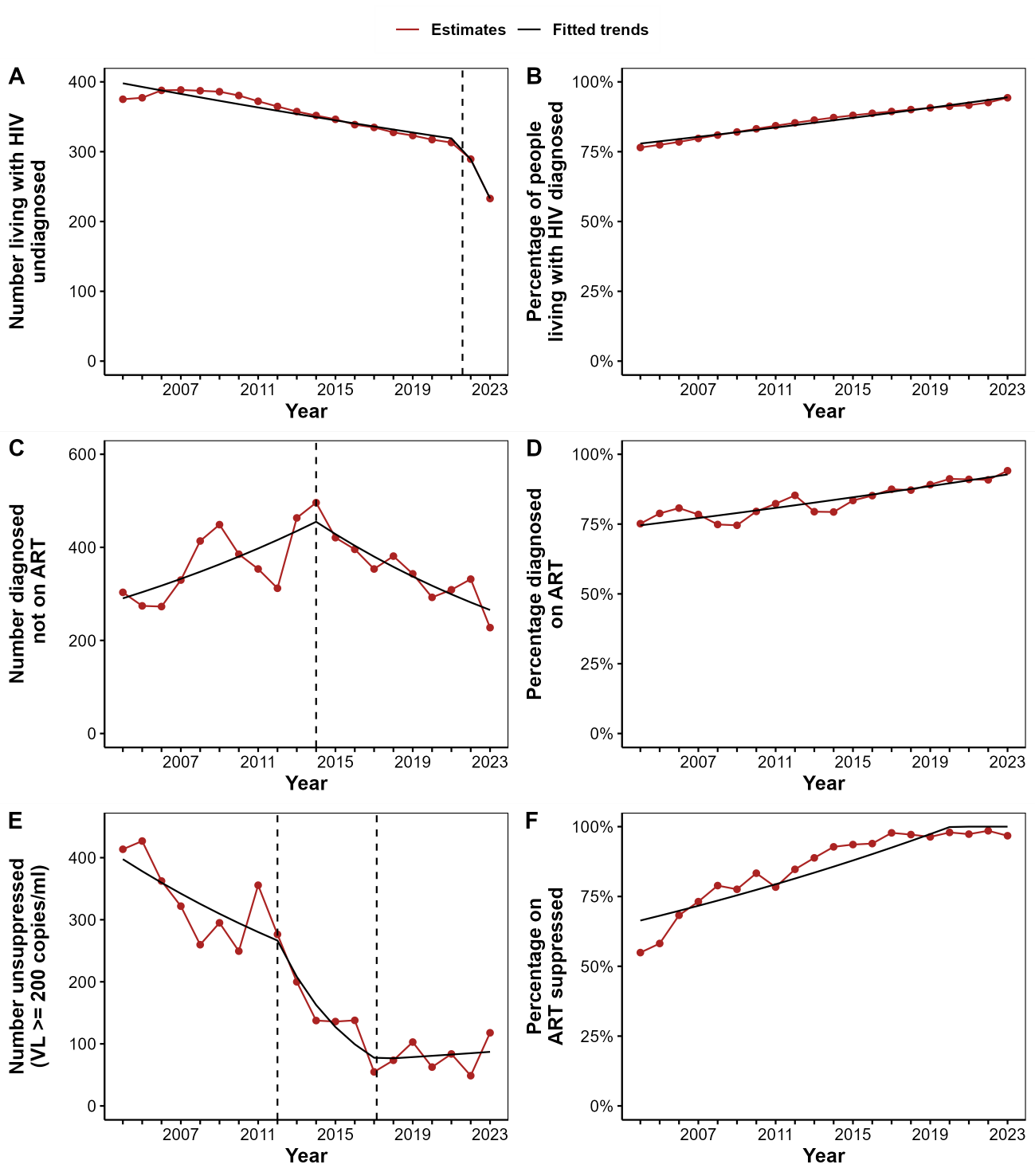


**Figure S11: Estimates and trends for the number (A, C, E) and percentage (B, D, F) of people living with HIV in the gaps of the female HIV cascade between 2004–2023.** Red lines show the best estimated values from the HIV cascade calculations. Black lines show the best fitting model predictions. Vertical dashed lines show the location of detected change points. No uncertainty ranges were produced for these metrics due to them being calculated from the cascade steps which are interdependent. The estimates for each gap estimate and percentage for the female HIV cascade are provided in Table S9.


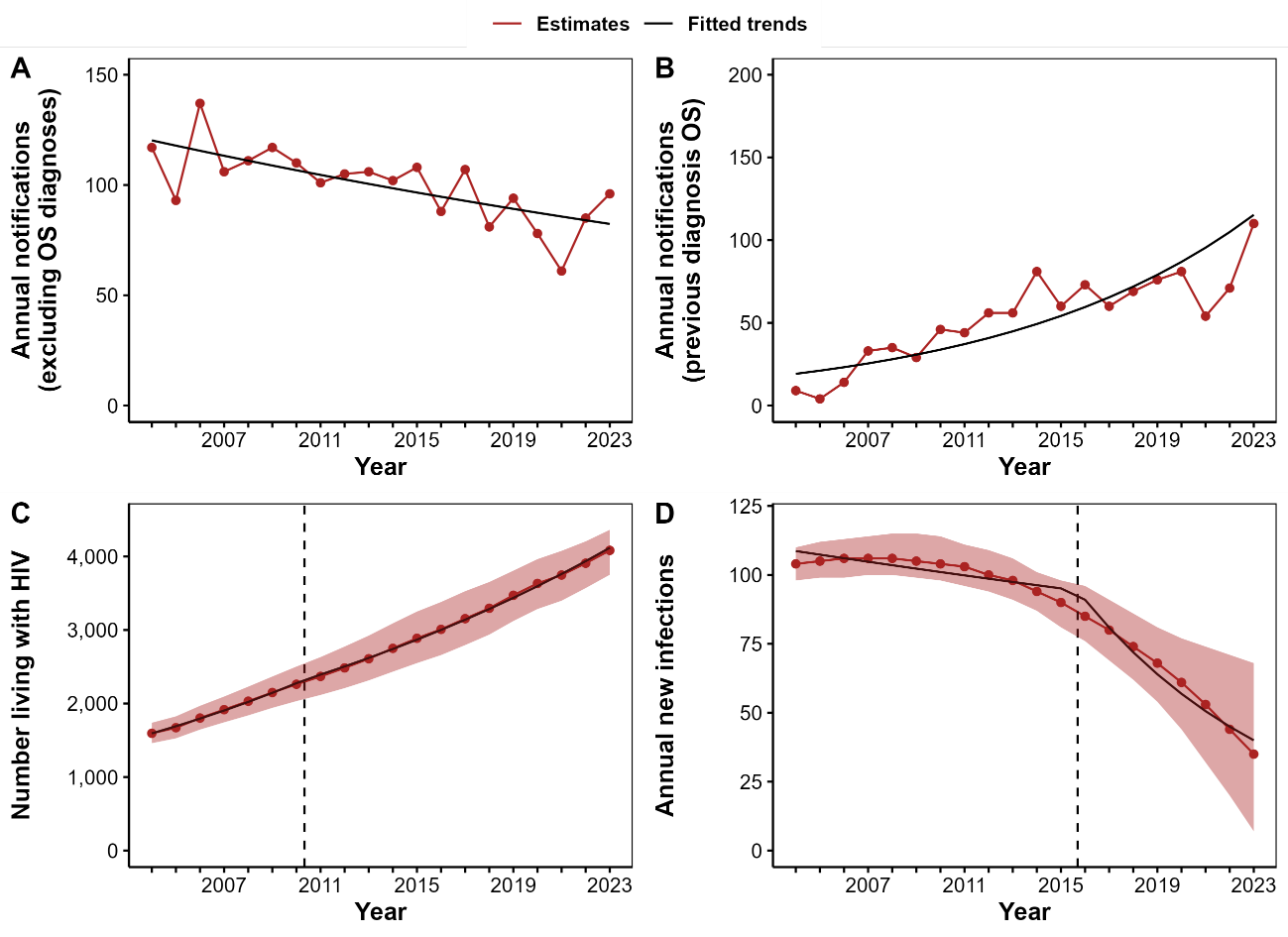


**Figure S12: Trends in notifications (A and B), number of people living with HIV (C), and annual new infections (D) between 2004-2023 for females.** Red lines and shading show the best estimated values and ranges from the HIV cascade calculations. Note there is no red shading for annual notifications data as they are directly from the national HIV registry. Black lines show the best fitting piecewise negative binomial model predictions. Vertical dashed lines show the location of detected change points. (OS = overseas). The estimates for females are provided in Table S10.


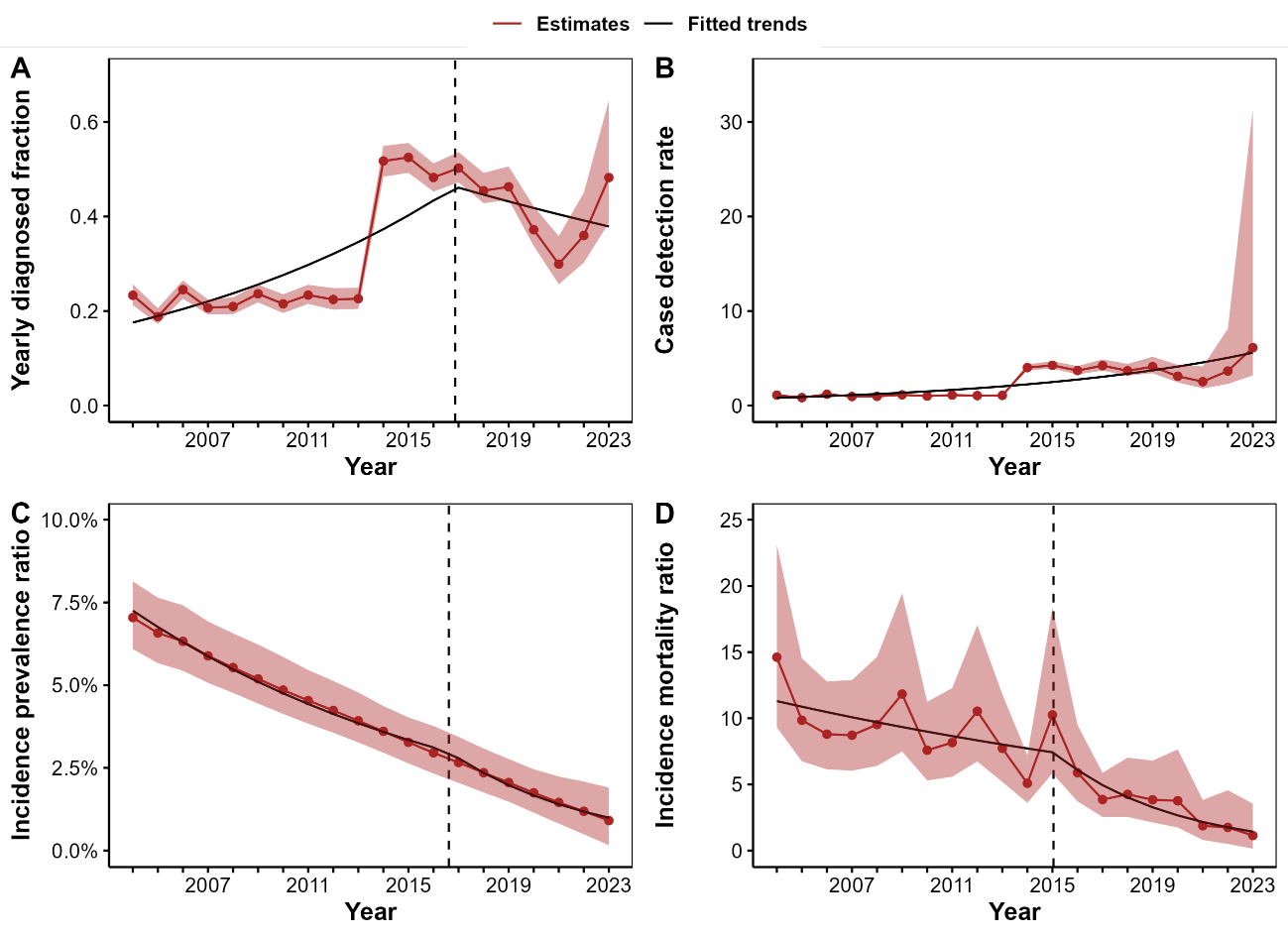


**Figure S13: Trends in other cascade related metrics between 2004-2023 for females.** A) YDF, B) CDR, C) IPR, and D) IMR. Red lines and shading show the best estimated values and ranges from the HIV cascade calculations. Black lines show the best fitting piecewise negative binomial model predictions. Vertical dashed lines show the location of detected change points. The estimates for females are provided in Table S10.

# Regression model fitting

The figures in the following subsections show the resulting regression model fits for each number of change points (labelled A) and the selected best fitting model (labelled B; the same figure as in the main manuscript) compared to the estimates for each epidemiological metric (labelled on the y-axis) for the population overall, males, and females. The resulting BIC value and the smallest gap between change points for each model is shown in the top figure. If the segmented algorithm fails for the specified number of change points, then it is replaced with the overall model meaning the BIC values will be the same as for the overall model and the gap between change points will be infinite (designated by ‘Inf’ in the figure). The locations of the change points for the selected model are shown as dashed vertical lines in the bottom figures.

## Figures for the overall population

**Fitted models for the number of people living with HIV (A) and the best fitting two change point (CP) model with estimated change points (vertical dashed lines)**
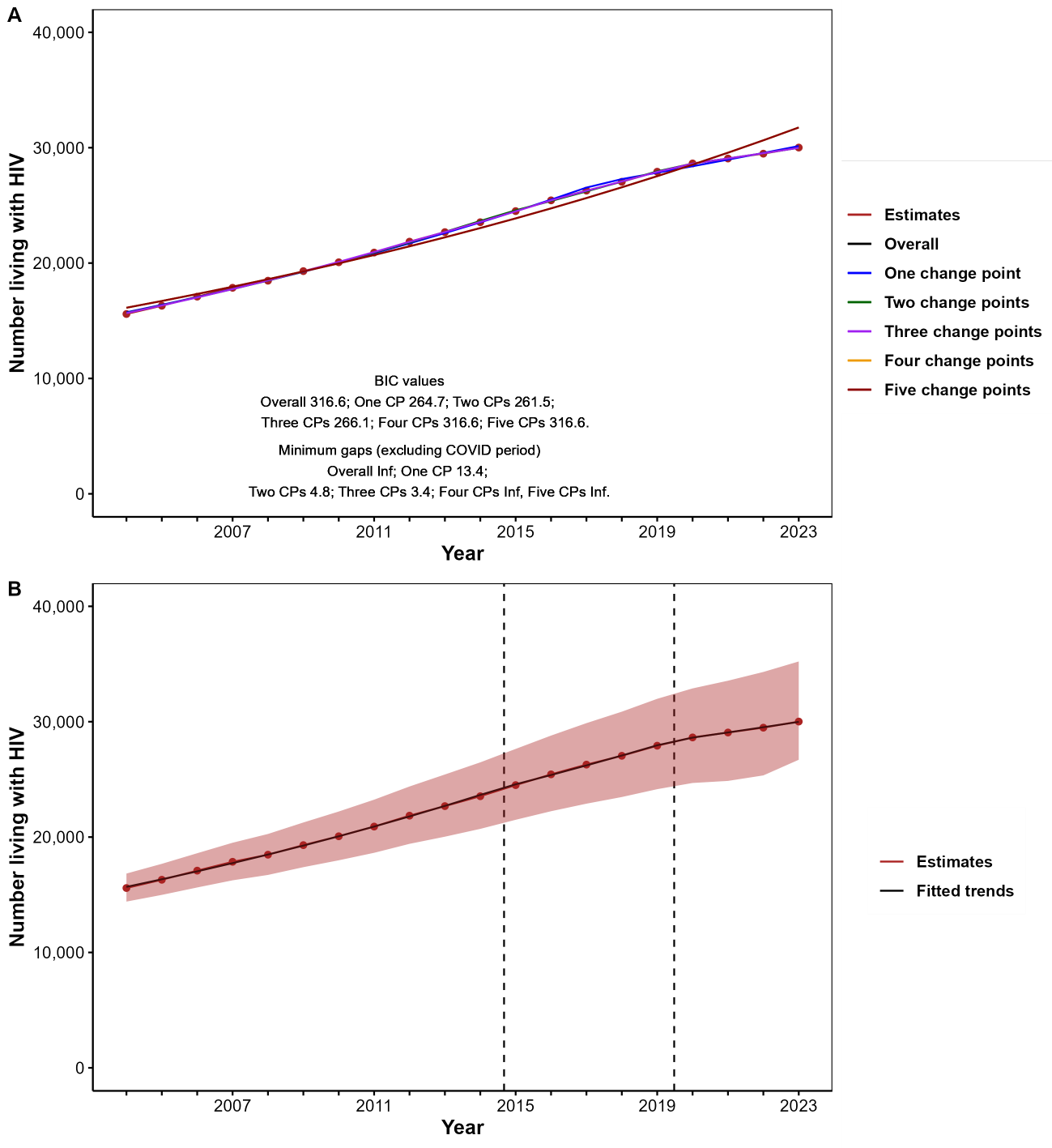


**Fitted models for the number of people living with diagnosed HIV (A) and the best fitting two change point model (B) with estimated change points (vertical dashed lines)**
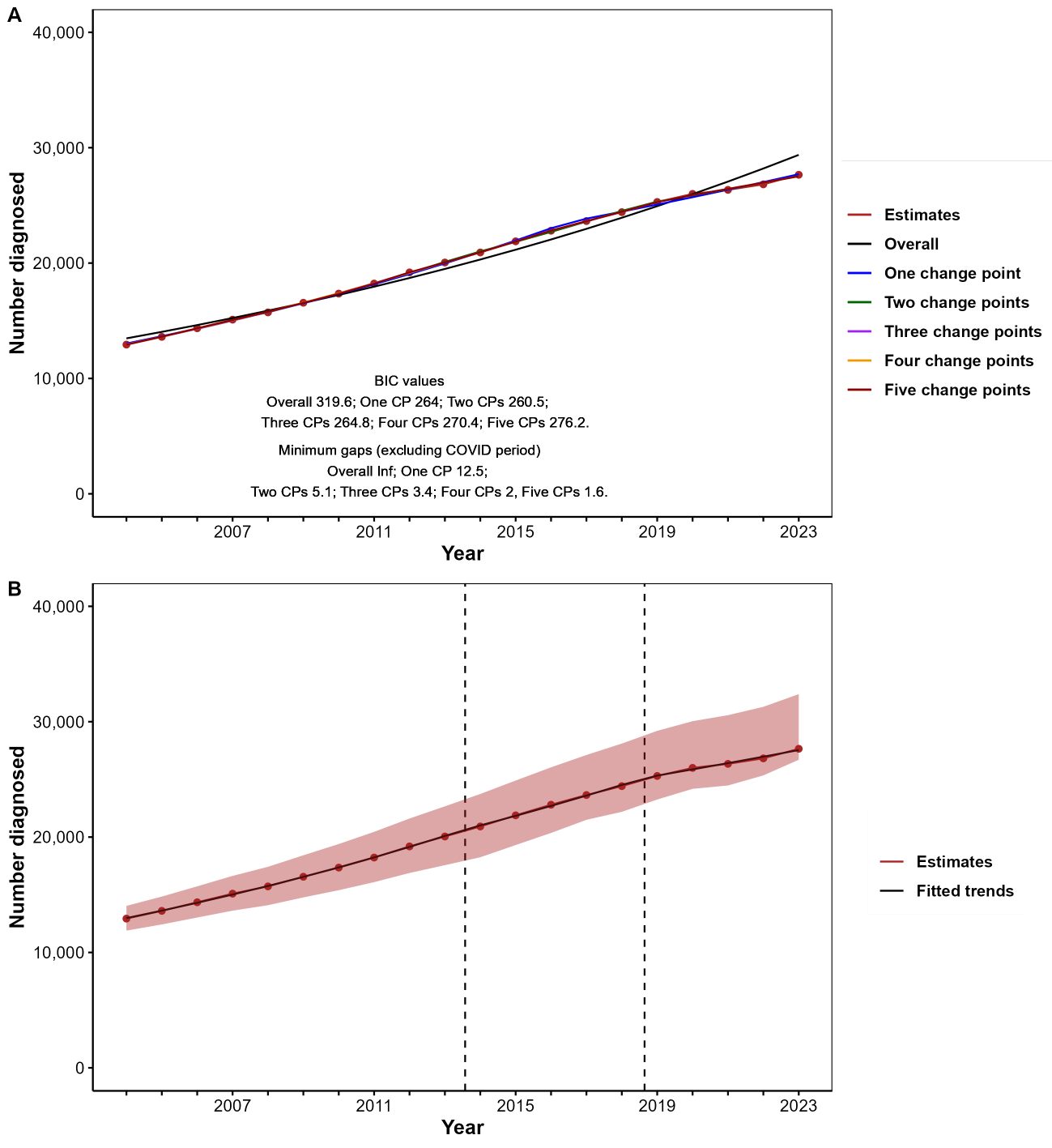


**Fitted models for the number of people living with HIV on ART (A) and the best fitting two change point model (B) with estimated change points (vertical dashed lines)**
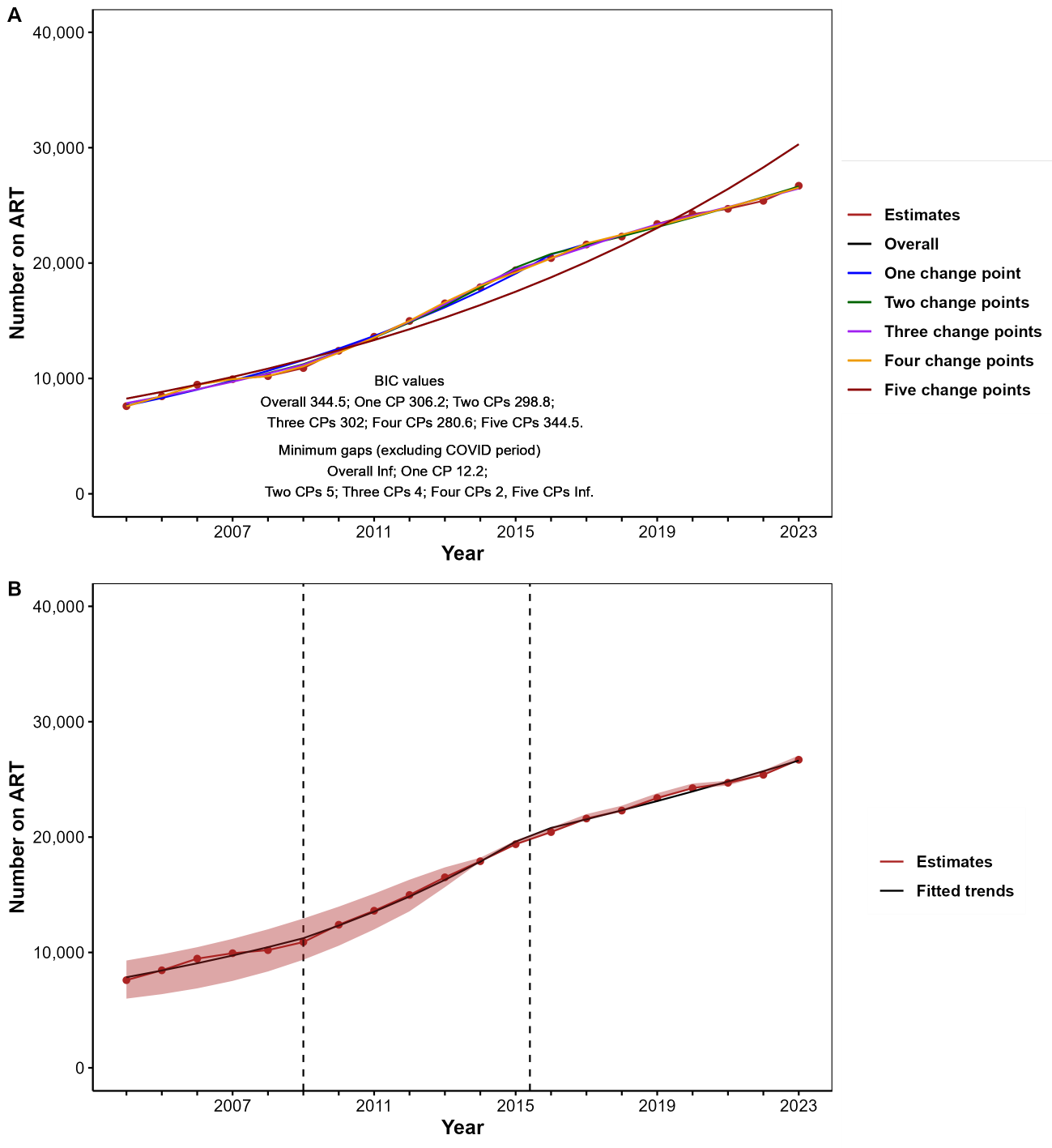


**Fitted models for the number of people with a suppressed viral load (A) and the best fitting one change point model (B) with estimated change points (vertical dashed lines)**
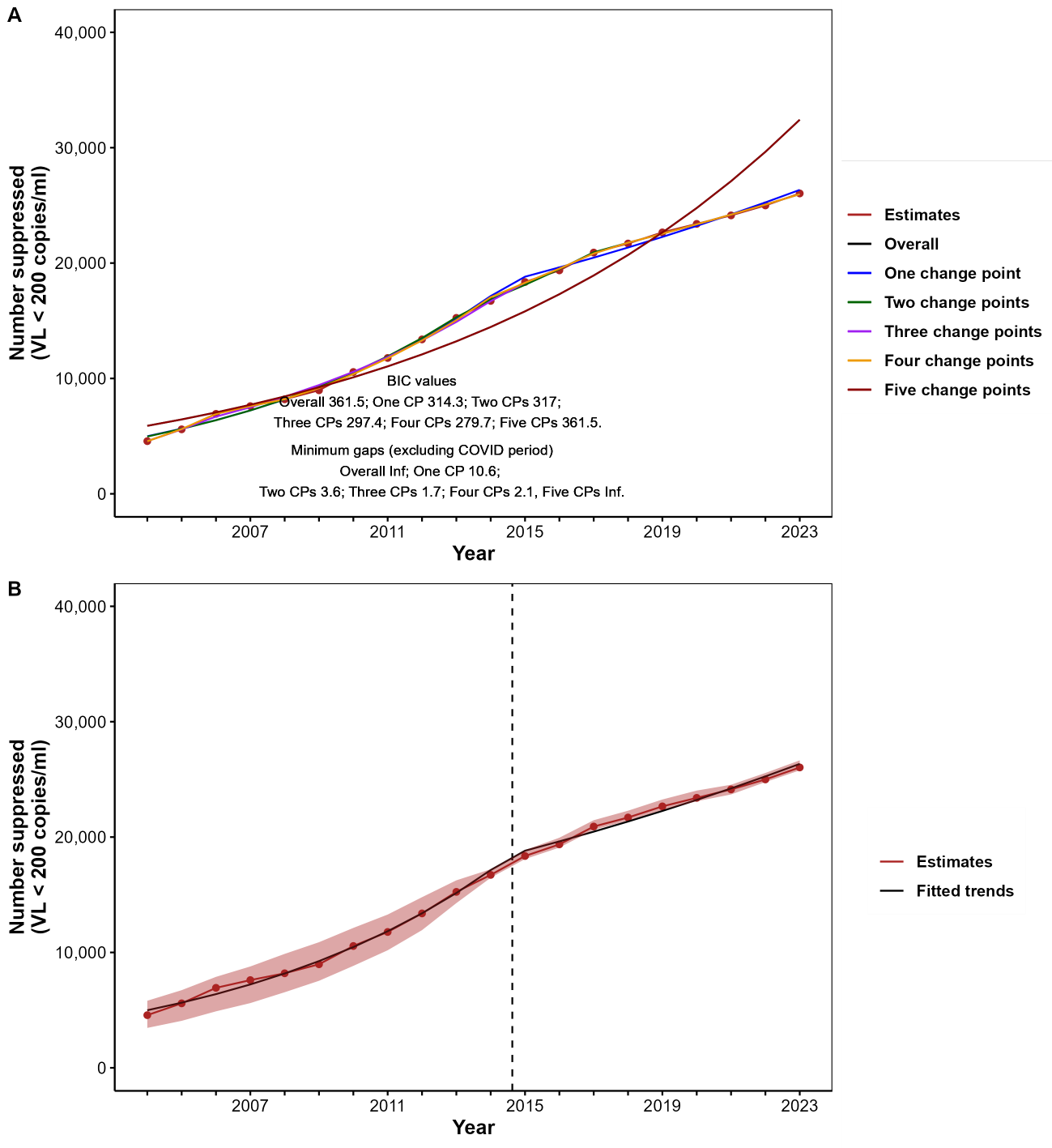


**Fitted models for the number of people living with undiagnosed HIV (A) and the best fitting two change point model (B) with estimated change points (vertical dashed lines)**
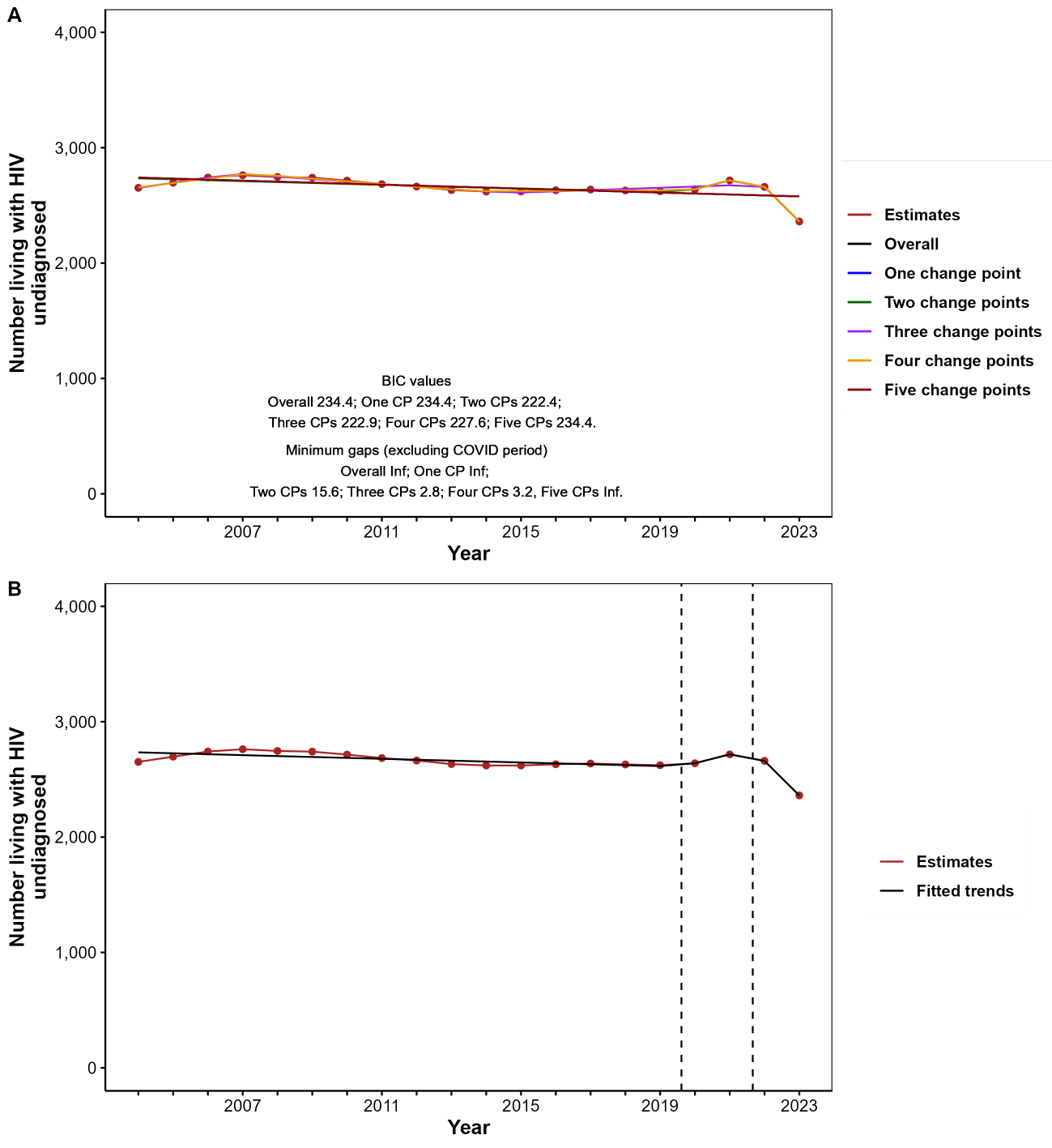


**Fitted models for the number of people diagnosed with HIV but not on ART (A) and the best fitting three change point model with estimated change points (vertical dashed lines)**
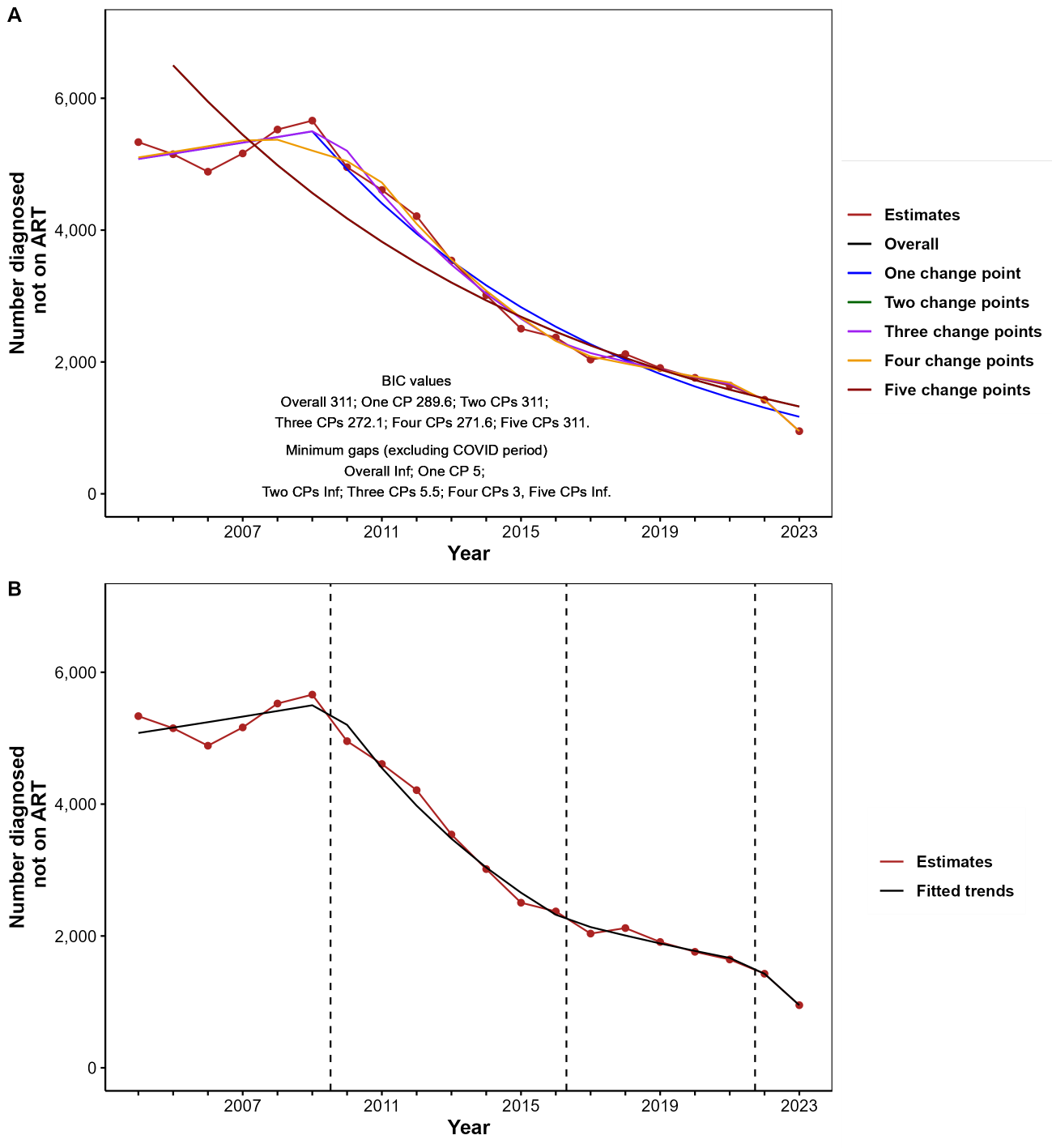


**Fitted models for the number of people on ART but with an unsuppressed viral load (A) and the best fitting overall model (B)**
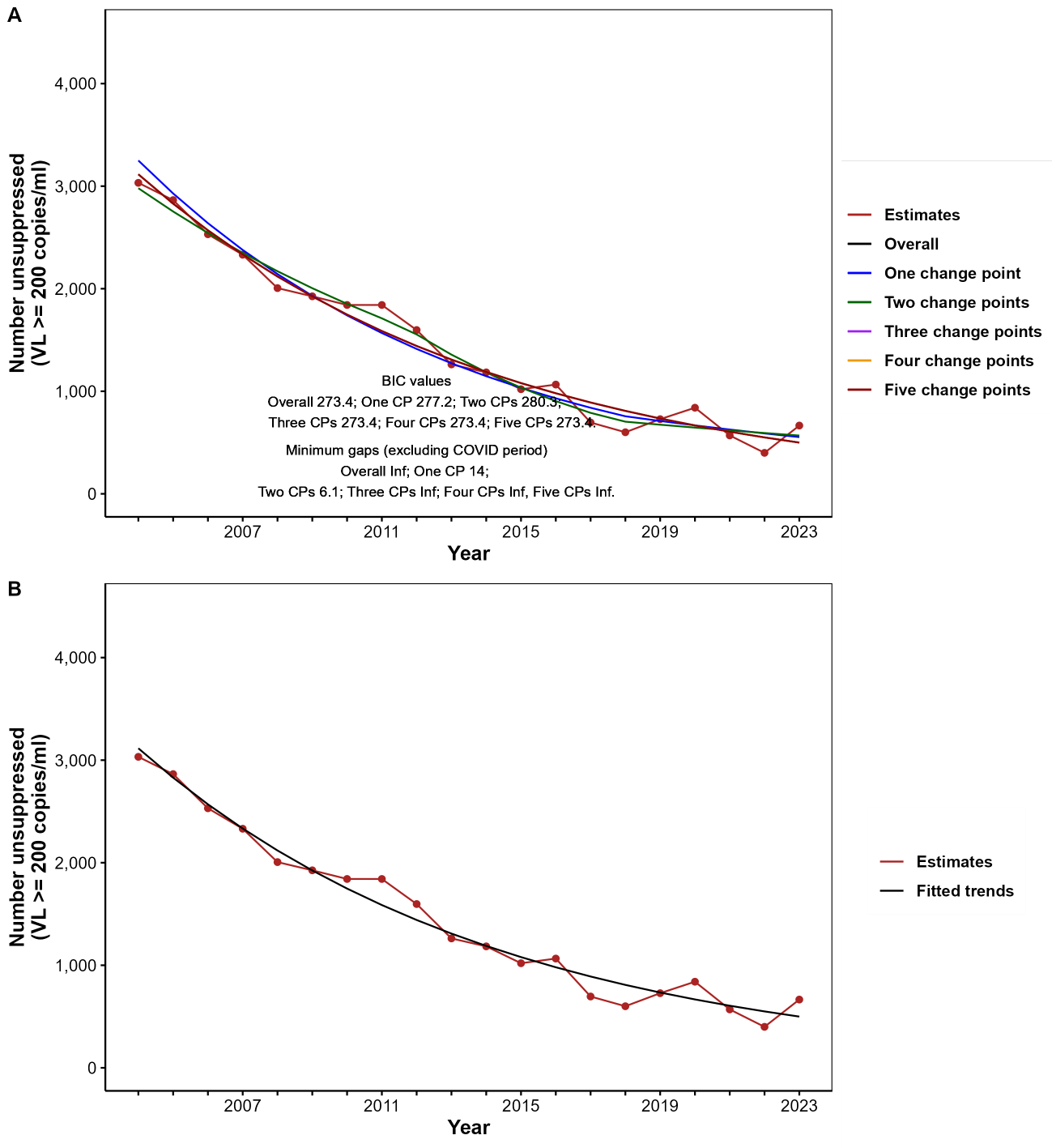


**Fitted models for the percentage of people living with HIV diagnosed (A) and the best fitting one change point model (B) with the estimated change point (vertical dashed line)**
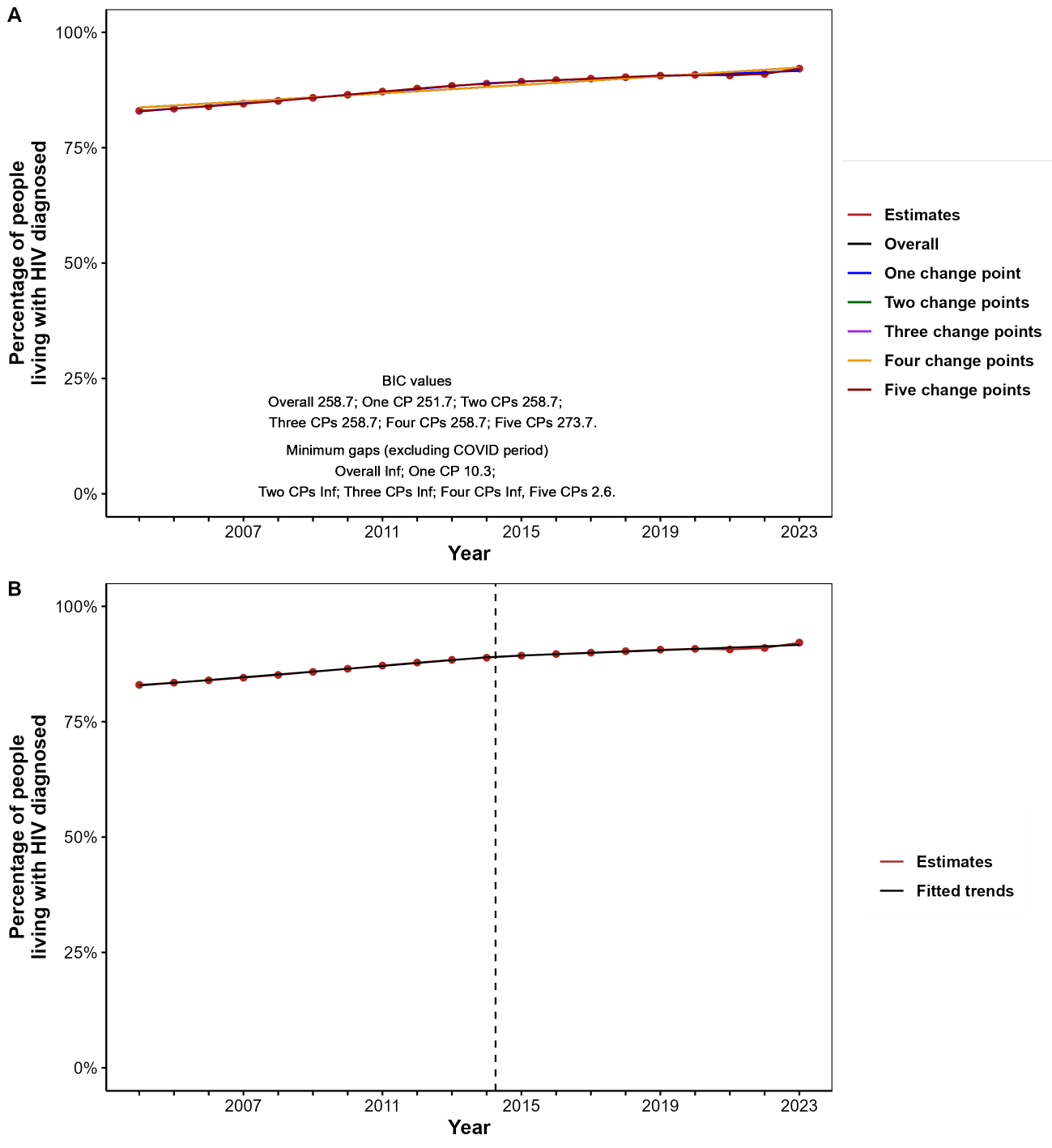


**Fitted models for the percentage of people diagnosed with HIV on ART (A) and the best fitting two change point model (B) with the estimated change points (vertical dashed lines)**
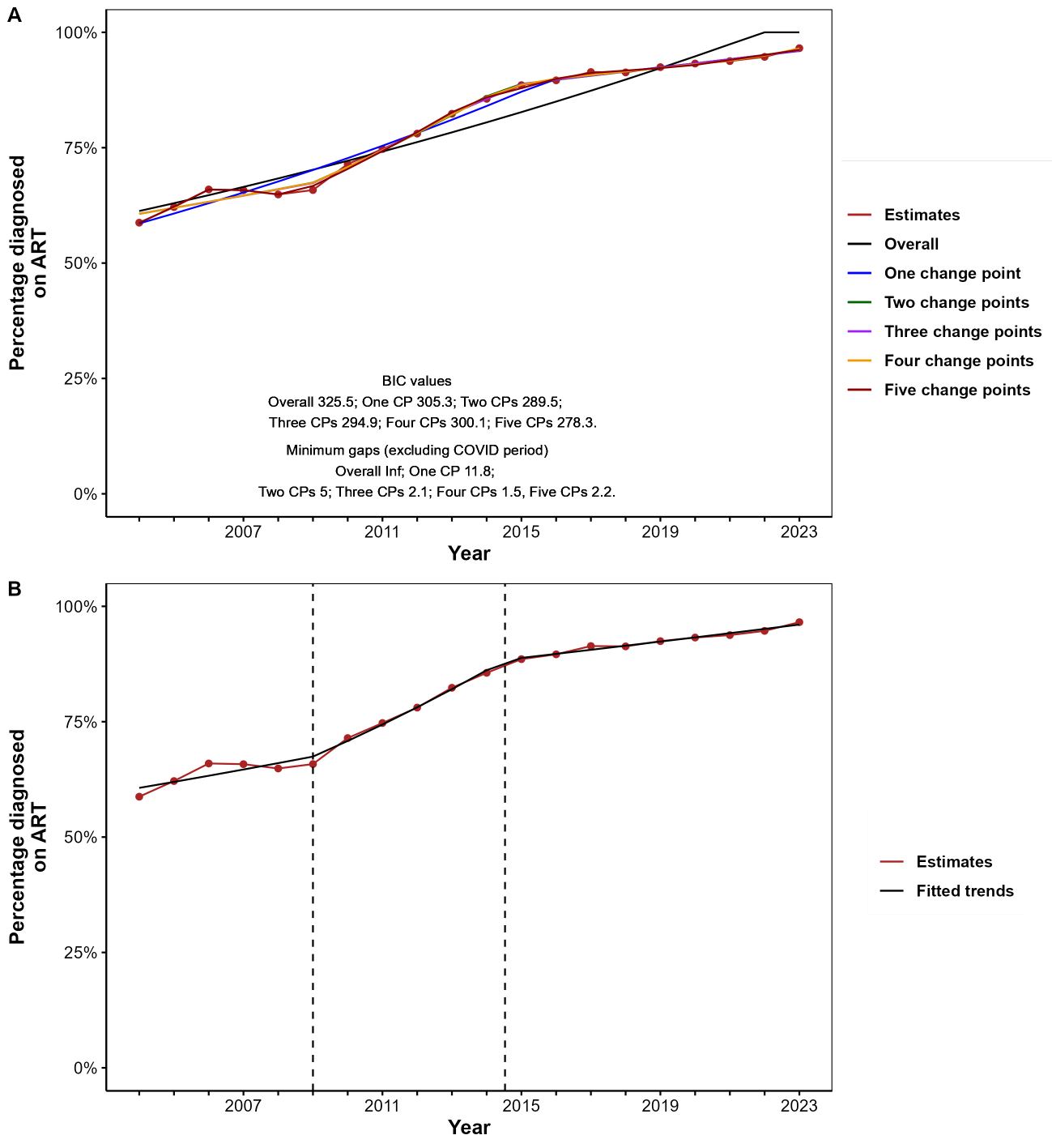


**Fitted models for the percentage of on ART with a suppressed viral load (A) and the best fitting one change point (CP) model (B) with the estimated change point (vertical dashed line)**
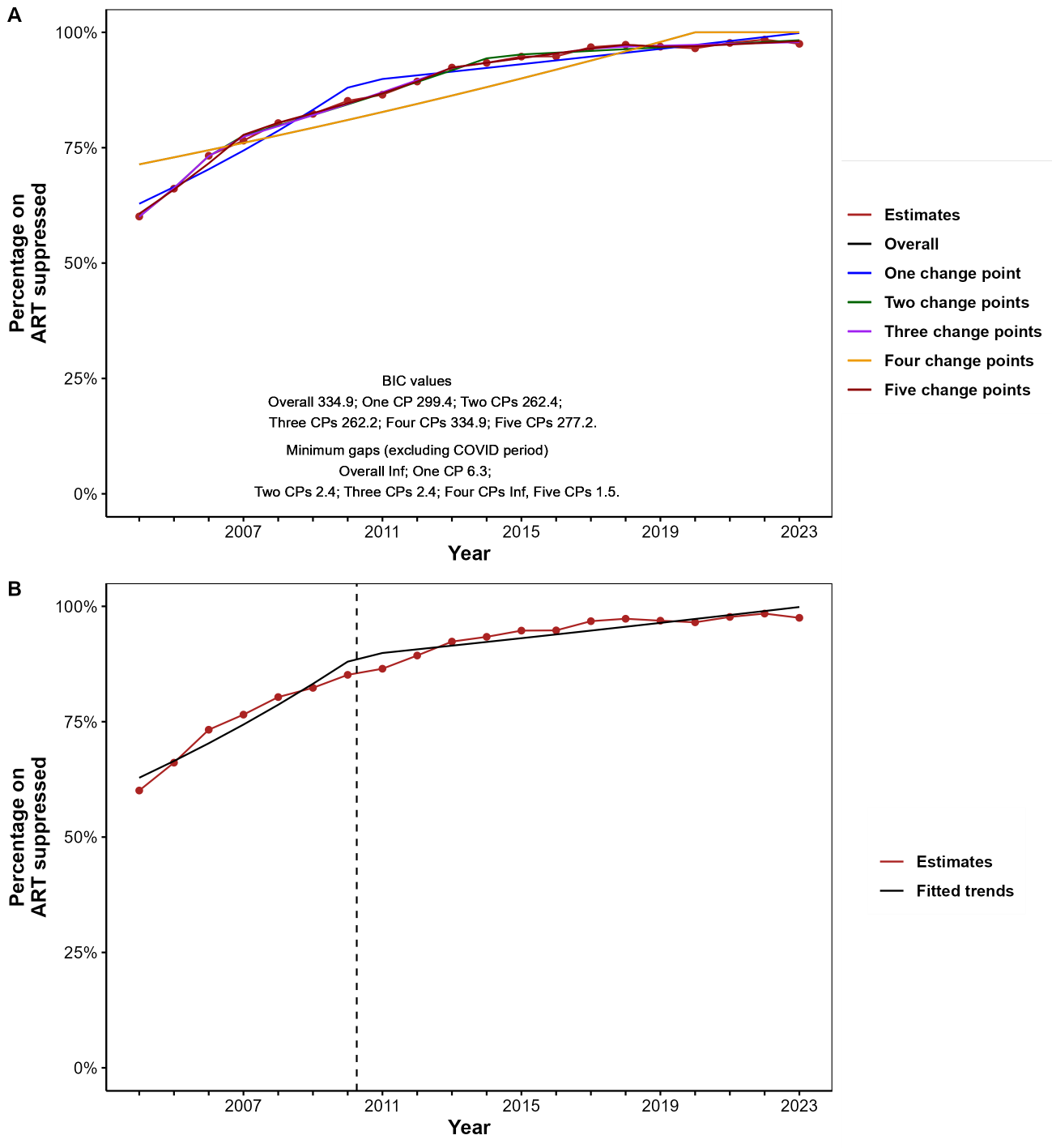


**Fitted models for the percentage of all people living with HIV with a suppressed viral load (A) and the best fitting one change point (CP) model (B) with the estimated change points (vertical dashed line)**
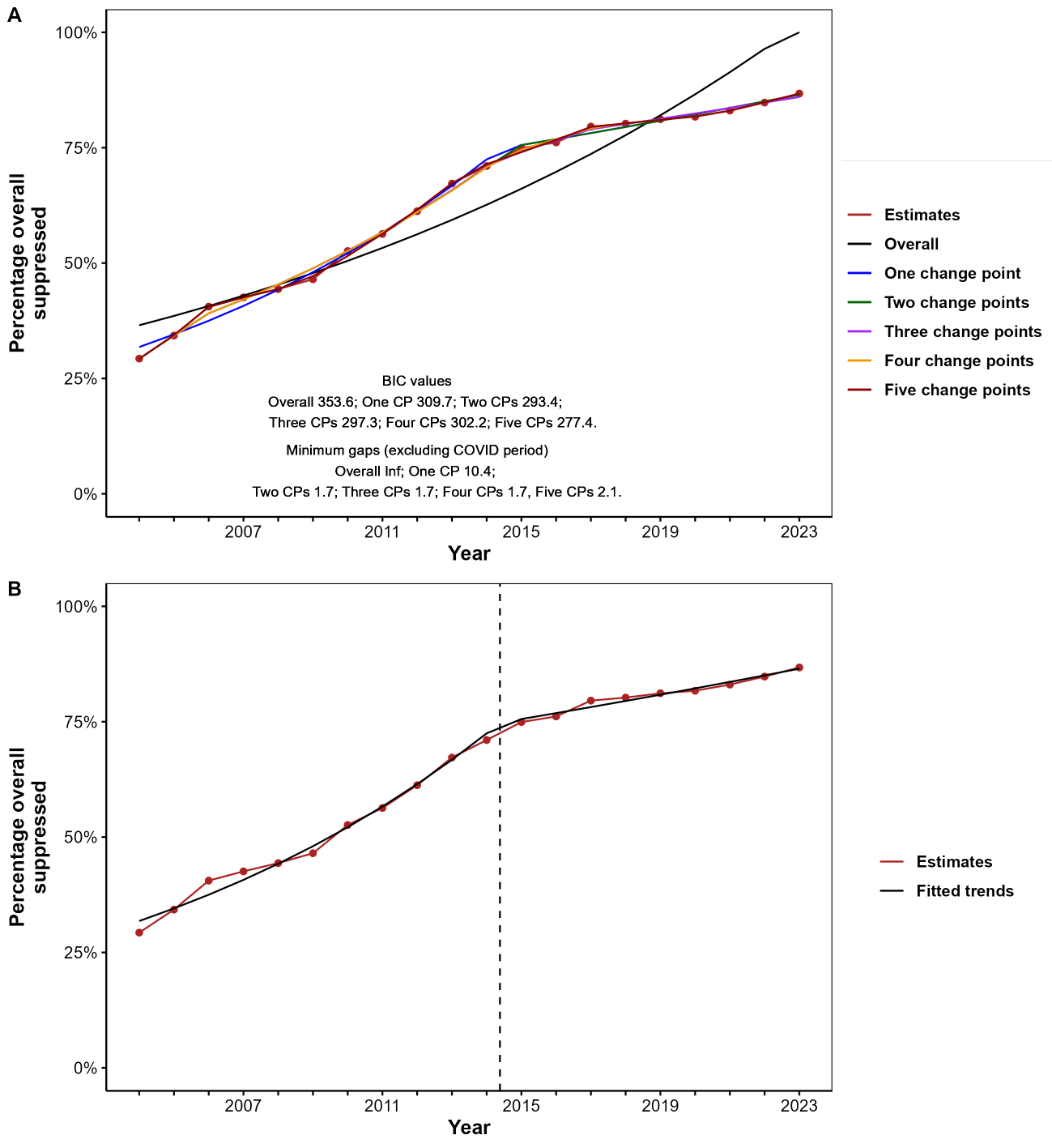


**Fitted models for the number of annual HIV notifications excluding people previously diagnosed overseas (A) and the best fitting three change point (CP) model (B) with the estimated change point (vertical dashed lines)**
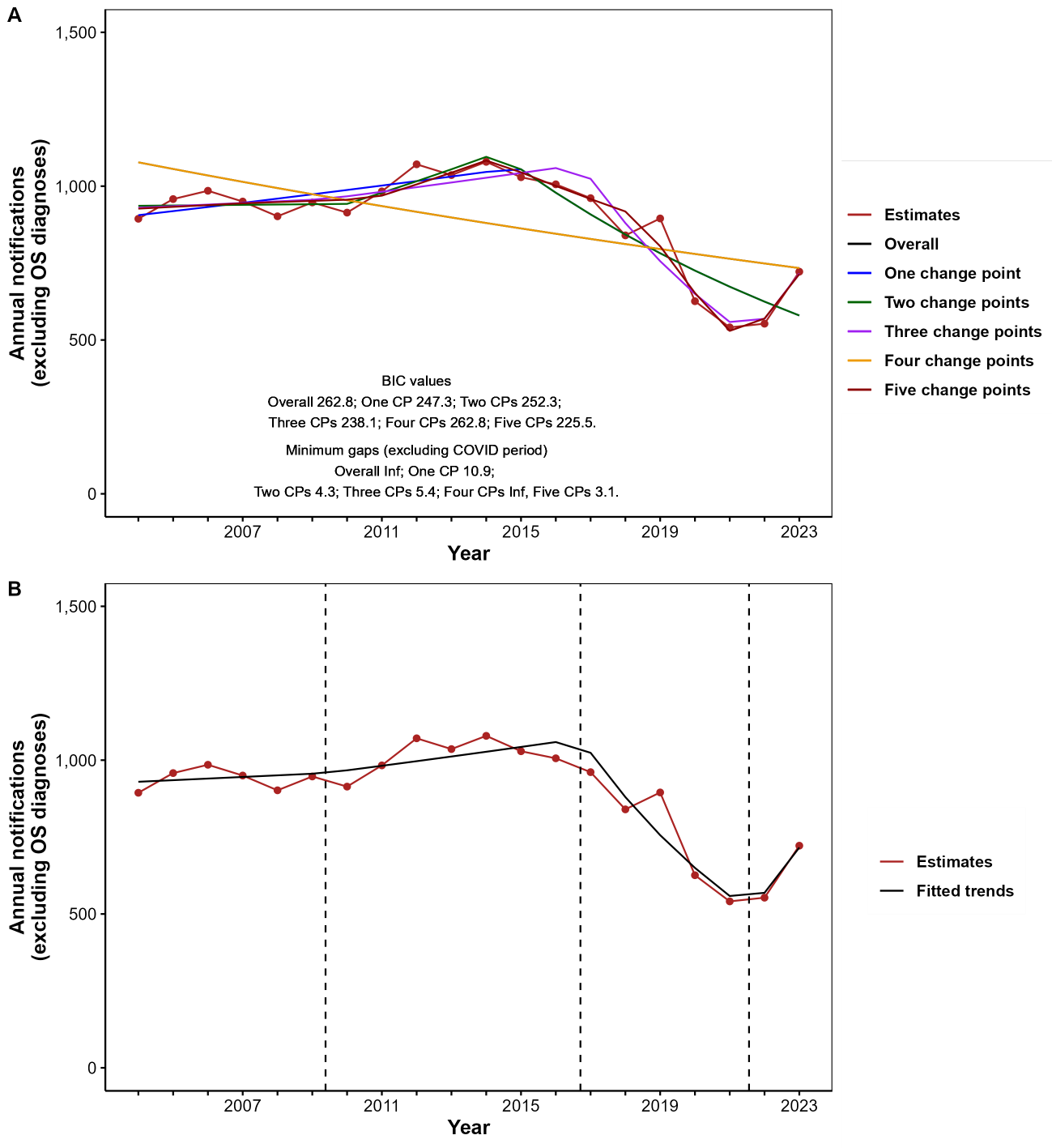


**Fitted models for the number of annual HIV notifications overall including people previously diagnosed overseas (OS) (A) and the best fitting three change point (CP) model (B) with the estimated change points (vertical dashed lines)**
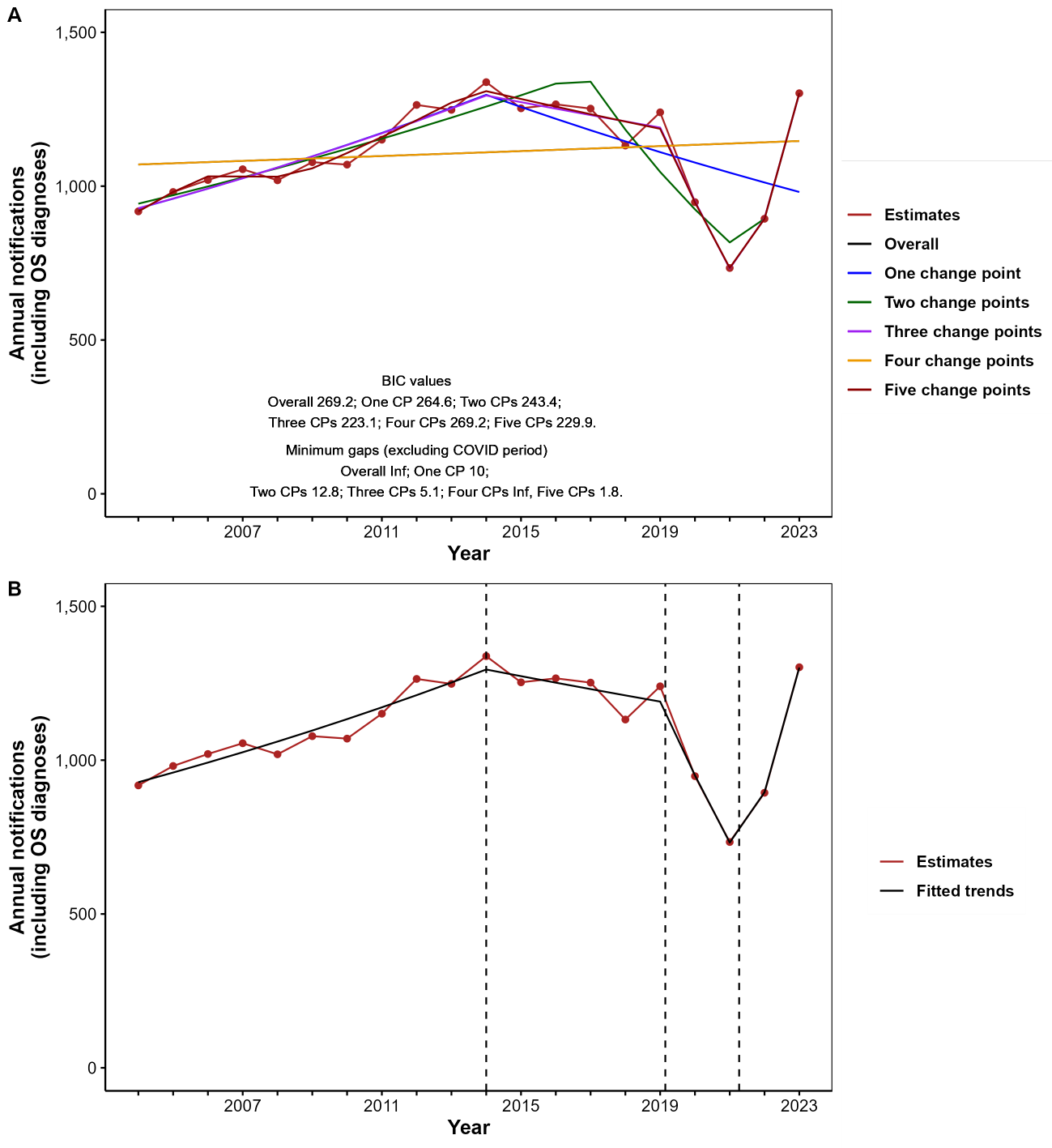


**Fitted models for the number of annual HIV notifications among people who have been previously diagnosed overseas (OS) (A) and the best fitting three change point (CP) model (B) with the estimated change points (vertical dashed lines)**
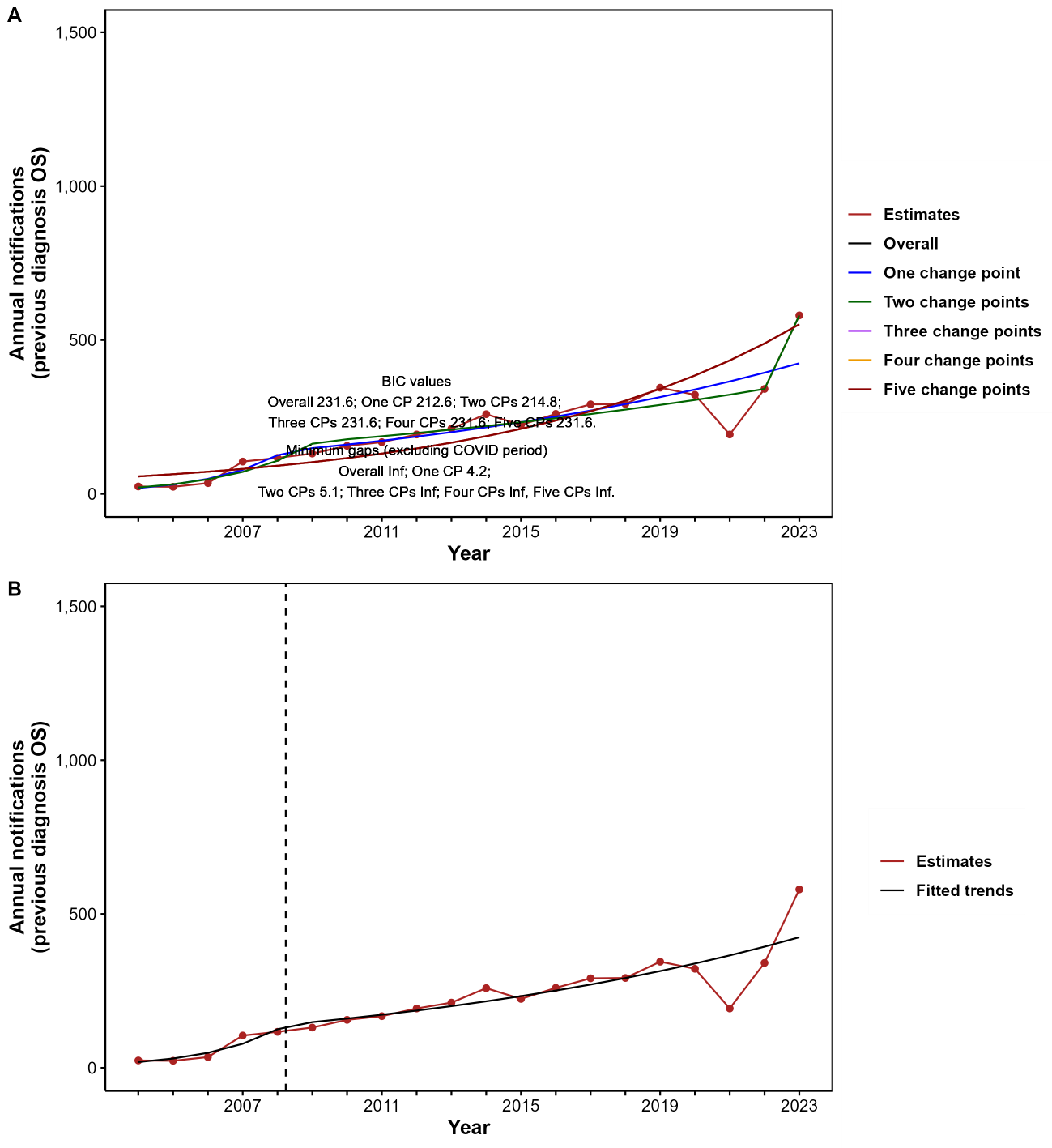


**Fitted models for the estimated number of annual new infections (A) and the best fitting two change point (CP) model (B) with the estimated change points (vertical dashed lines)**
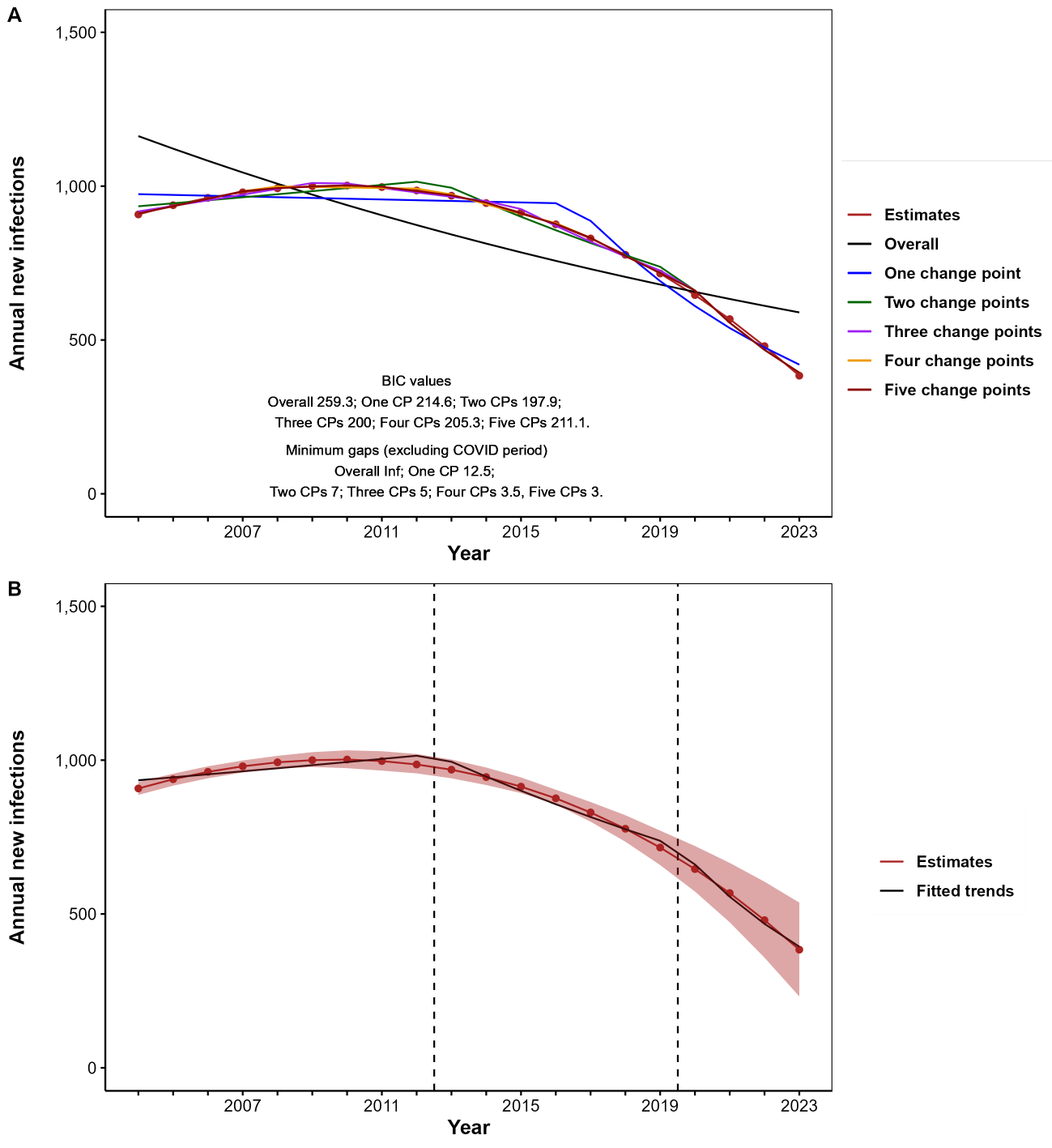


**Fitted models for the yearly diagnosed fraction (YDF) (A) and the best fitting two change point (CP) model (B) with the estimated change points (vertical dashed lines)**
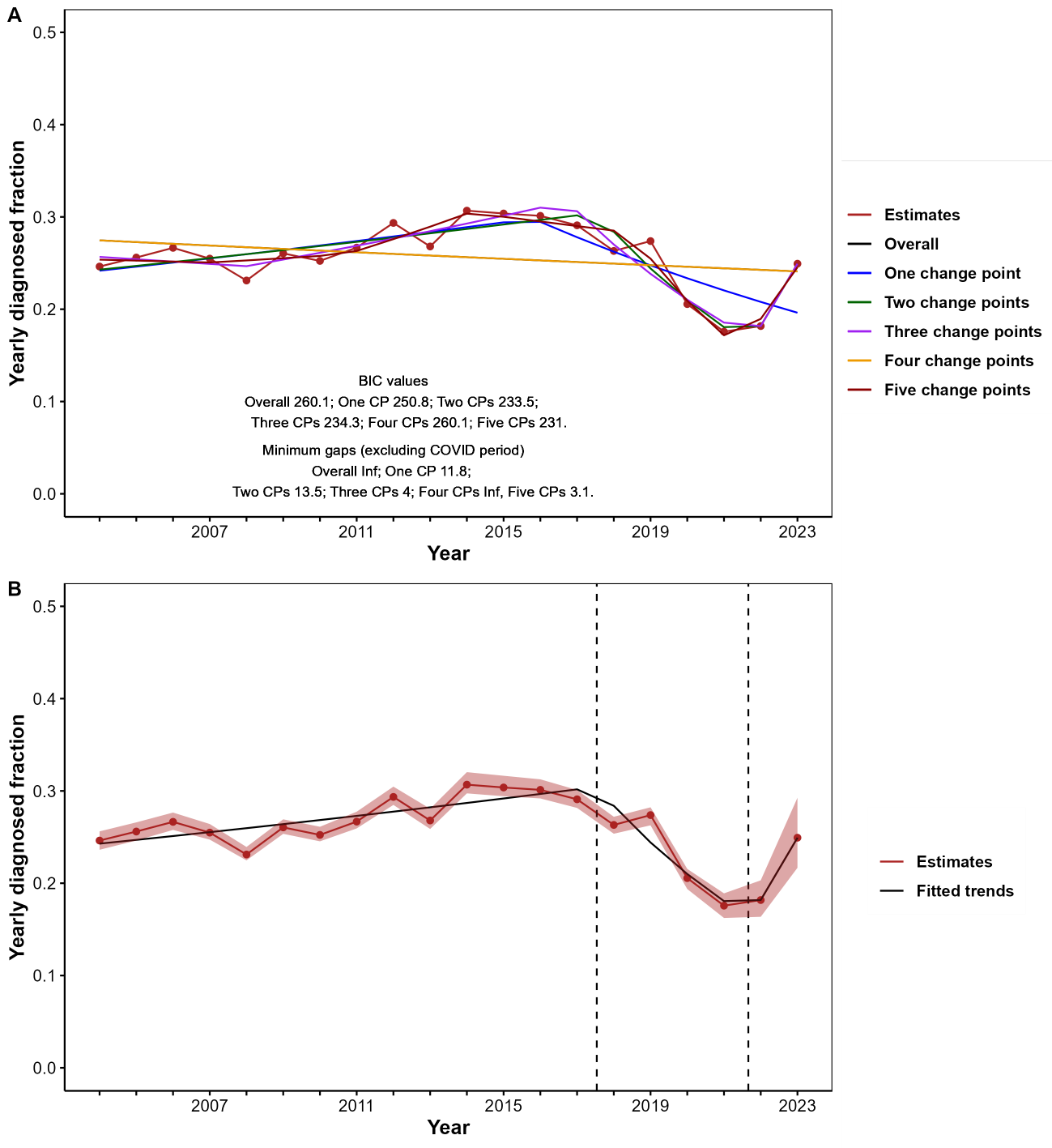


**Fitted models for the case detection rate (CDR) (A) and the best fitting two change point (CP) model (B) with the estimated change points (vertical dashed lines)**
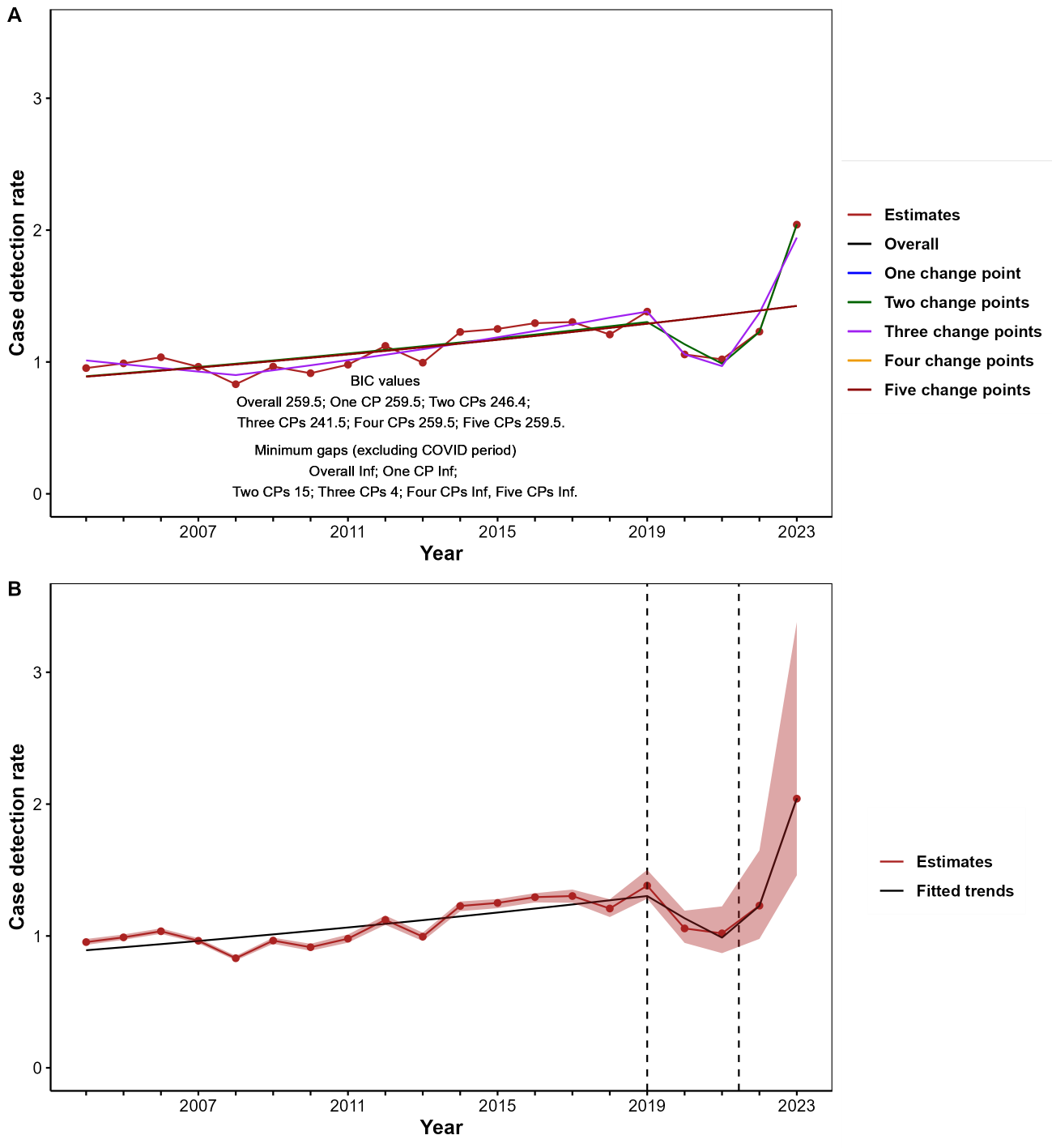


**Fitted models for the incidence prevalence ratio (IPR) (A) and the best fitting two change point (CP) model (B) with the estimated change points (vertical dashed lines)**
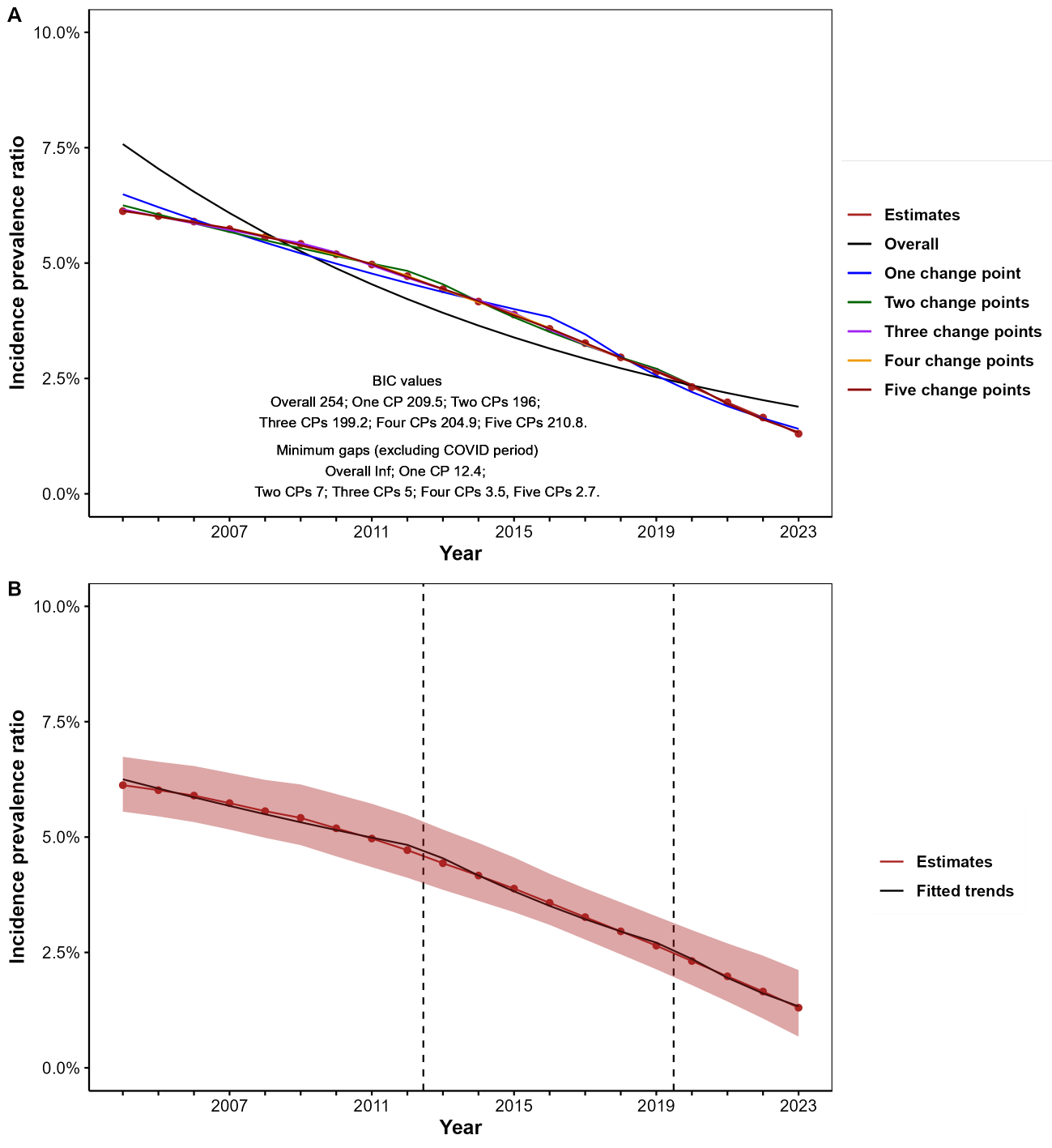


**Fitted models for the incidence mortality ratio (IMR) (A) and the best fitting one change point (CP) model (B) with the estimated change point (vertical dashed line)**
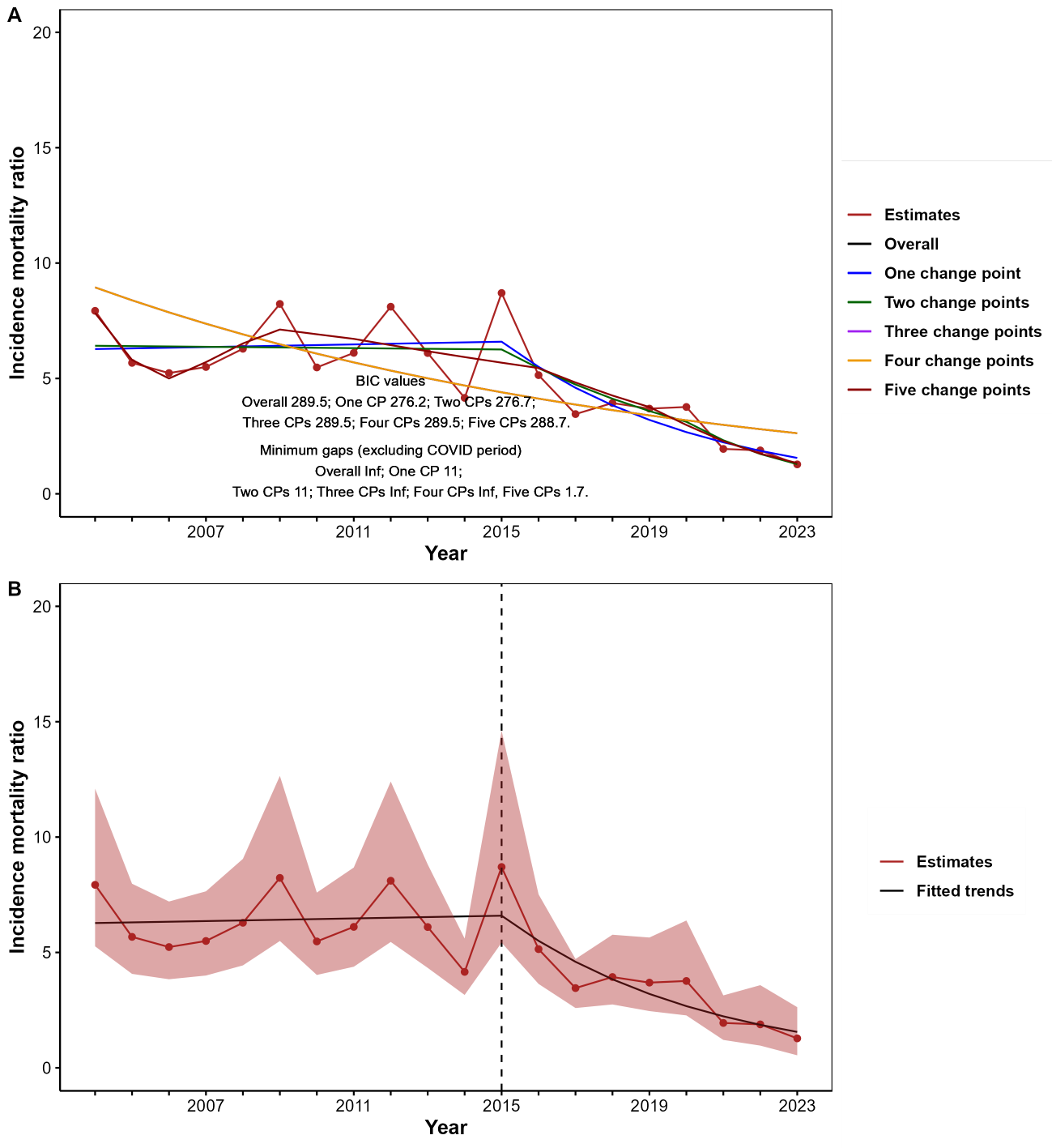


## Figures for males

**Fitted models for the number of people living with HIV (A) and the best fitting two change point (CP) model with estimated change points (vertical dashed lines)**
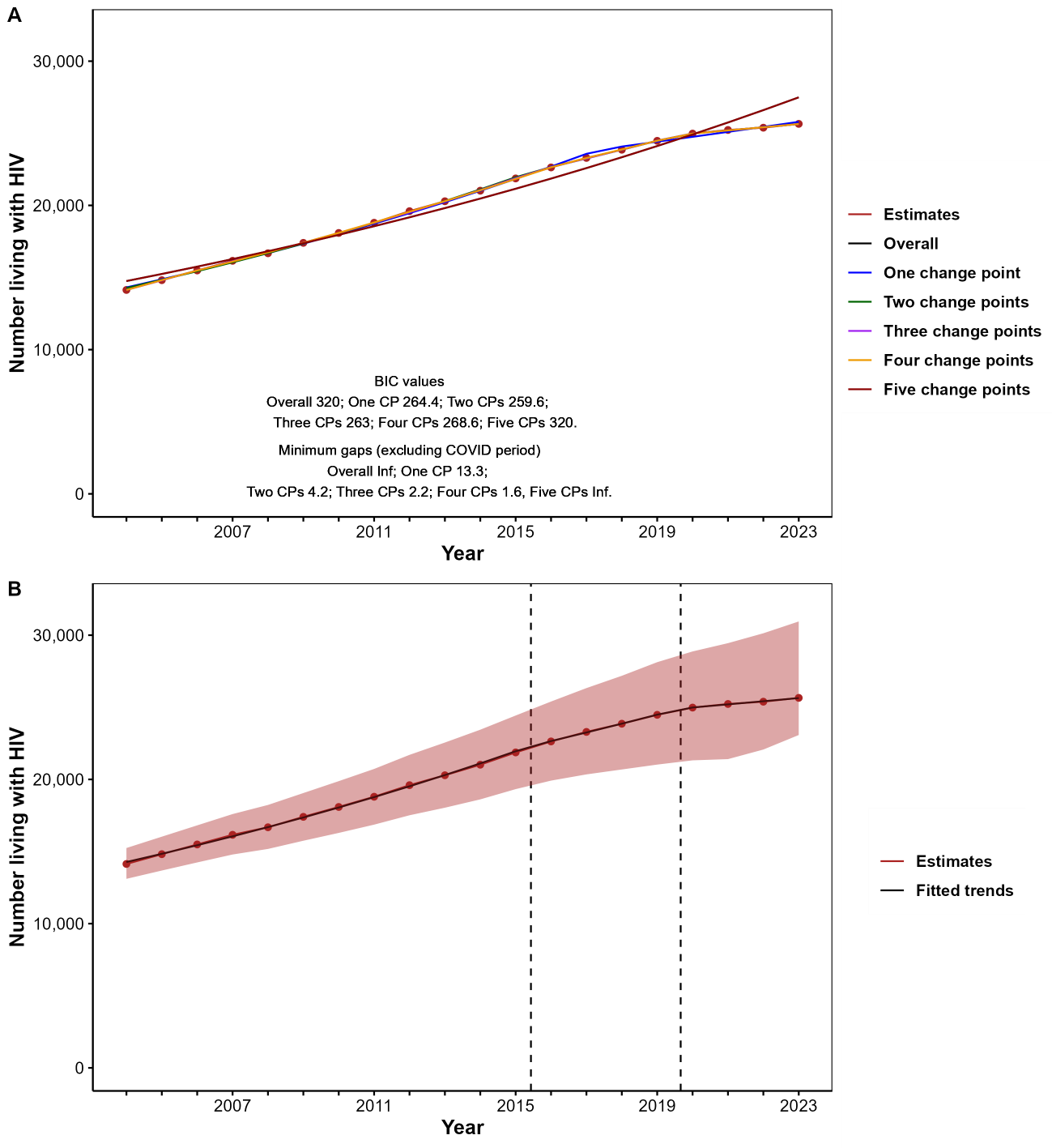


**Fitted models for the number of people living with diagnosed HIV (A) and the best fitting two change point model (B) with estimated change points (vertical dashed lines)**
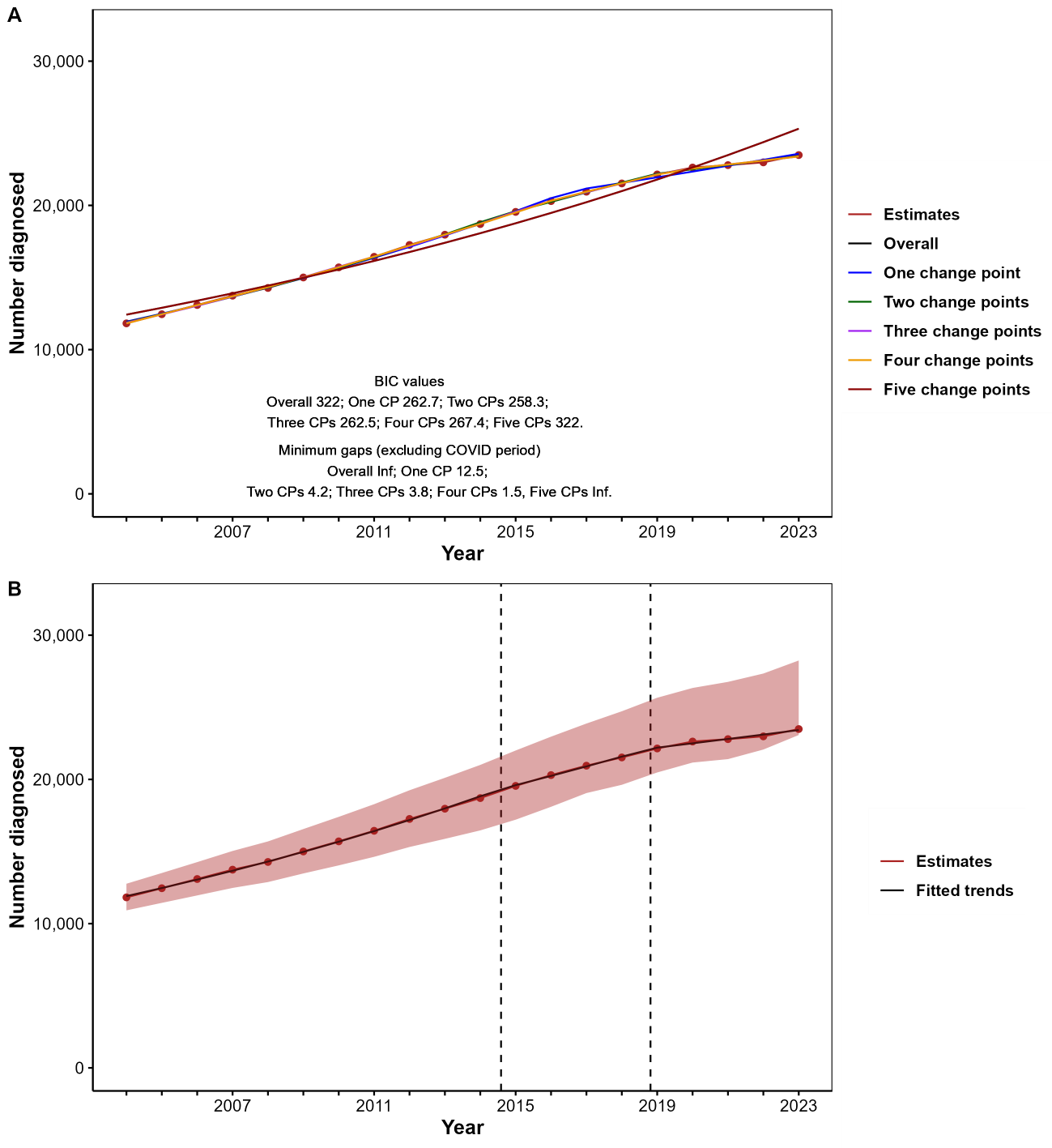


**Fitted models for the number of people living with HIV on ART (A) and the best fitting two change point model (B) with estimated change points (vertical dashed lines)**
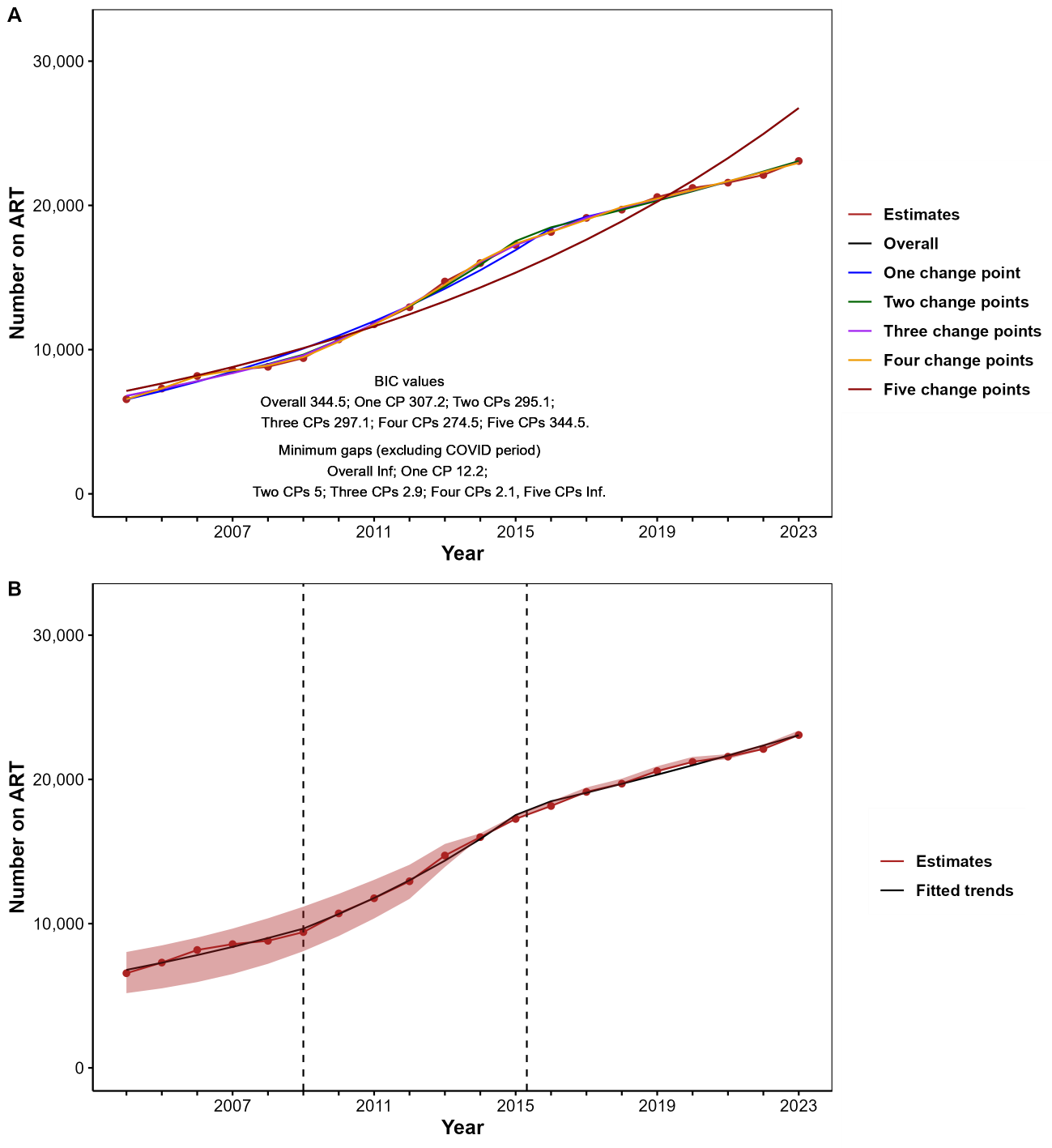


**Fitted models for the number of people with a suppressed viral load (A) and the best fitting one change point model (B) with estimated change points (vertical dashed lines)**
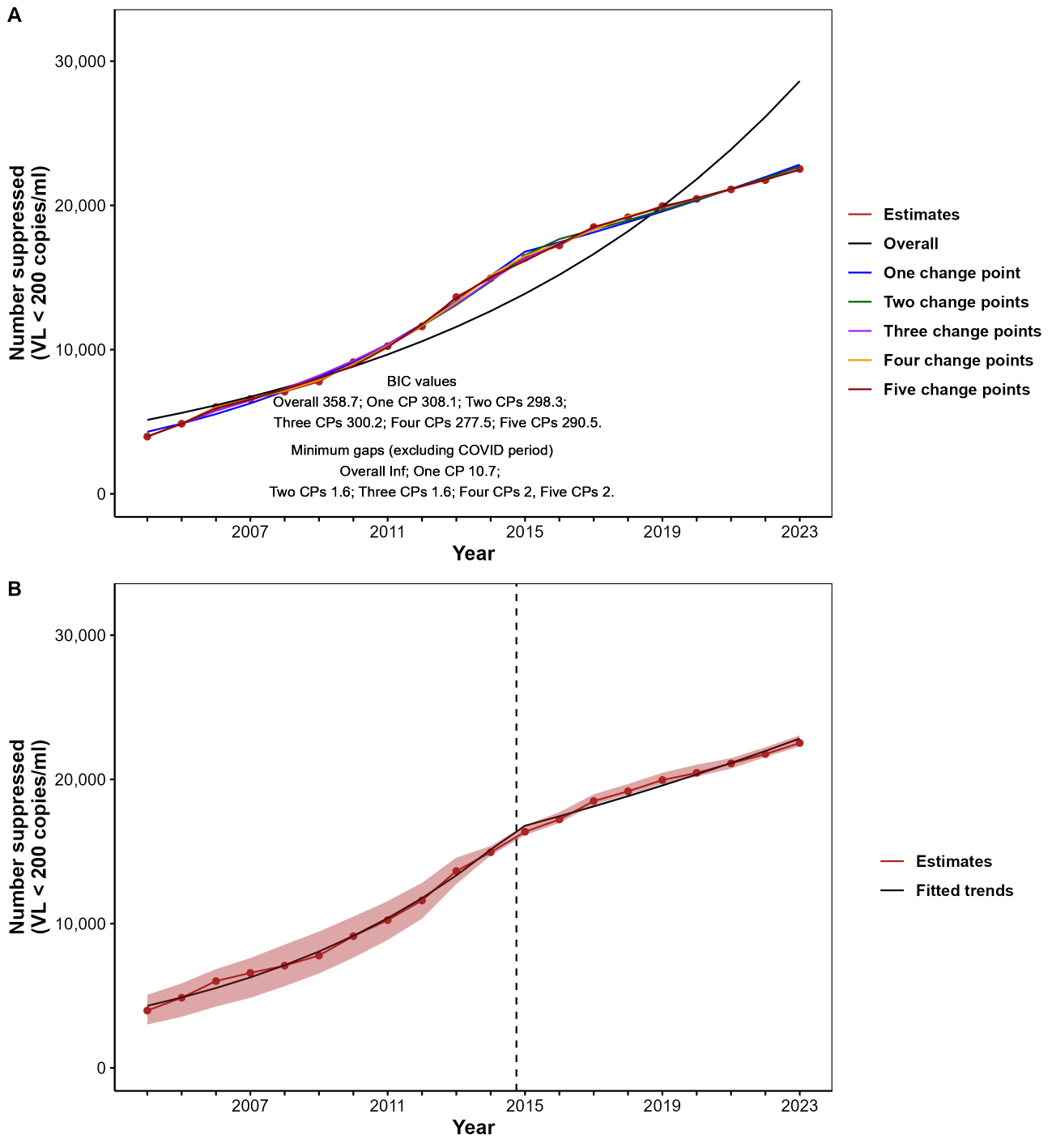


**Fitted models for the number of people living with undiagnosed HIV (A) and the best fitting two change point model (B) with estimated change points (vertical dashed lines)**
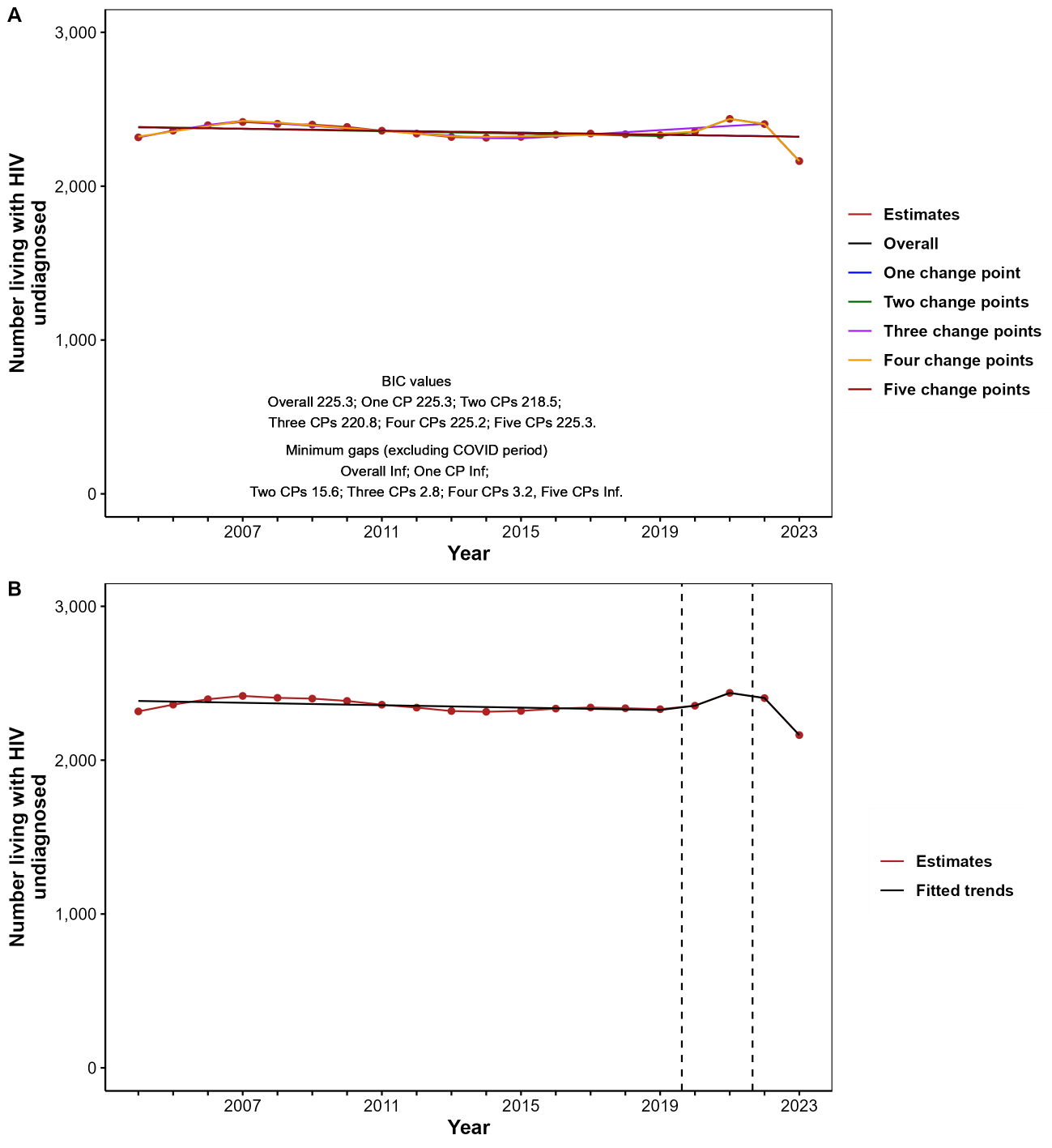


**Fitted models for the number of people diagnosed with HIV but not on ART (A) and the best fitting three change point model with estimated change points (vertical dashed lines)**
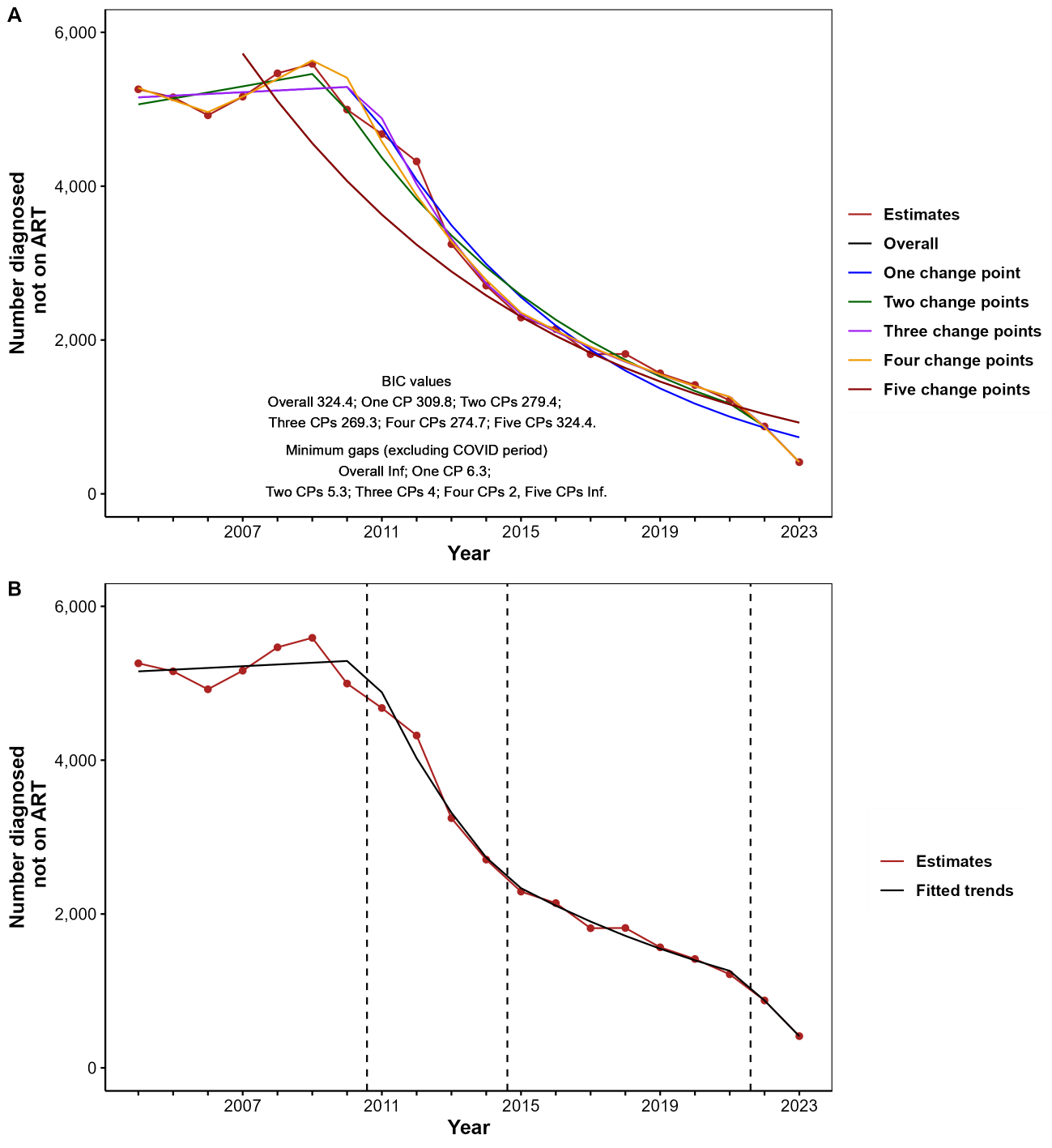


**Fitted models for the number of people on ART but with an unsuppressed viral load (A) and the best fitting overall model (B)**
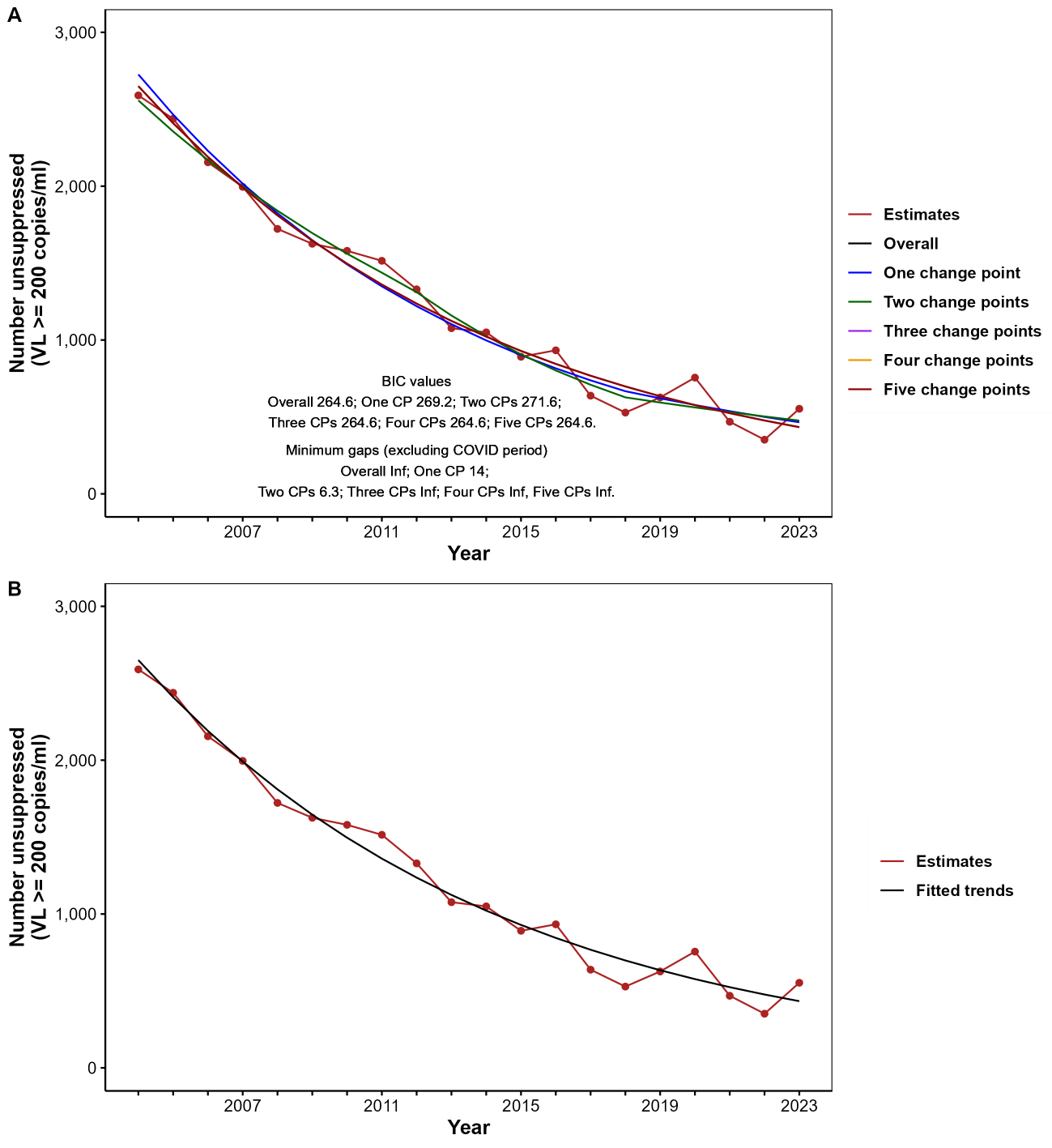


**Fitted models for the percentage of people living with HIV diagnosed (A) and the best fitting one change point model (B) with the estimated change point (vertical dashed line)**
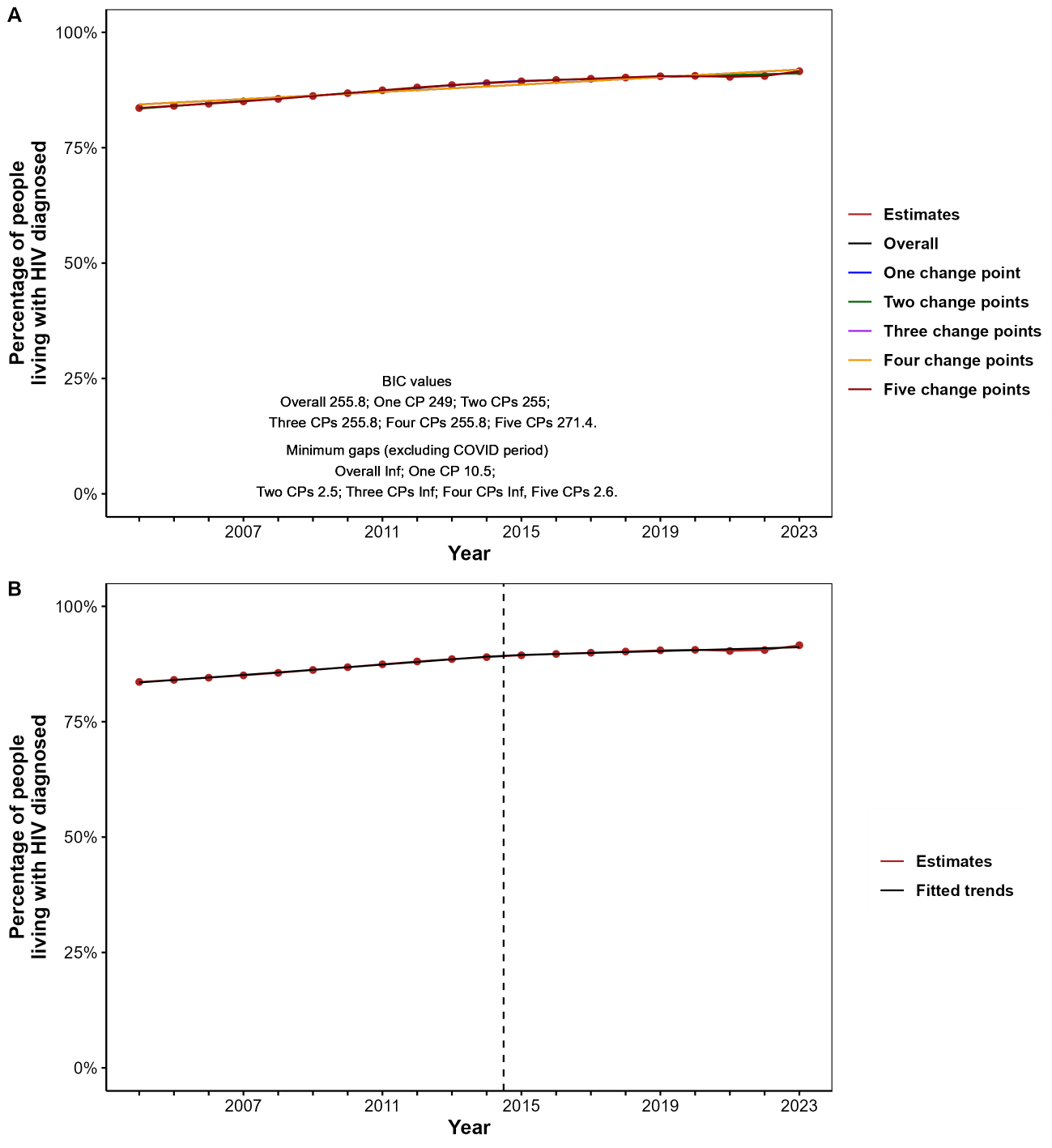


**Fitted models for the percentage of people diagnosed with HIV on ART (A) and the best fitting two change point model (B) with the estimated change points (vertical dashed lines)**
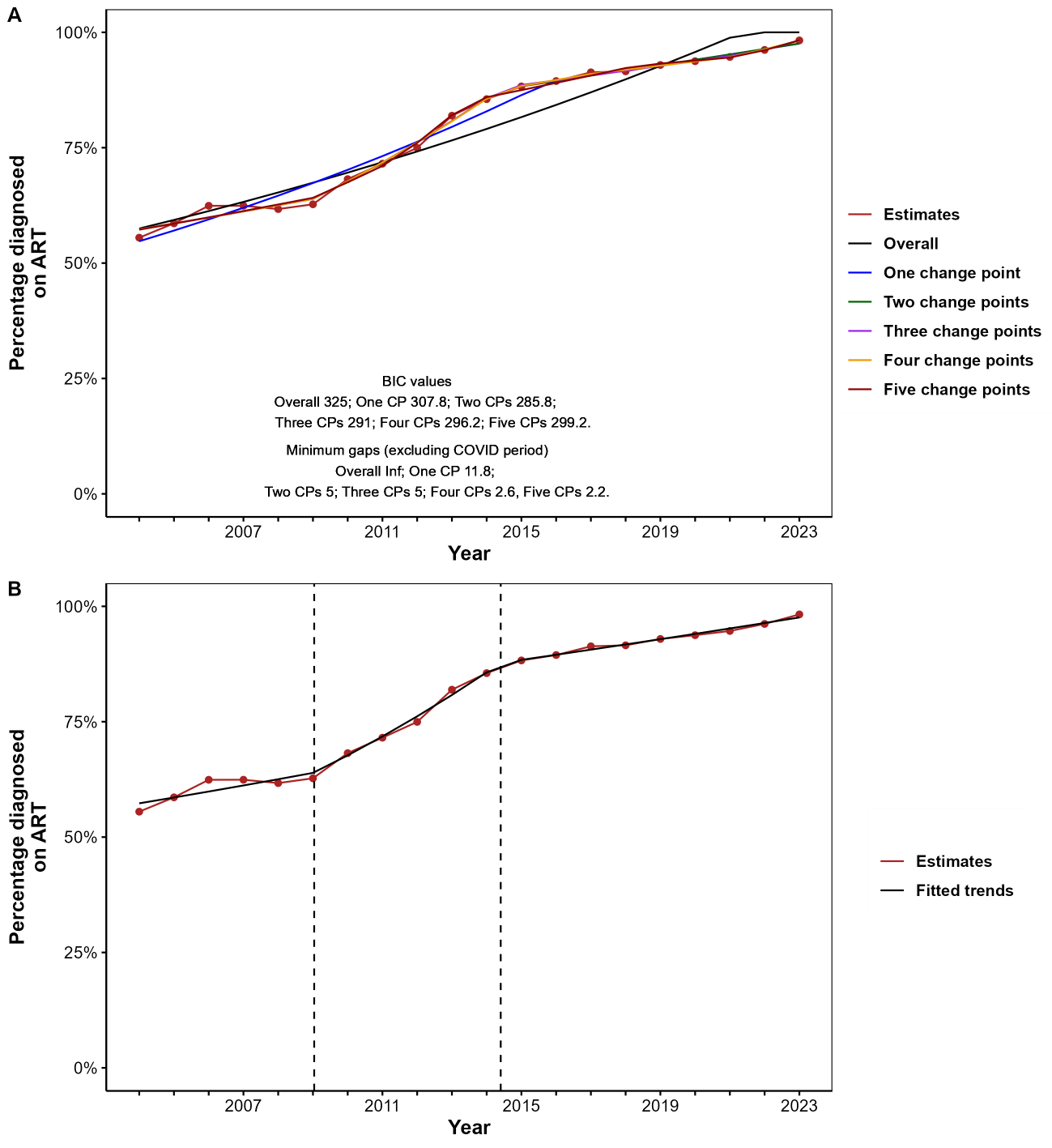


**Fitted models for the percentage of on ART with a suppressed viral load (A) and the best fitting one change point (CP) model (B) with the estimated change point (vertical dashed line)**
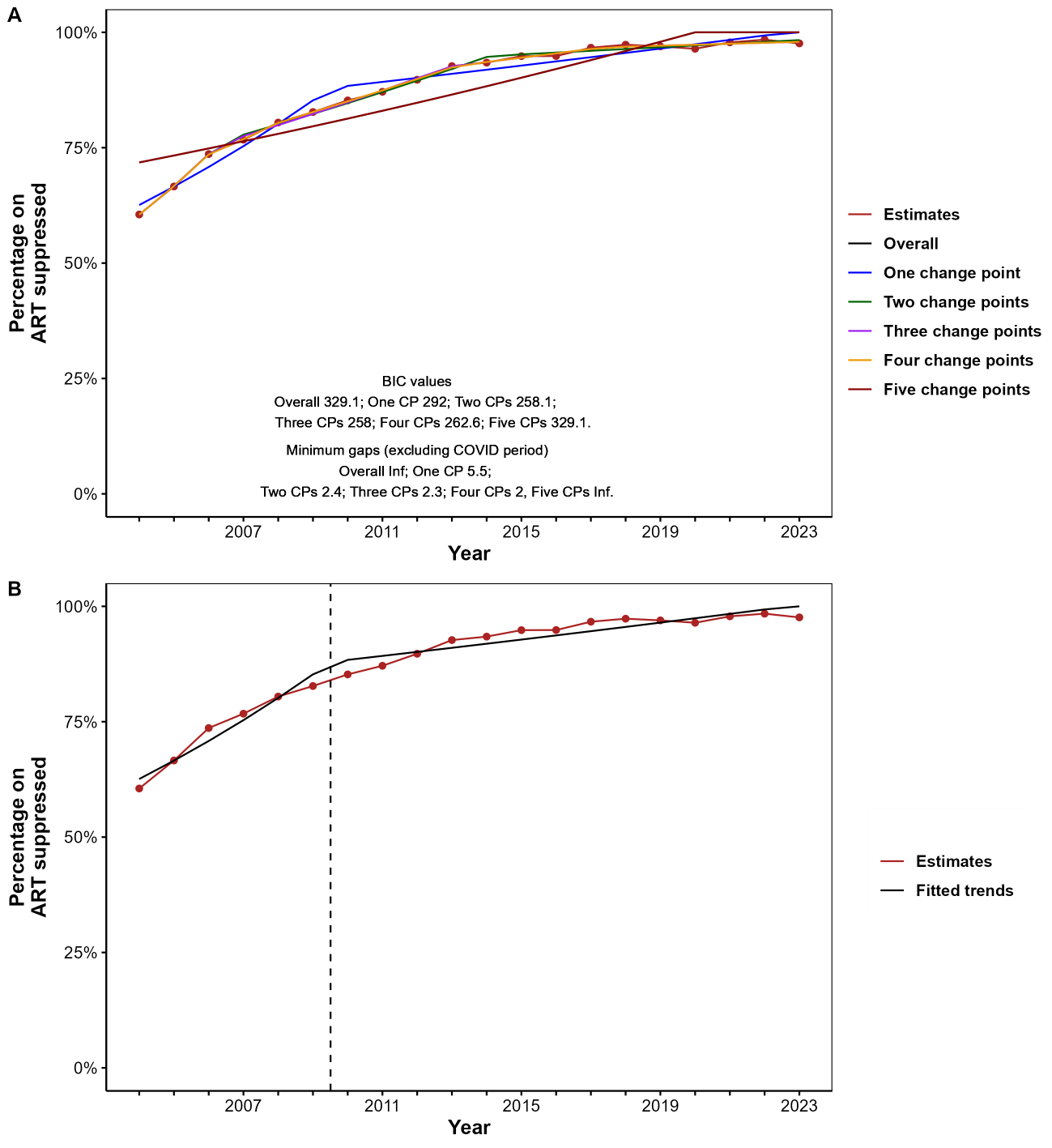


**Fitted models for the percentage of all people living with HIV with a suppressed viral load (A) and the best fitting one change point (CP) model (B) with the estimated change points (vertical dashed line)**
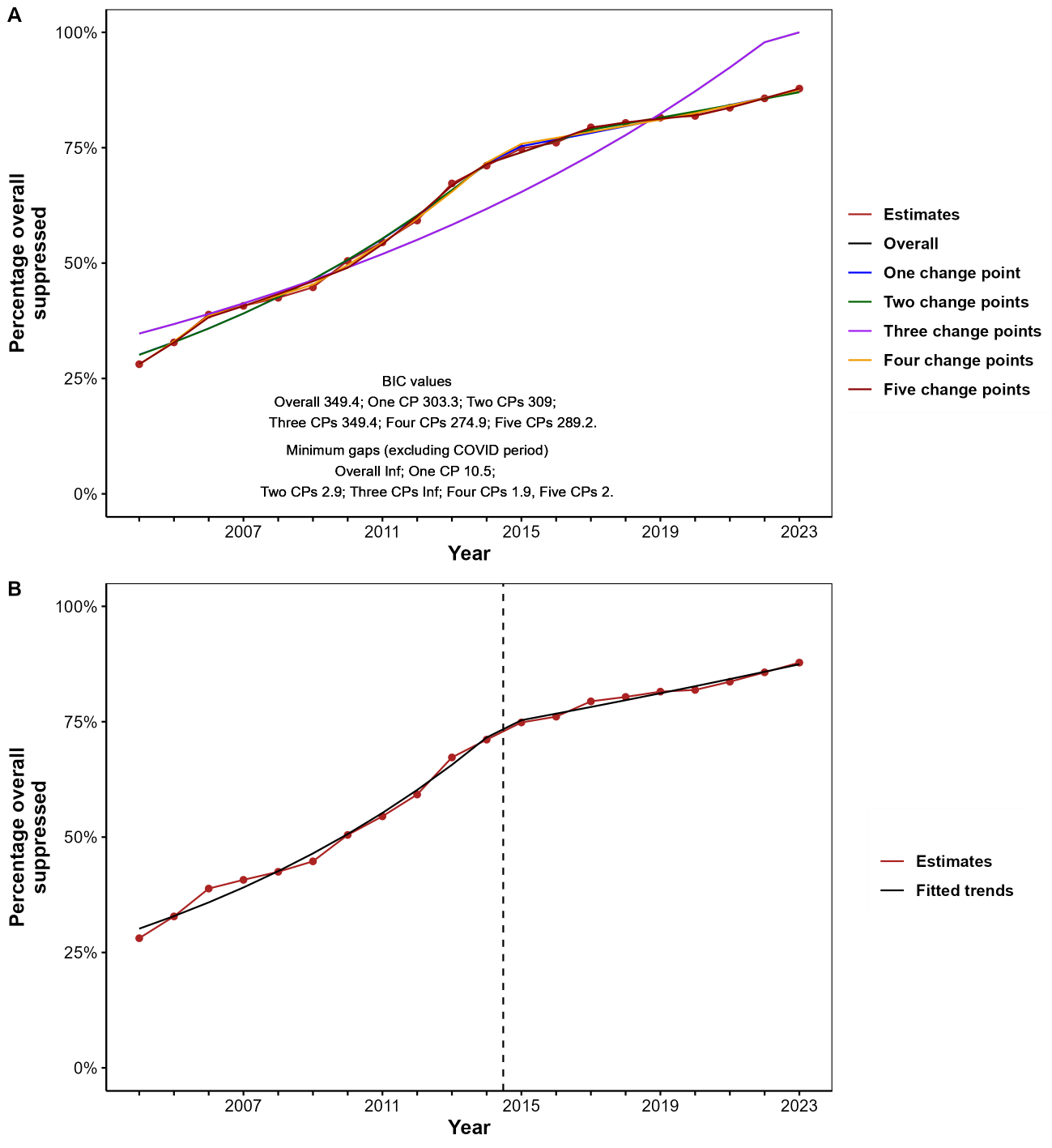


**Fitted models for the number of annual HIV notifications excluding people previously diagnosed overseas (A) and the best fitting three change point (CP) model (B) with the estimated change point (vertical dashed lines)**
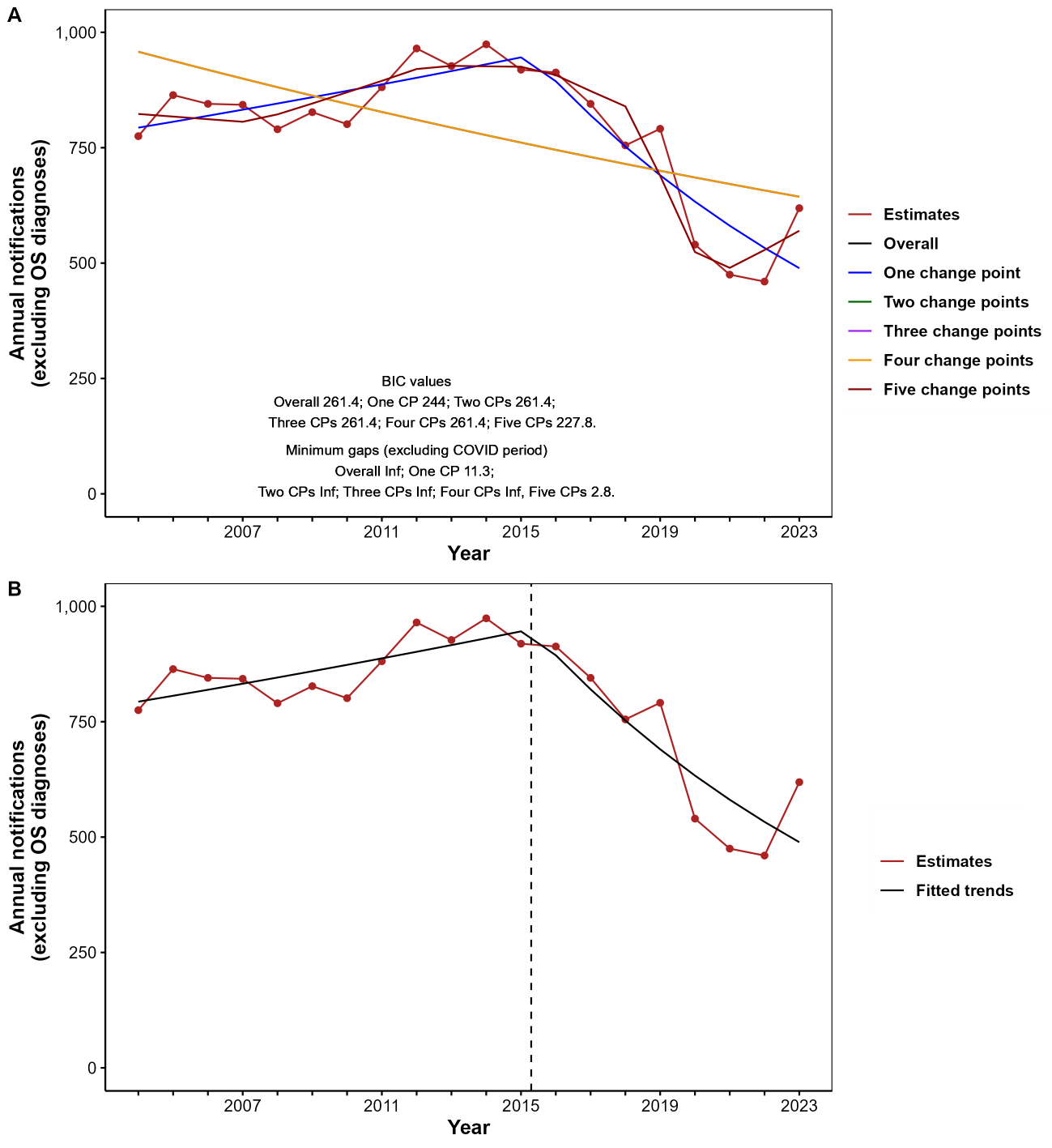


**Fitted models for the number of annual HIV notifications overall including people previously diagnosed overseas (OS) (A) and the best fitting three change point (CP) model (B) with the estimated change points (vertical dashed lines)**
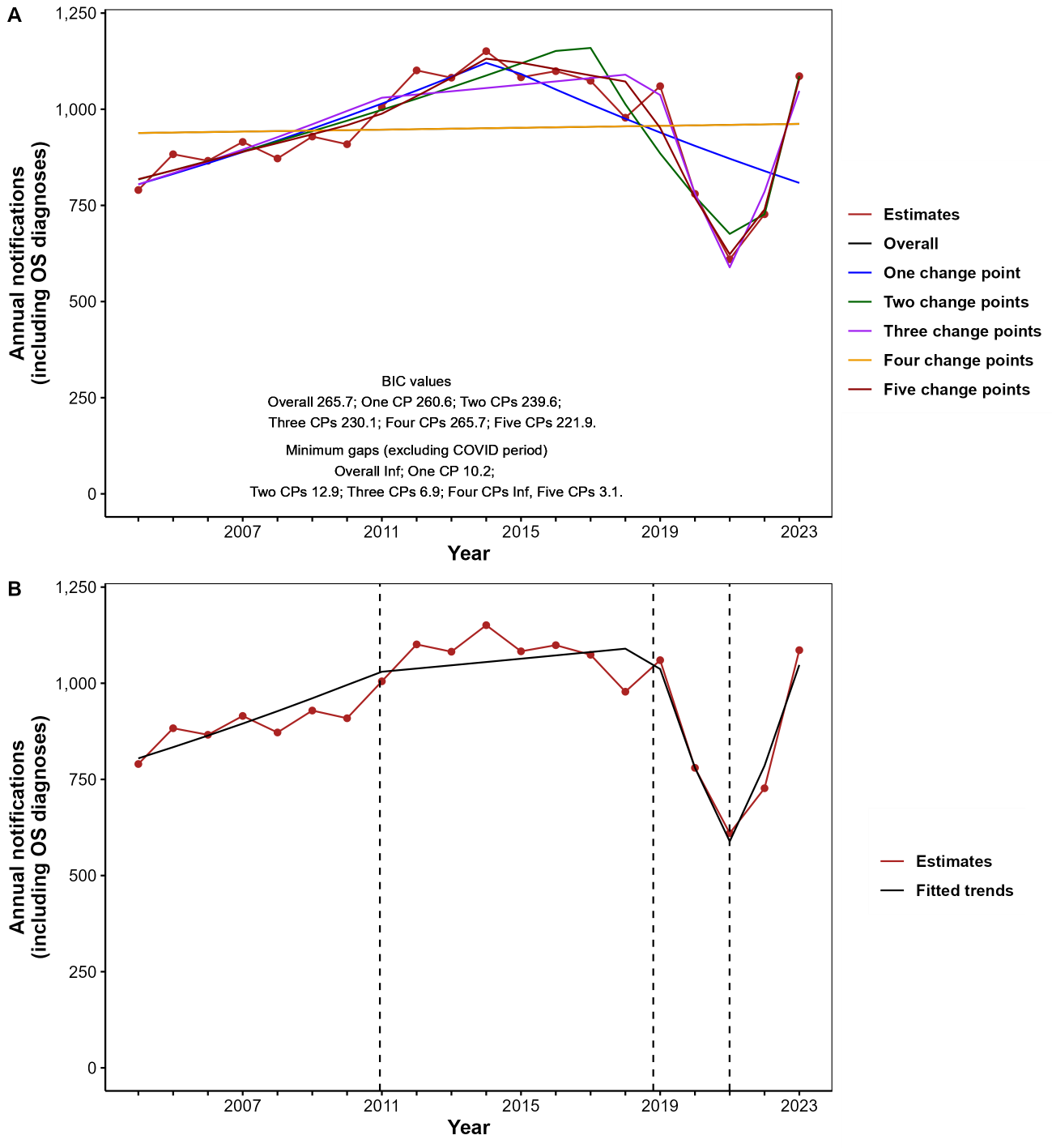


**Fitted models for the number of annual HIV notifications among people who have been previously diagnosed overseas (OS) (A) and the best fitting three change point (CP) model (B) with the estimated change points (vertical dashed lines)**
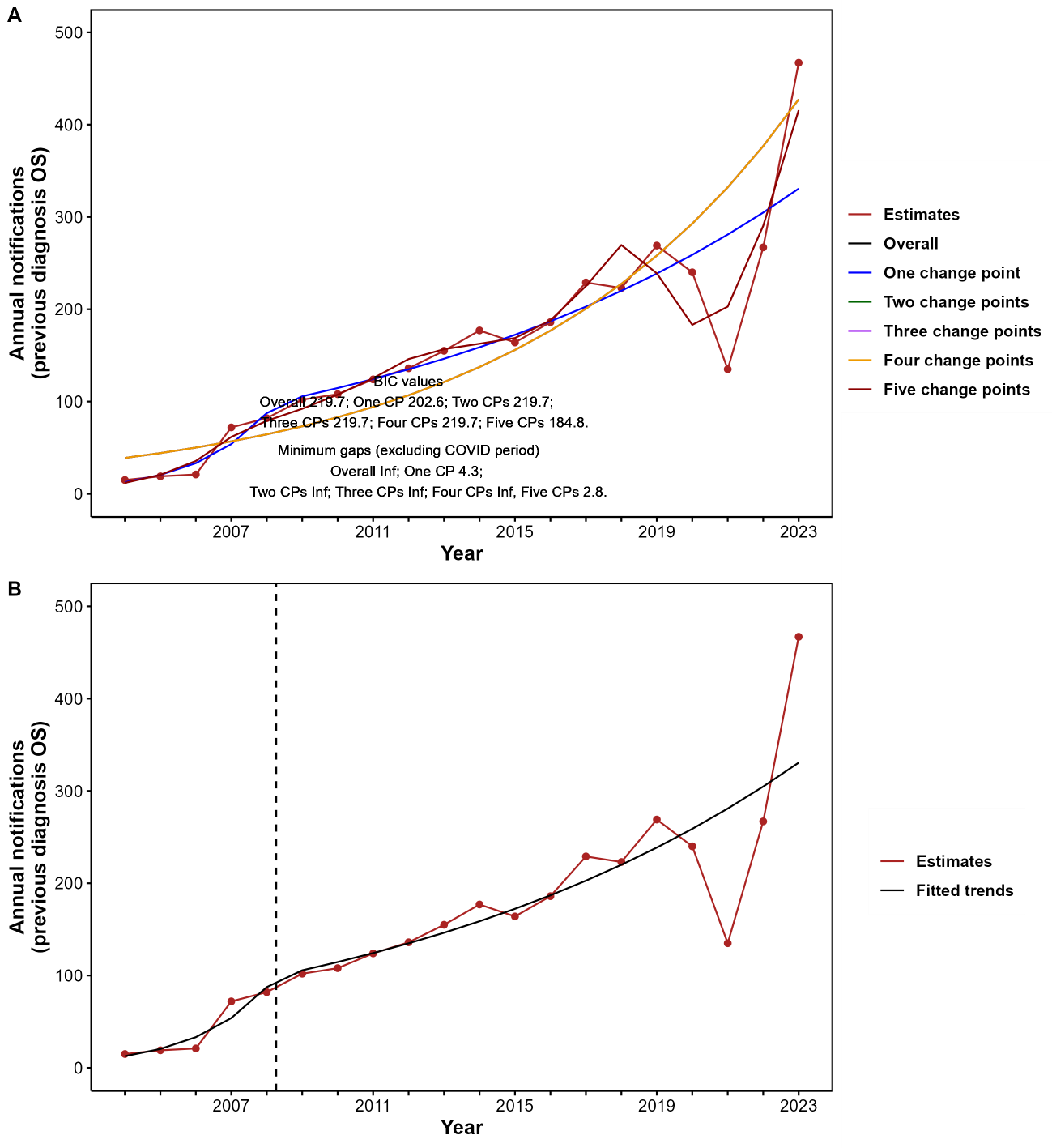


**Fitted models for the estimated number of annual new infections (A) and the best fitting two change point (CP) model (B) with the estimated change points (vertical dashed lines)**
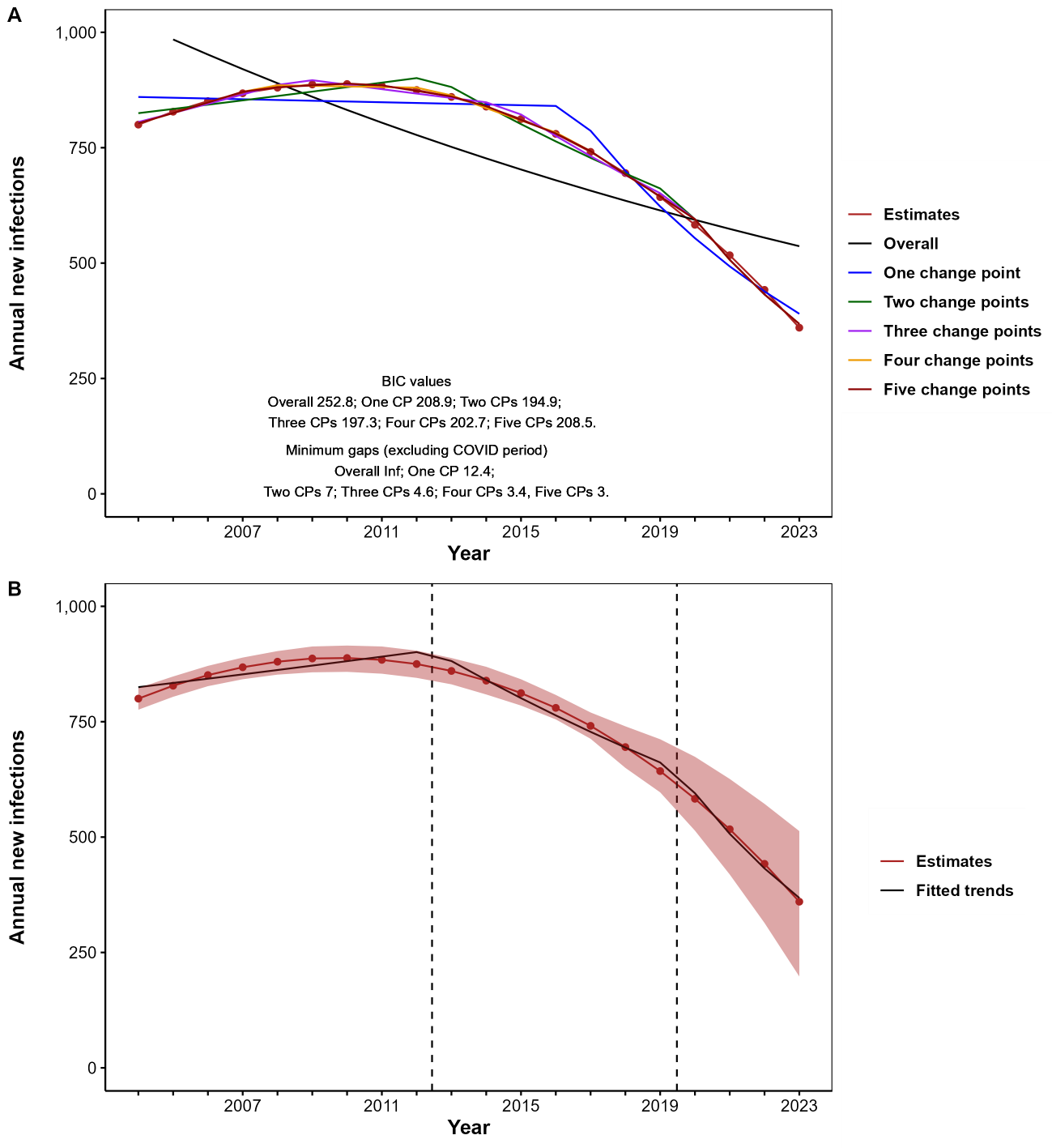


**Fitted models for the yearly diagnosed fraction (YDF) (A) and the best fitting two change point (CP) model (B) with the estimated change points (vertical dashed lines)**
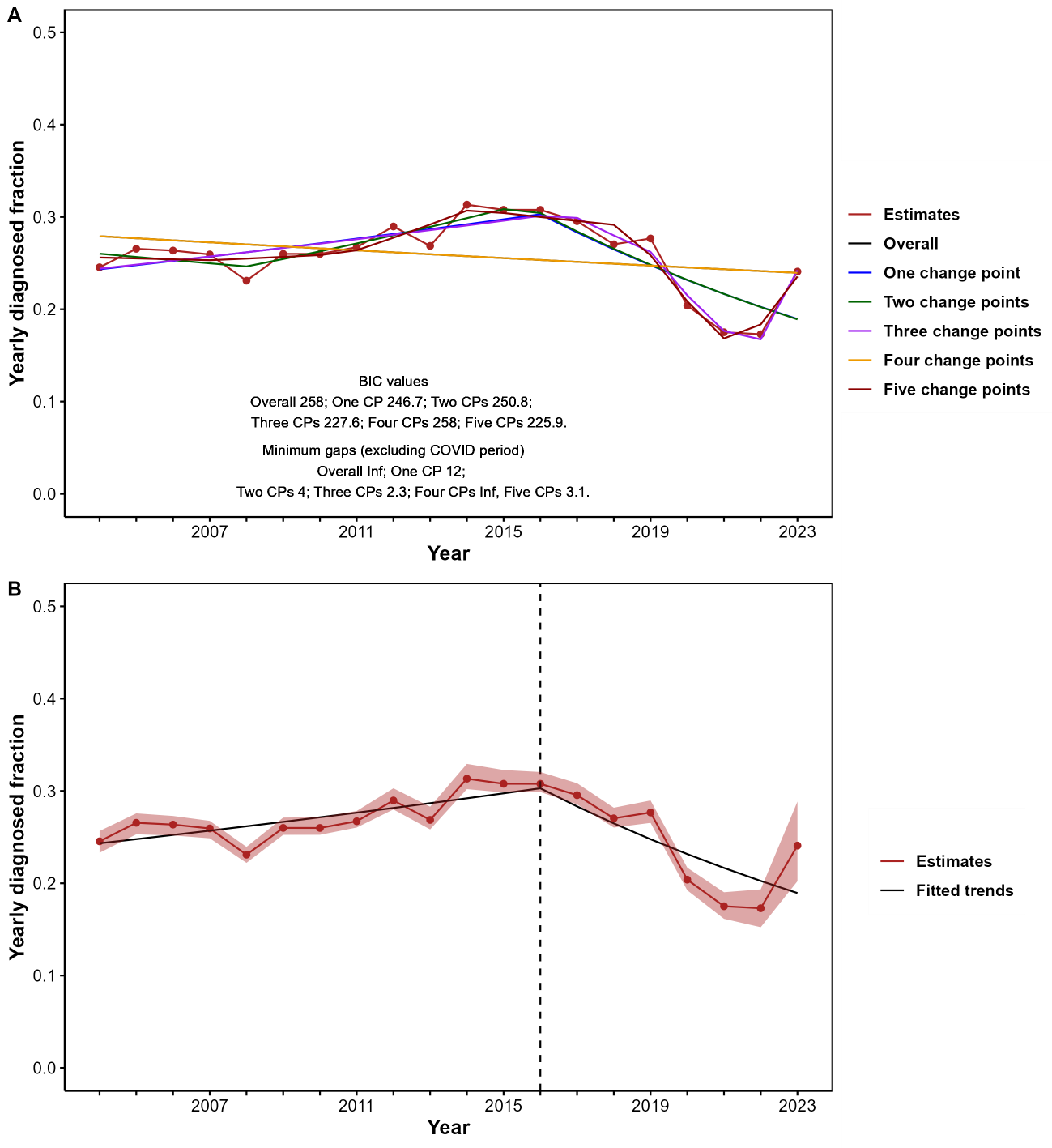


**Fitted models for the case detection rate (CDR) (A) and the best fitting two change point (CP) model (B) with the estimated change points (vertical dashed lines)**
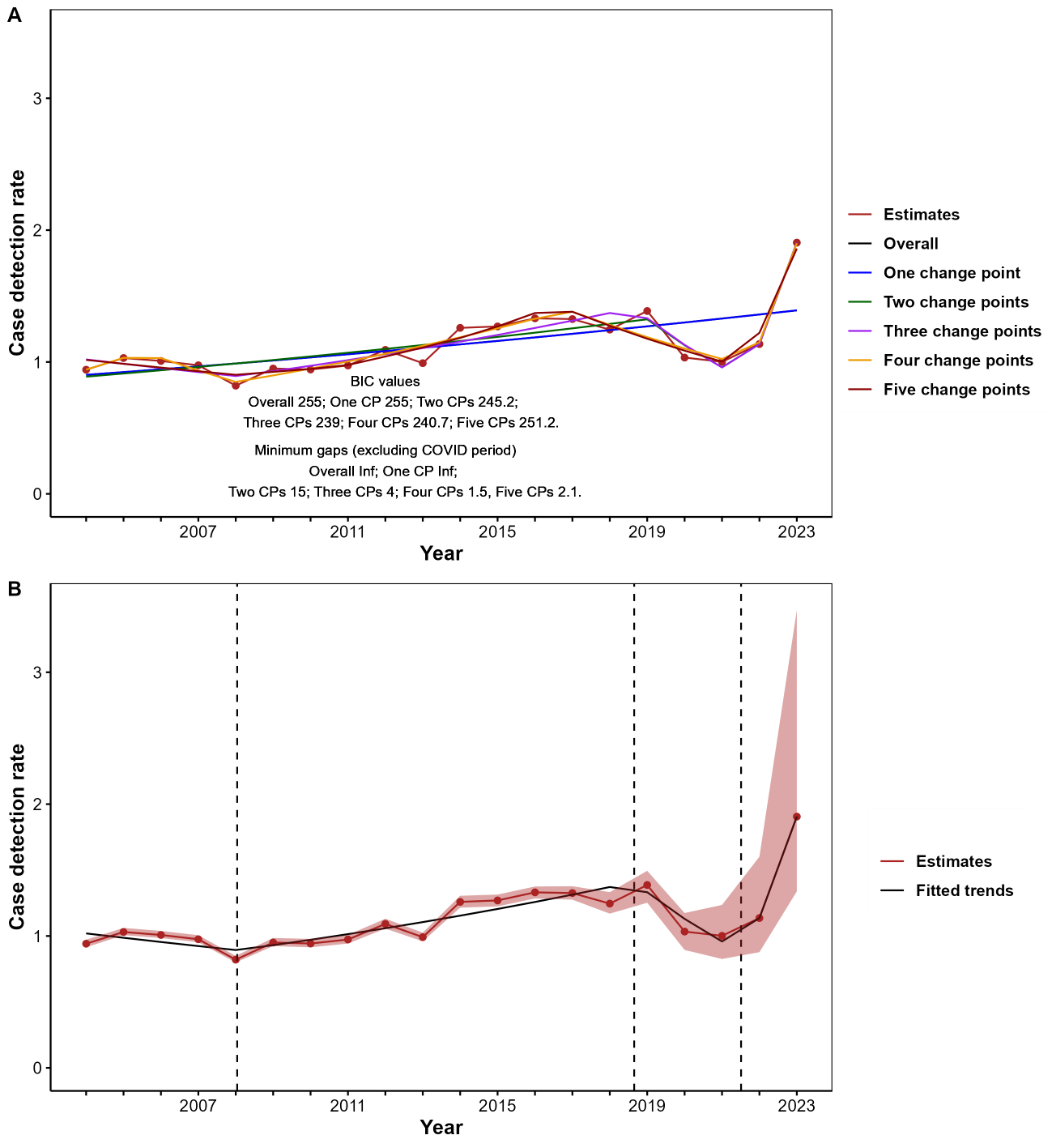


**Fitted models for the incidence prevalence ratio (IPR) (A) and the best fitting two change point (CP) model (B) with the estimated change points (vertical dashed lines)**
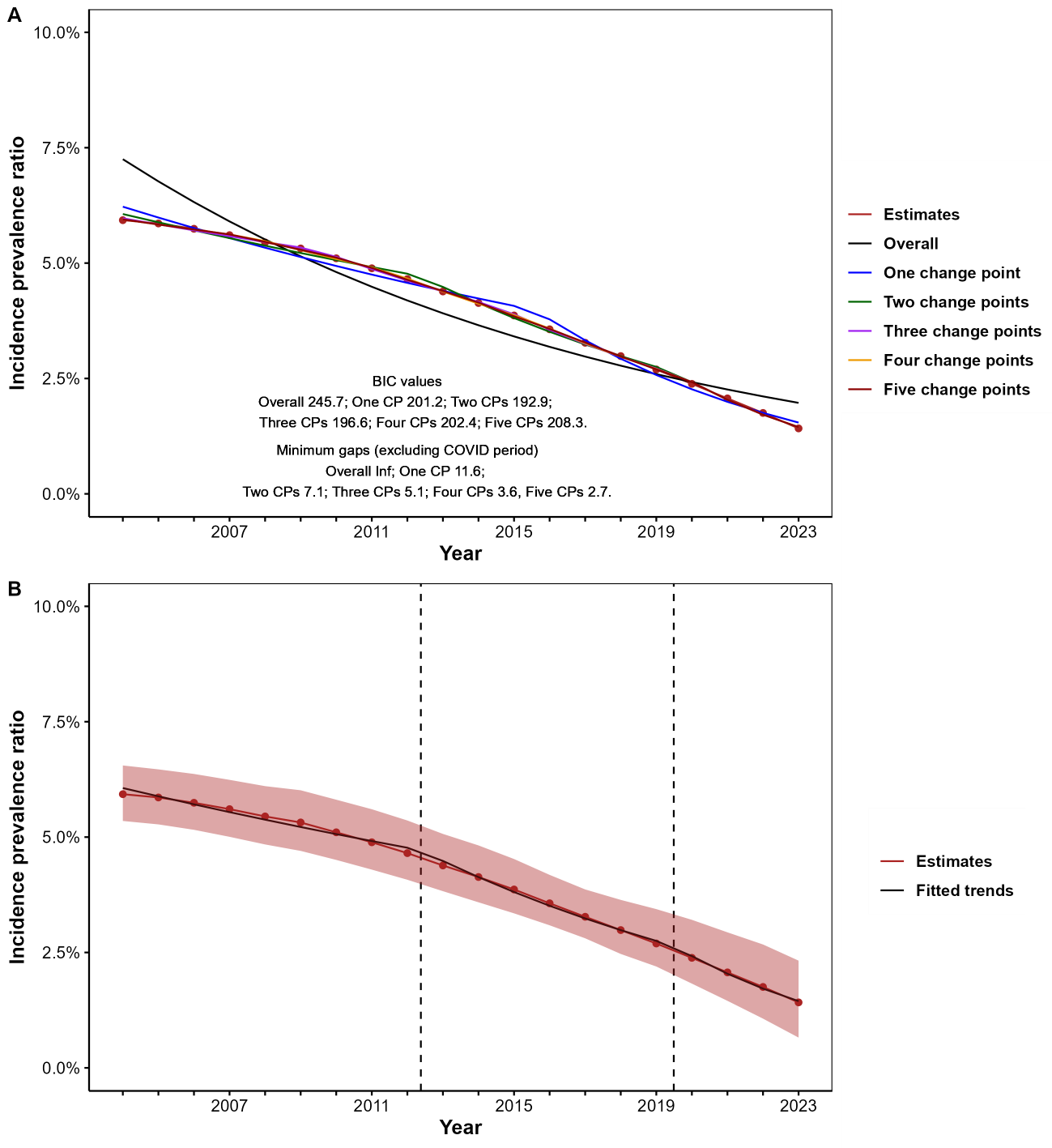


**Fitted models for the incidence mortality ratio (IMR) (A) and the best fitting one change point (CP) model (B) with the estimated change point (vertical dashed line)**
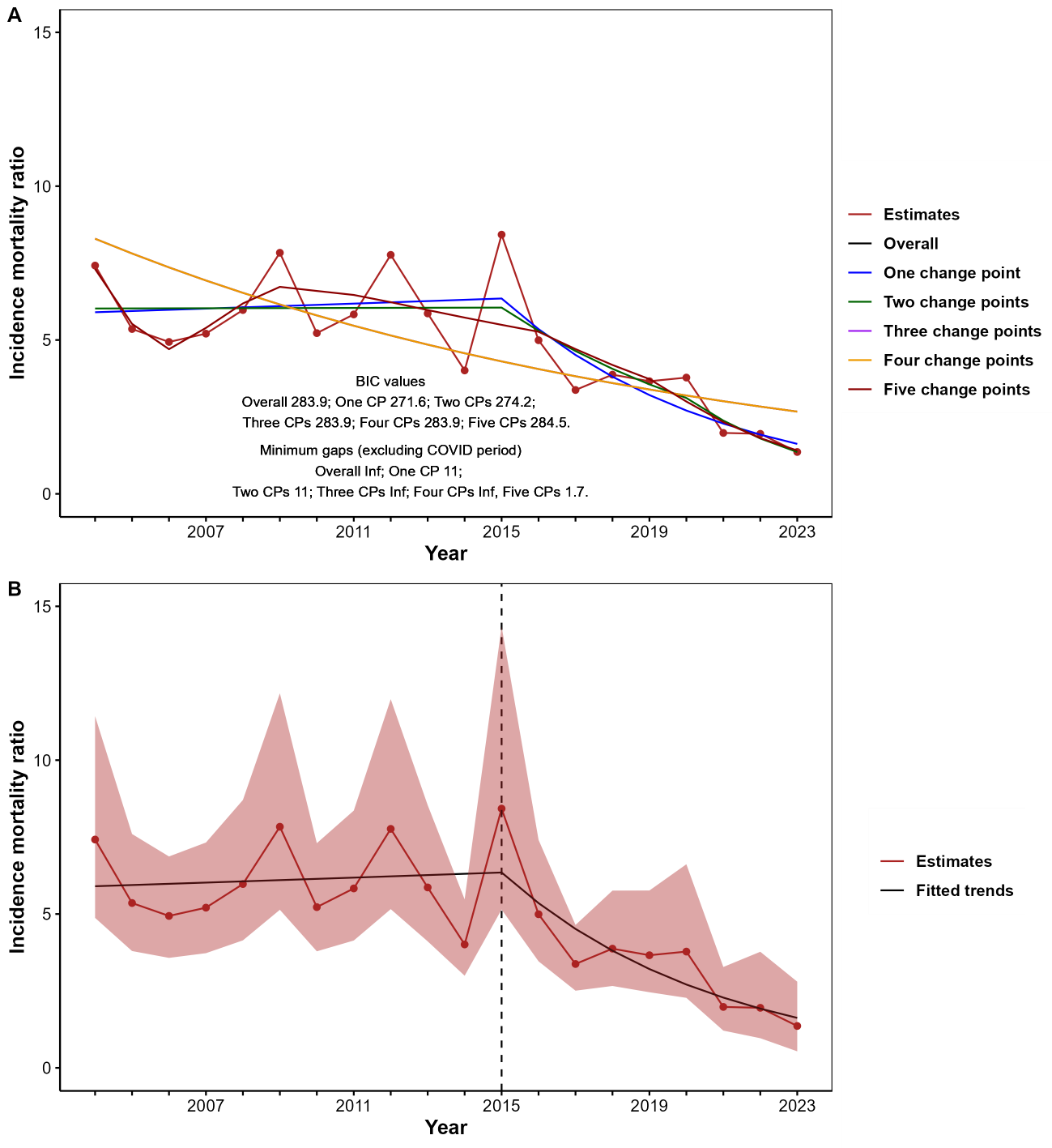


## Figures for females

**Fitted models for the number of people living with HIV (A) and the best fitting two change point (CP) model with estimated change points (vertical dashed lines)**
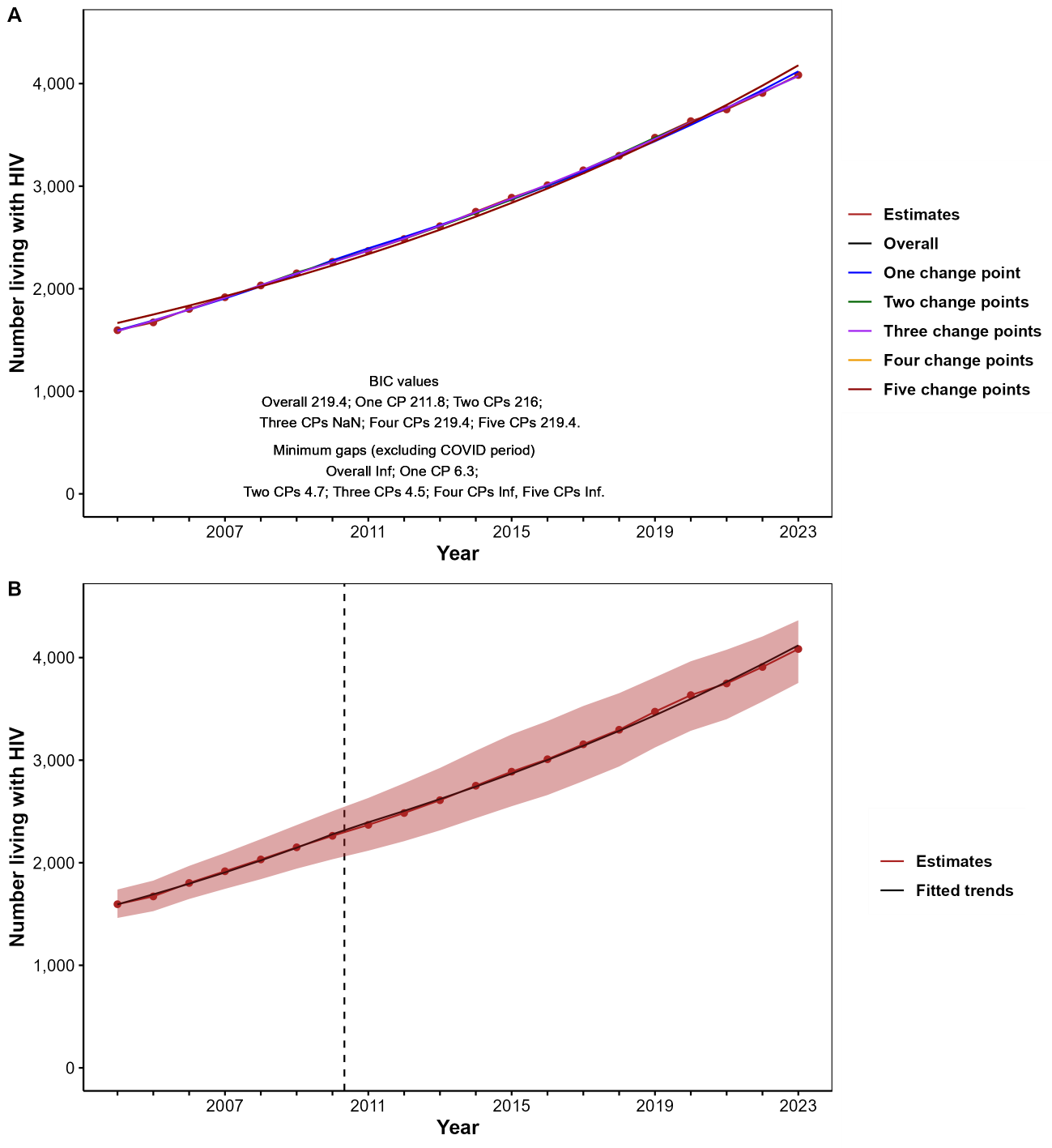


**Fitted models for the number of people living with HIV (A) and the best fitting two change point model (B) with estimated change points (vertical dashed lines)**
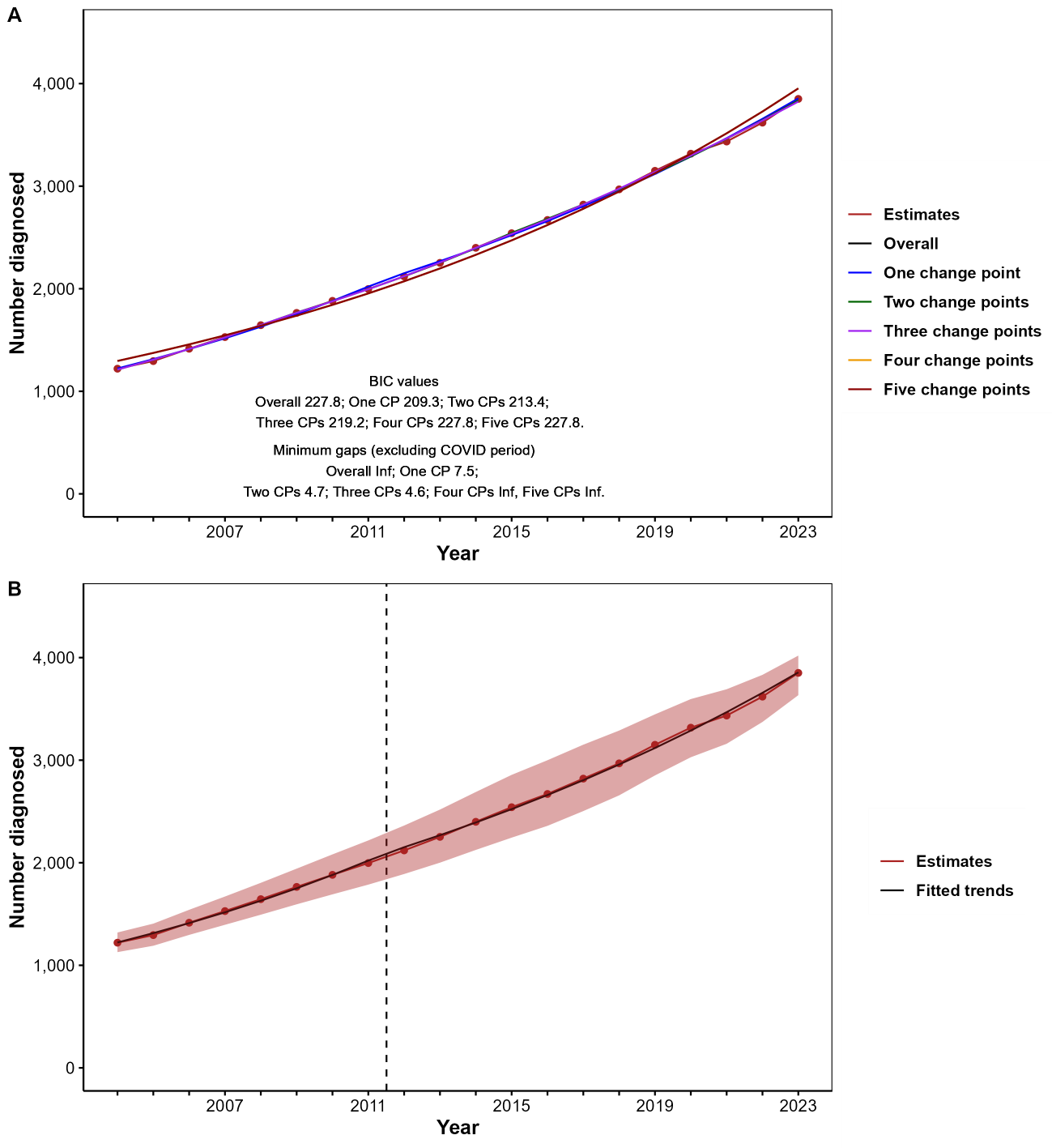


**Fitted models for the number of people living with diagnosed HIV (A) and the best fitting two change point model (B) with estimated change points (vertical dashed lines)**

**Fitted models for the number of people living with HIV on ART (A) and the best fitting two change point model (B) with estimated change points (vertical dashed lines)**
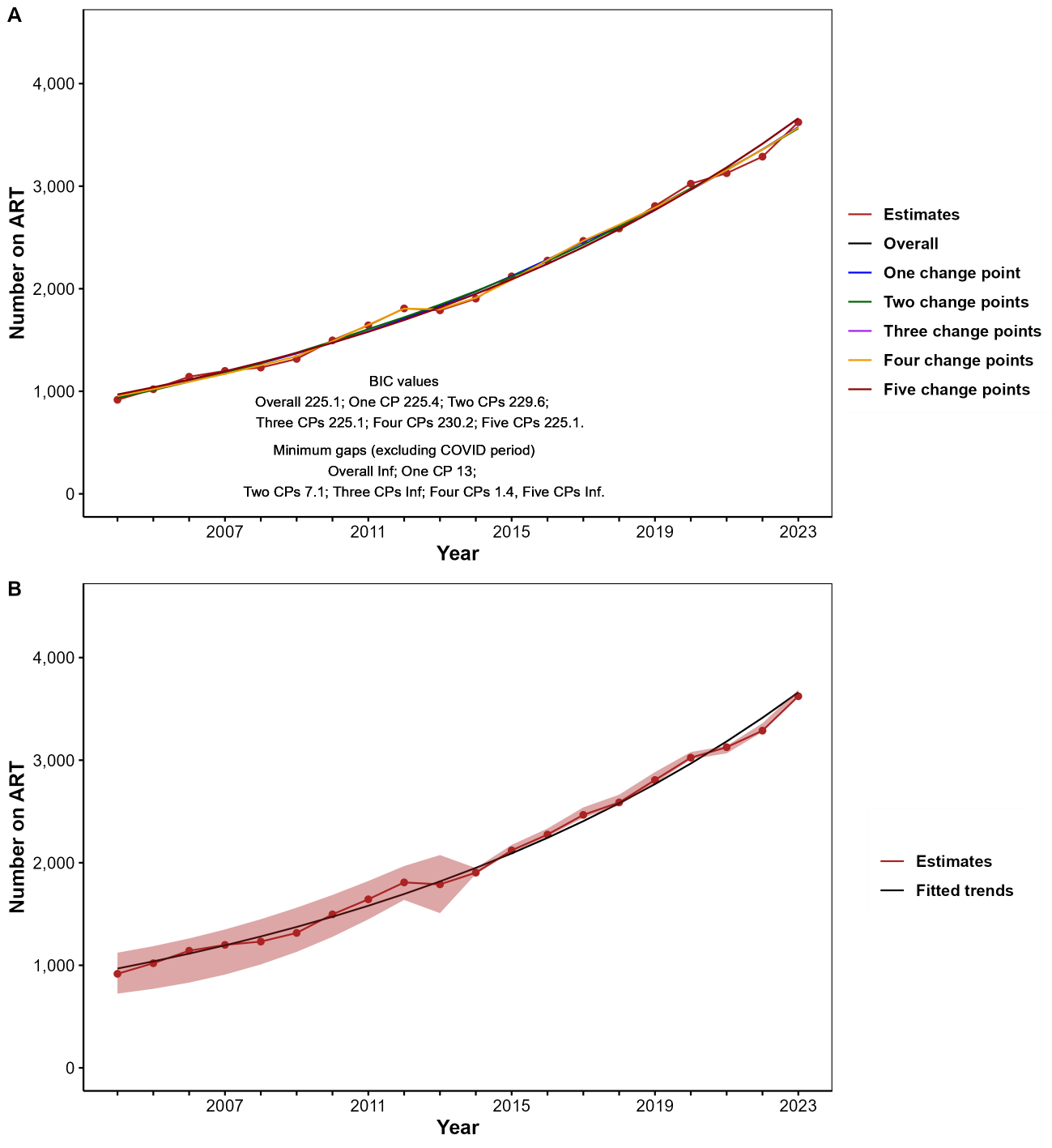


**Fitted models for the number of people with a suppressed viral load (A) and the best fitting one change point model (B) with estimated change points (vertical dashed lines)**
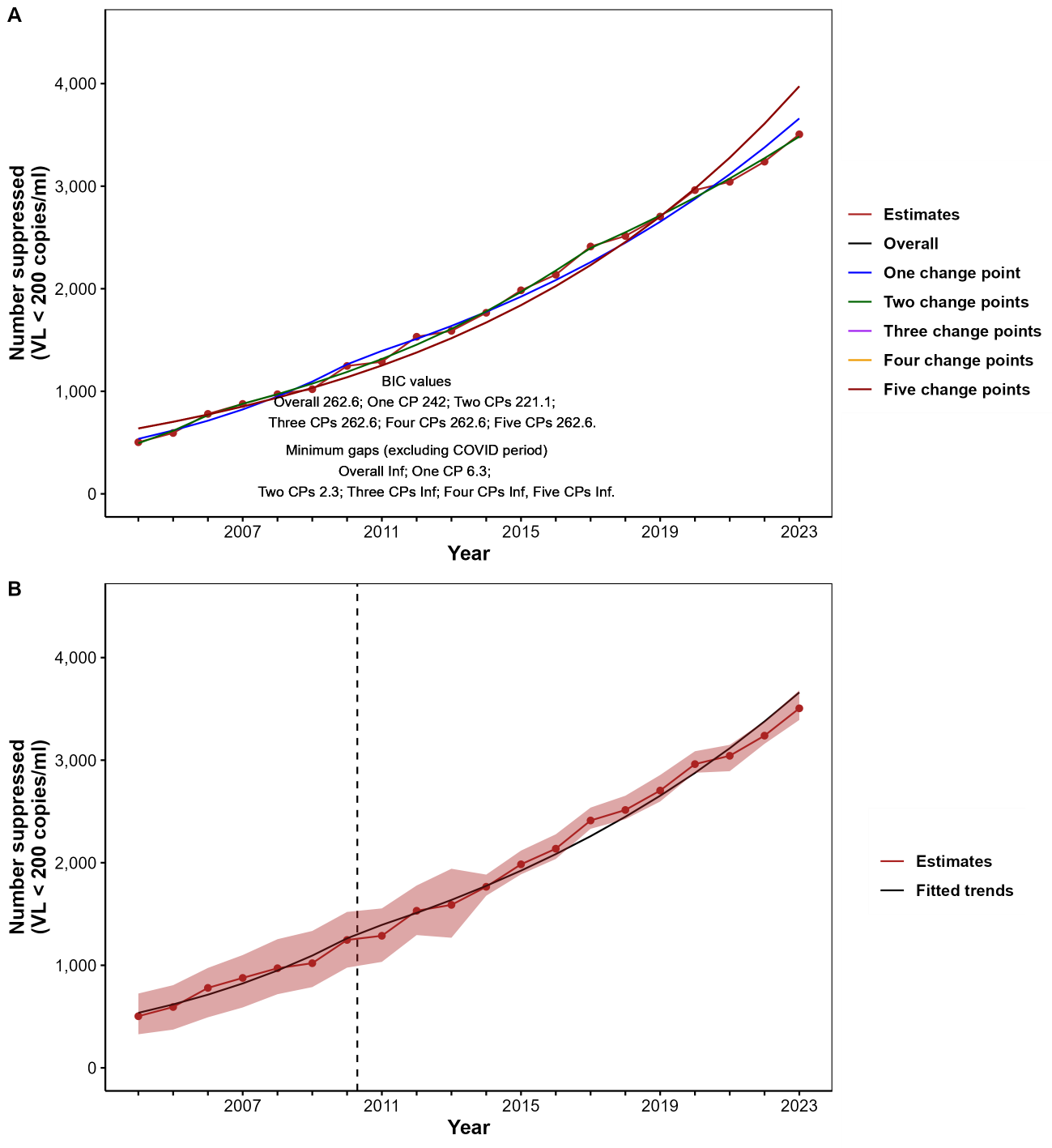


**Fitted models for the number of people living with undiagnosed HIV (A) and the best fitting two change point model (B) with estimated change points (vertical dashed lines)**
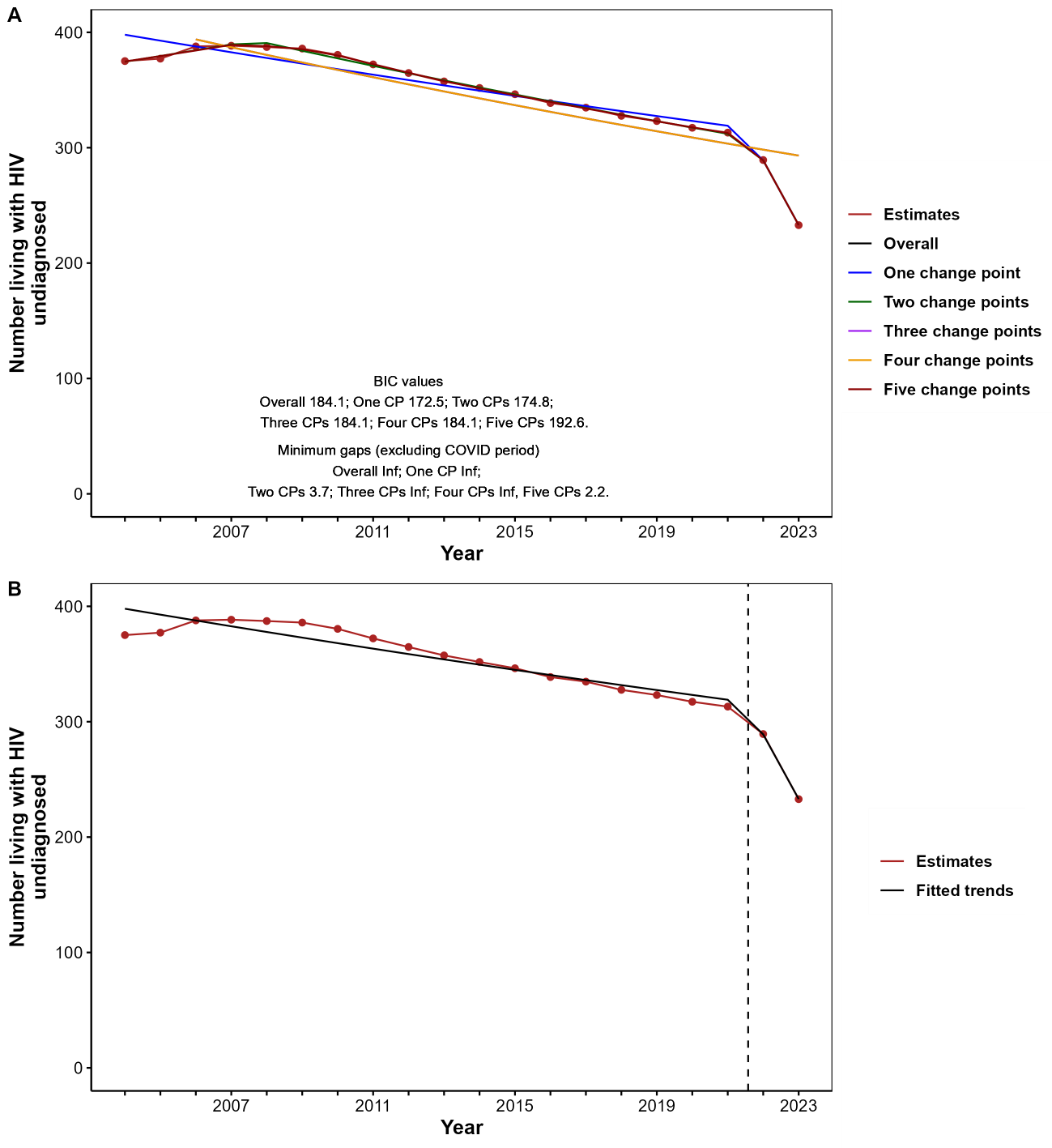


**Fitted models for the number of people diagnosed with HIV but not on ART (A) and the best fitting three change point model with estimated change points (vertical dashed lines)**
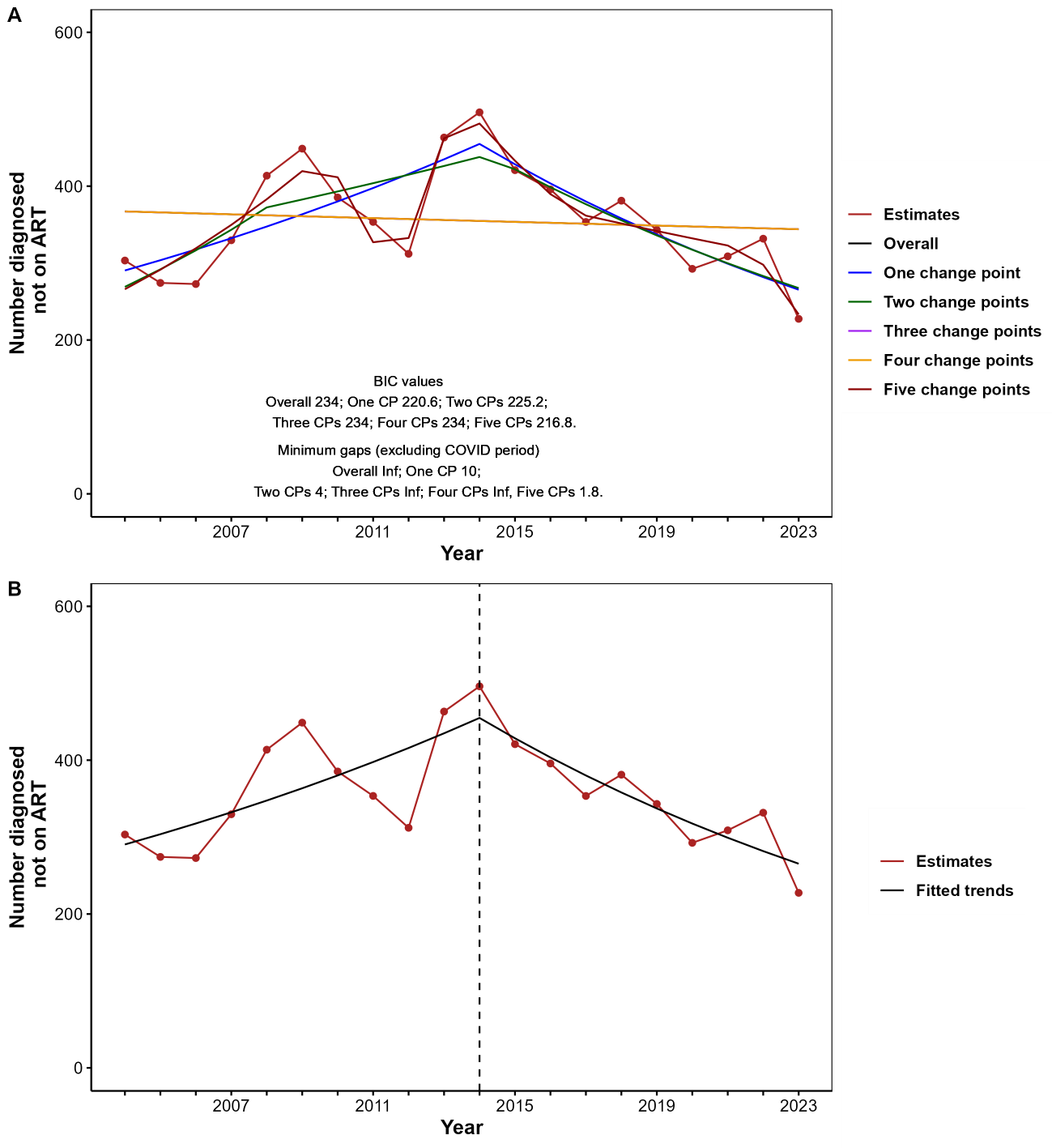


**Fitted models for the number of people on ART but with an unsuppressed viral load (A) and the best fitting overall model (B)**
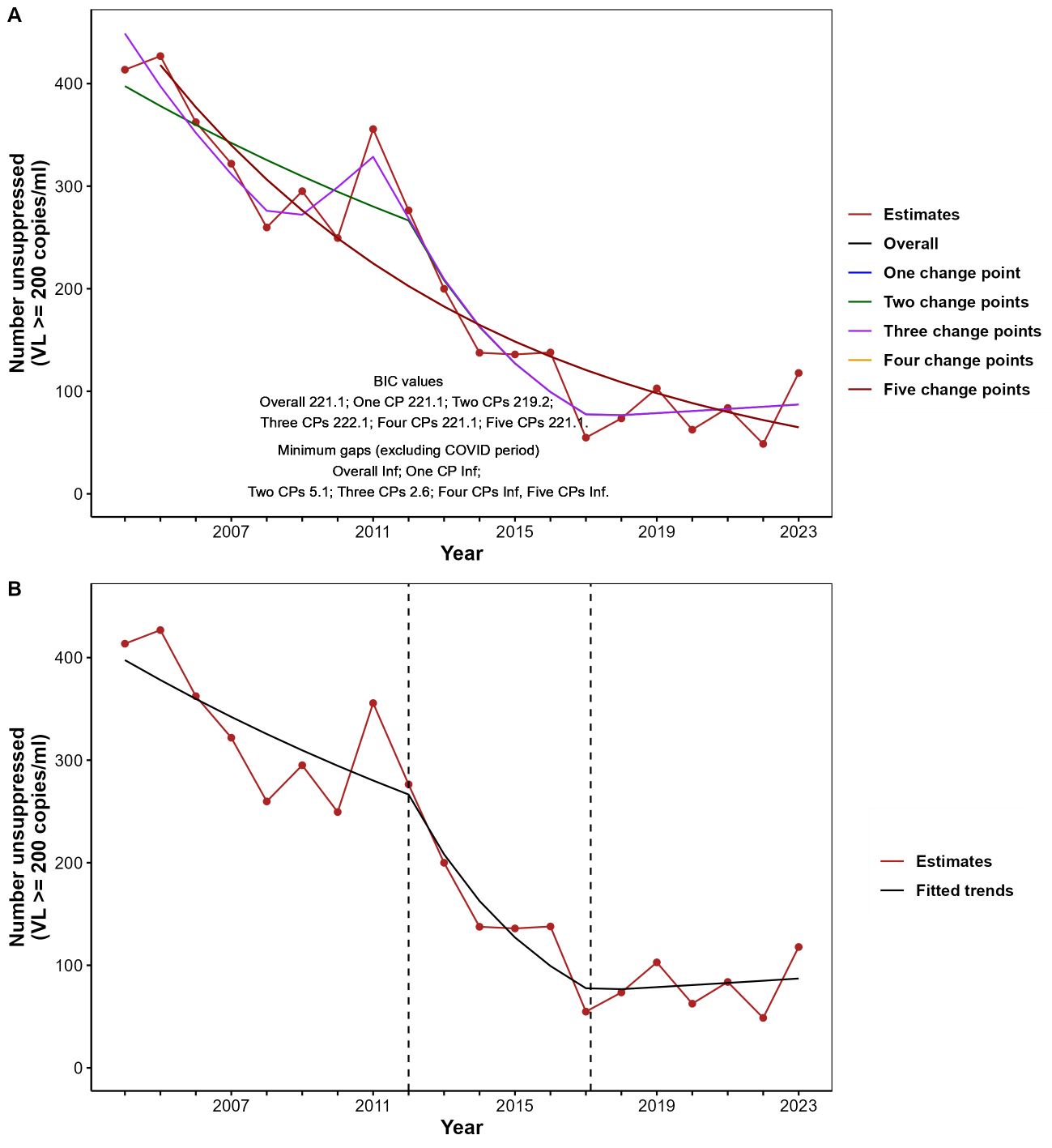


**Fitted models for the percentage of people living with HIV diagnosed (A) and the best fitting one change point model (B) with the estimated change point (vertical dashed line)**
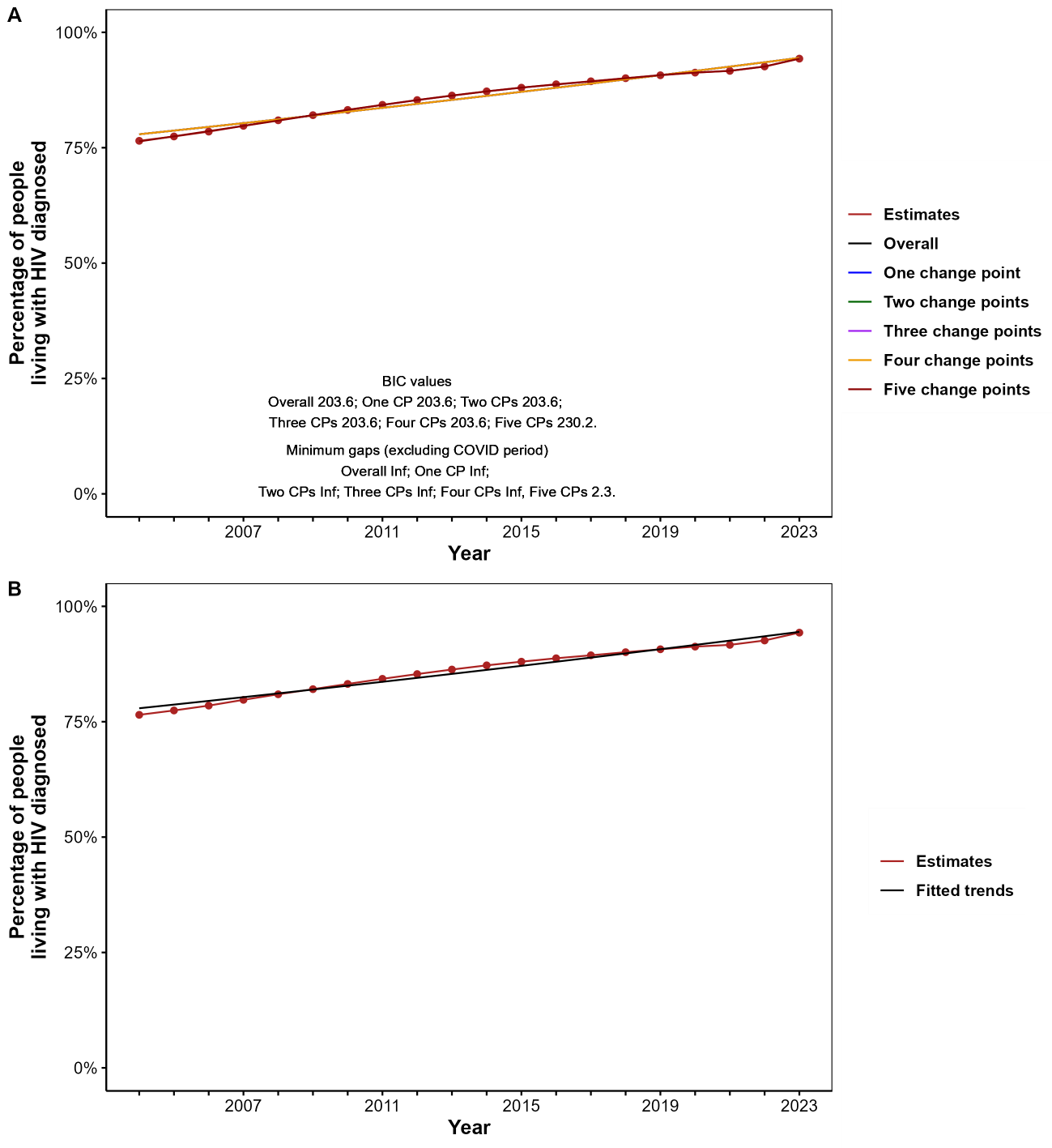


**Fitted models for the percentage of people diagnosed with HIV on ART (A) and the best fitting two change point model (B) with the estimated change points (vertical dashed lines)**
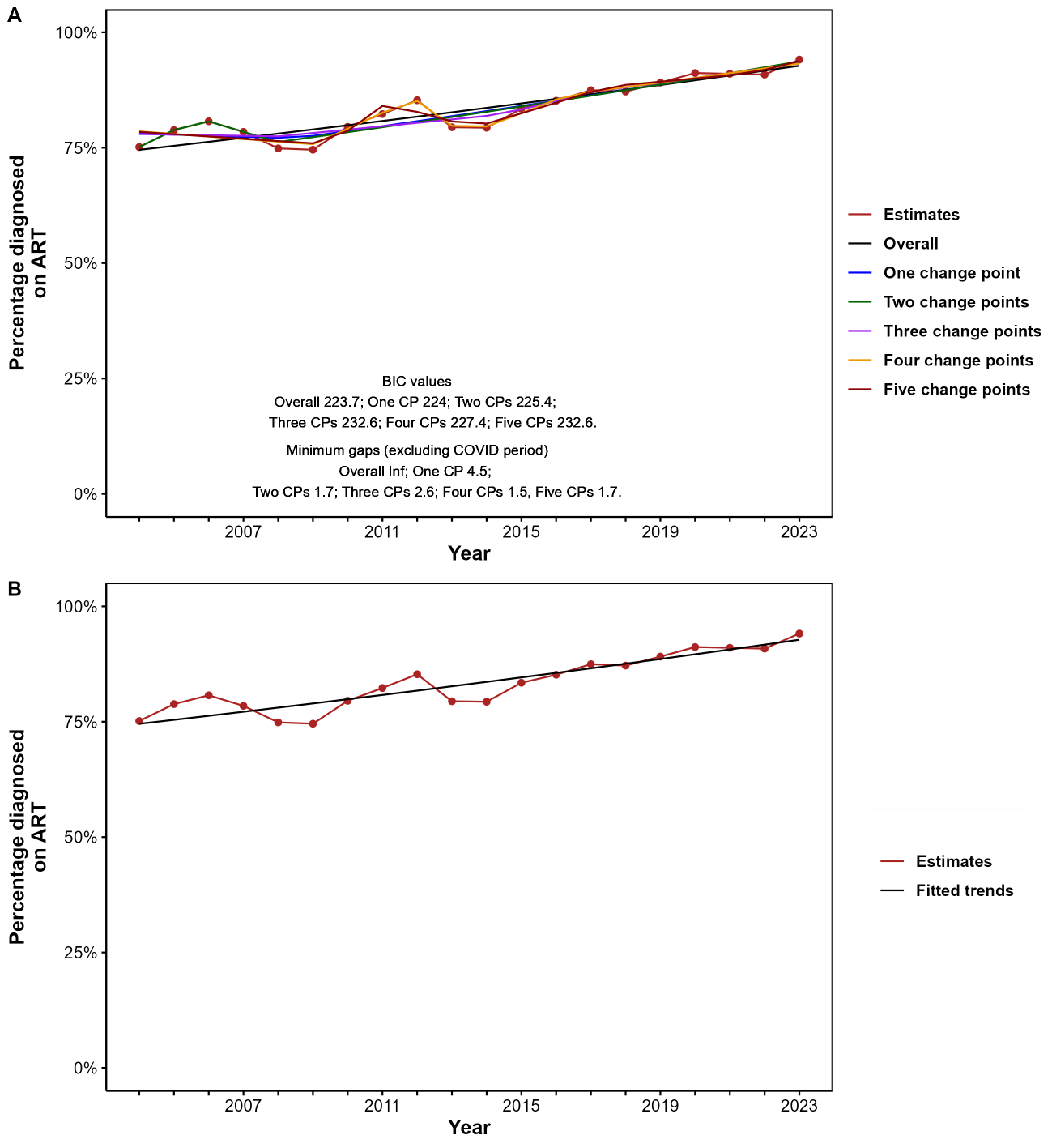


**Fitted models for the percentage of on ART with a suppressed viral load (A) and the best fitting one change point (CP) model (B) with the estimated change point (vertical dashed line)**
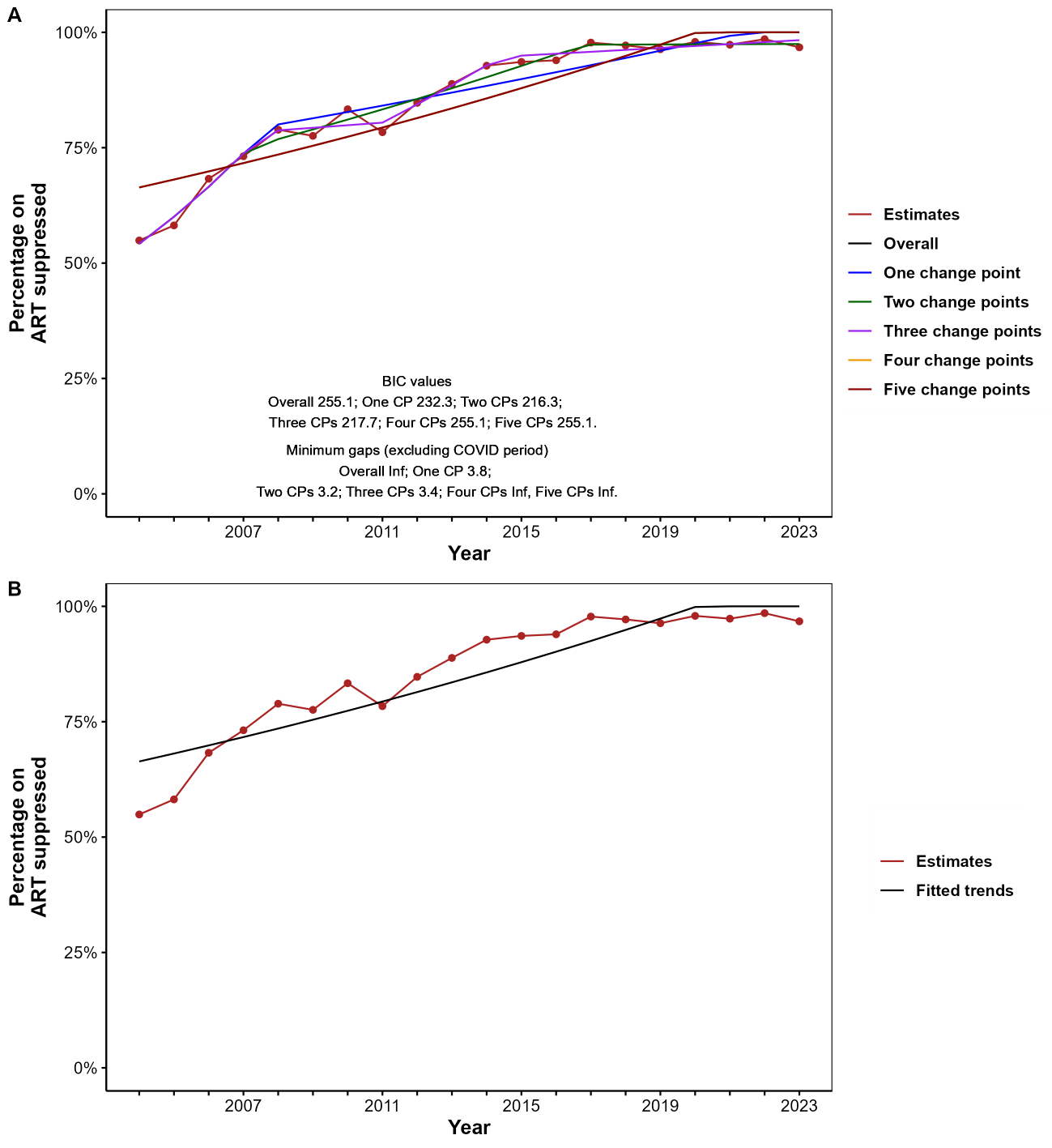


**Fitted models for the percentage of all people living with HIV with a suppressed viral load (A) and the best fitting one change point (CP) model (B) with the estimated change points (vertical dashed line)**
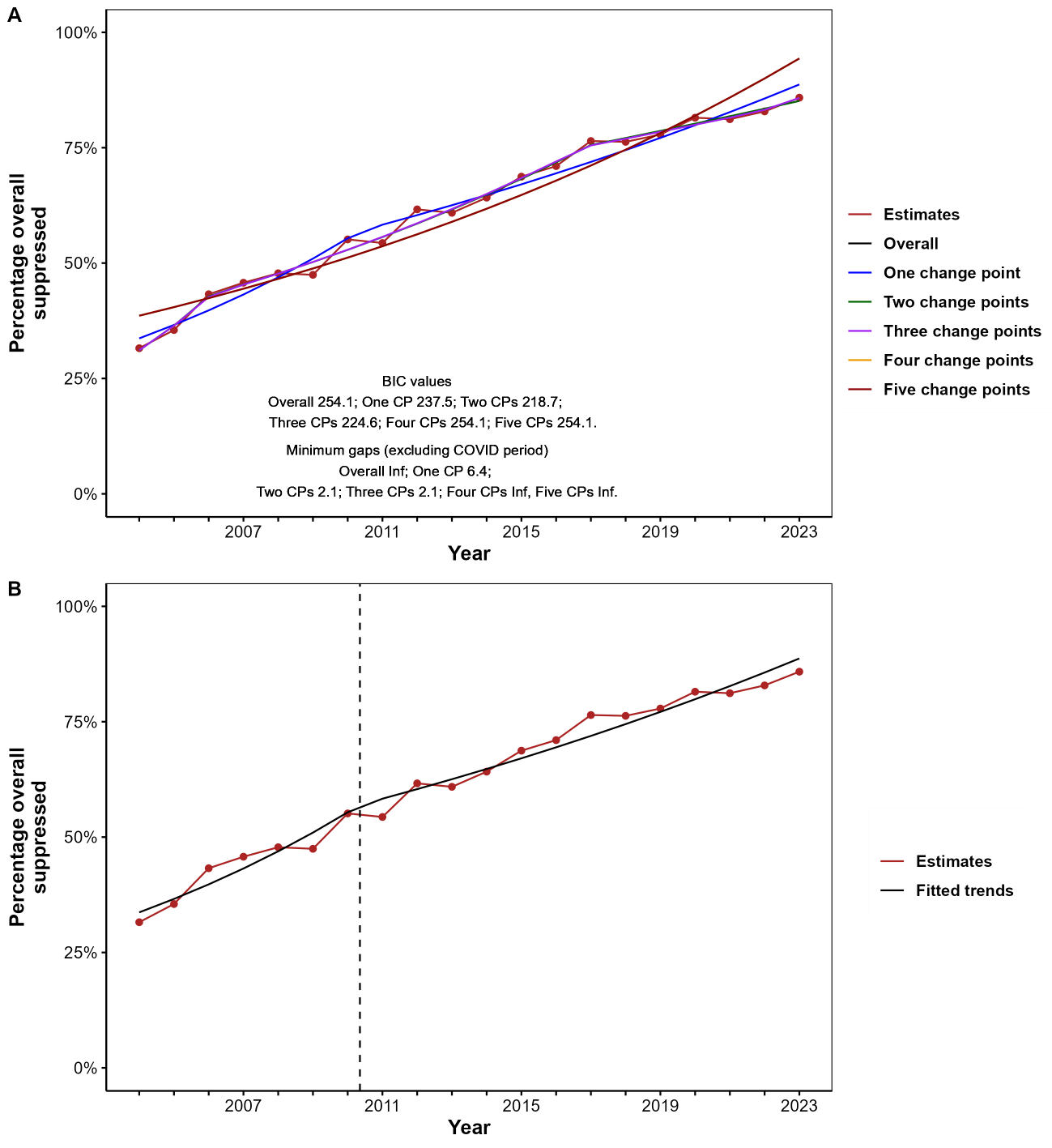


**Fitted models for the number of annual HIV notifications excluding people previously diagnosed overseas (A) and the best fitting three change point (CP) model (B) with the estimated change point (vertical dashed lines)**
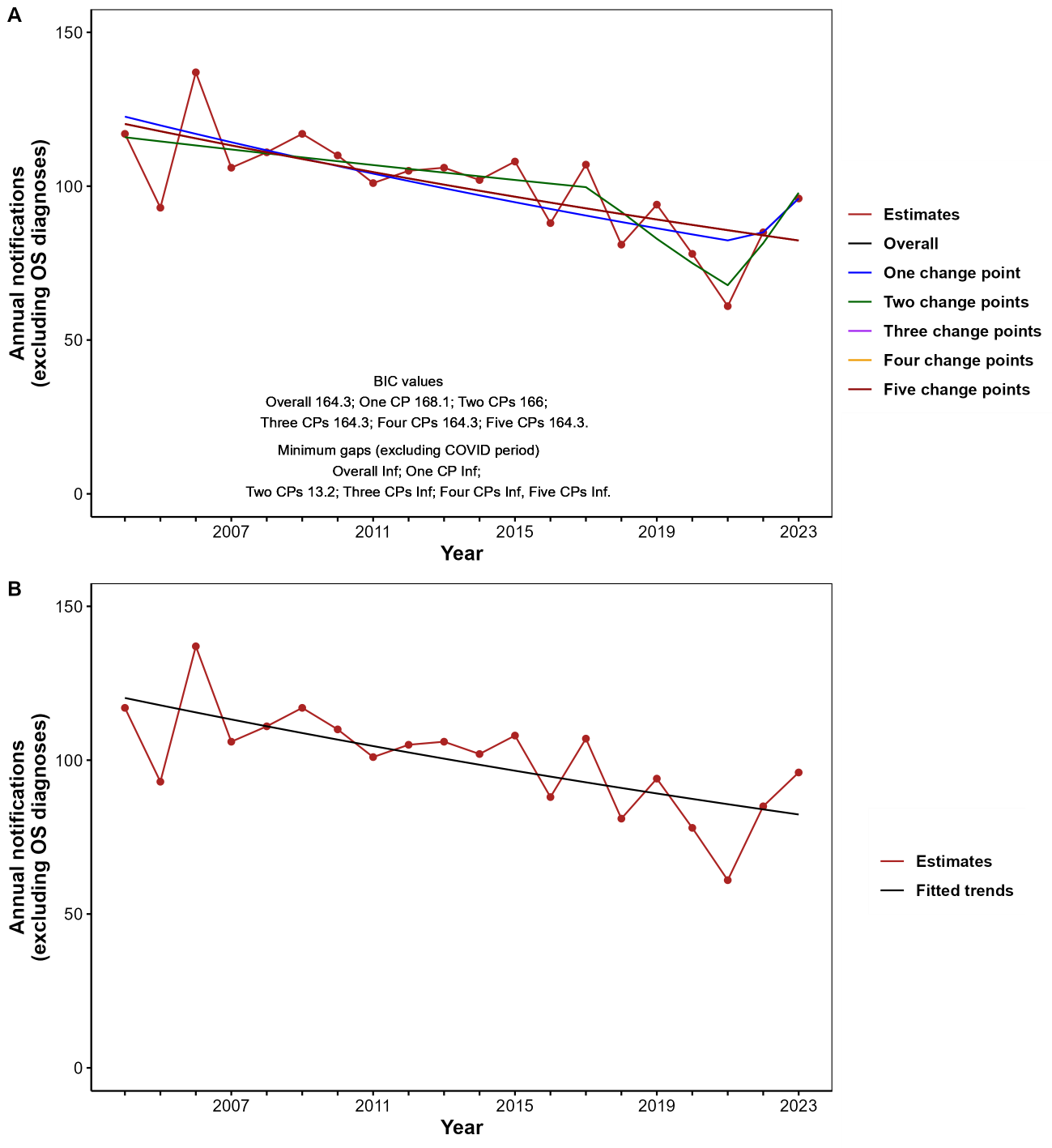


**Fitted models for the number of annual HIV notifications overall including people previously diagnosed overseas (OS) (A) and the best fitting three change point (CP) model (B) with the estimated change points (vertical dashed lines)**
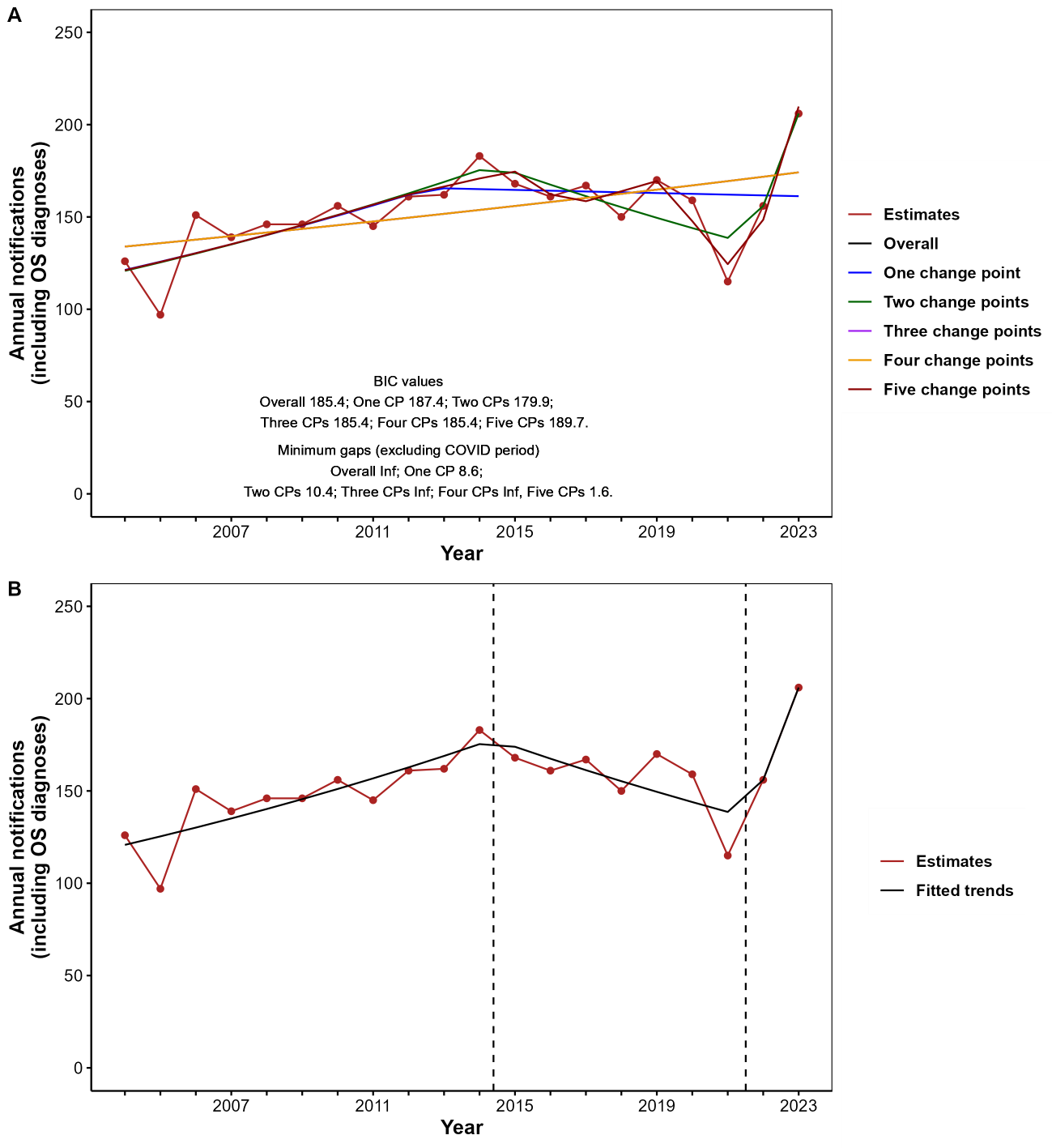


**Fitted models for the number of annual HIV notifications among people who have been previously diagnosed overseas (OS) (A) and the best fitting three change point (CP) model (B) with the estimated change points (vertical dashed lines)**
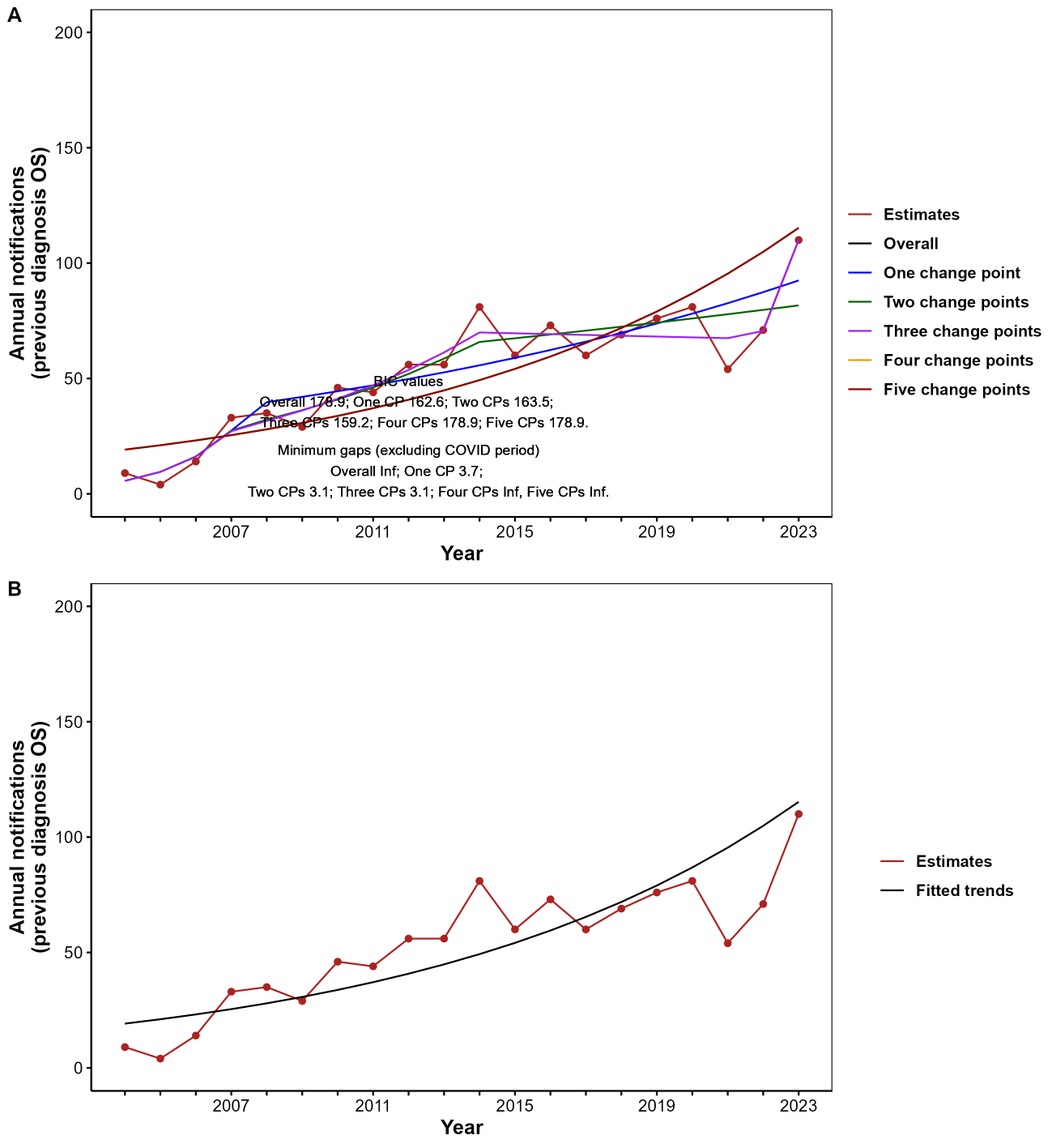


**Fitted models for the estimated number of annual new infections (A) and the best fitting two change point (CP) model (B) with the estimated change points (vertical dashed lines)**
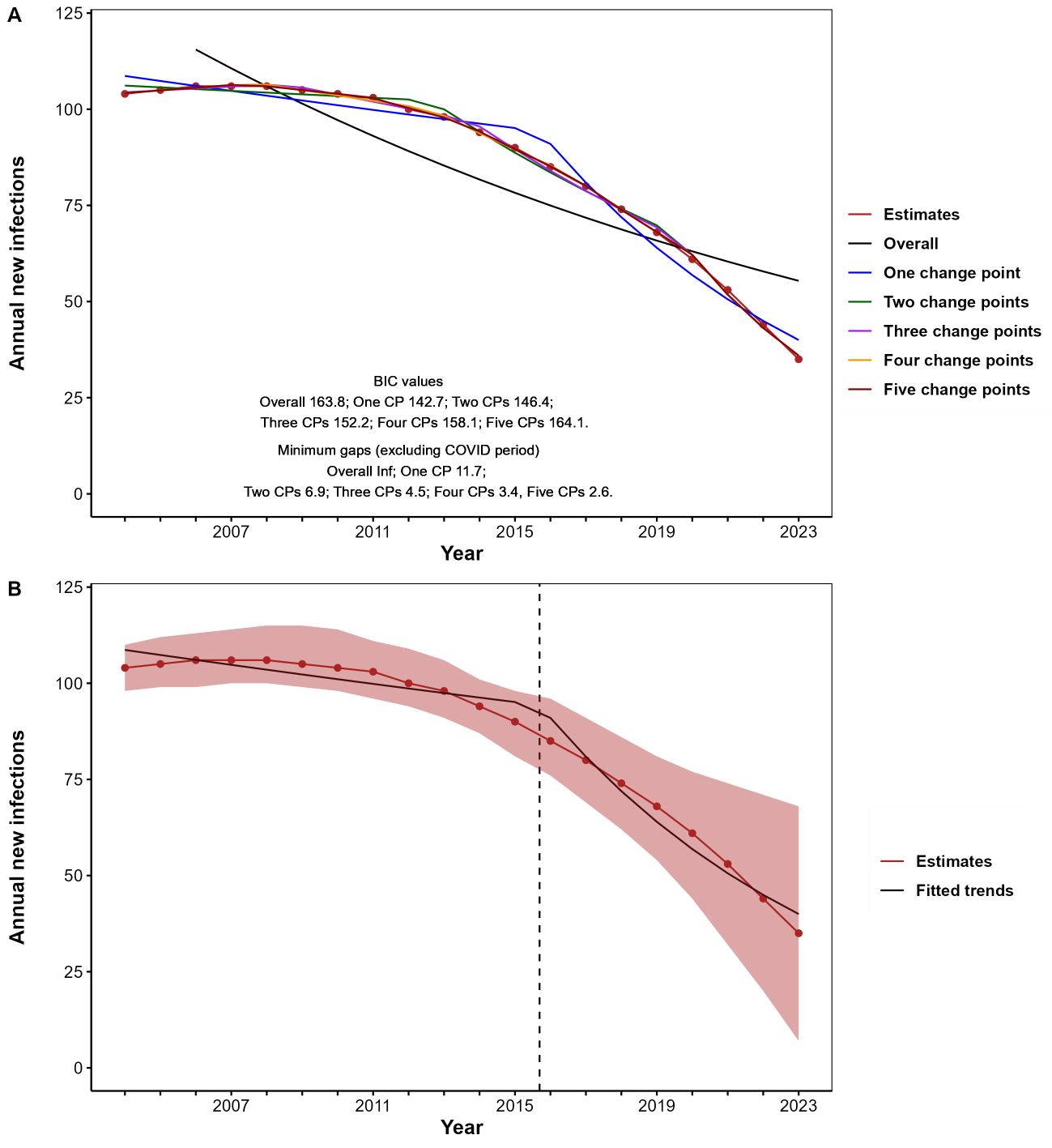


**Fitted models for the yearly diagnosed fraction (YDF) (A) and the best fitting two change point (CP) model (B) with the estimated change points (vertical dashed lines)**
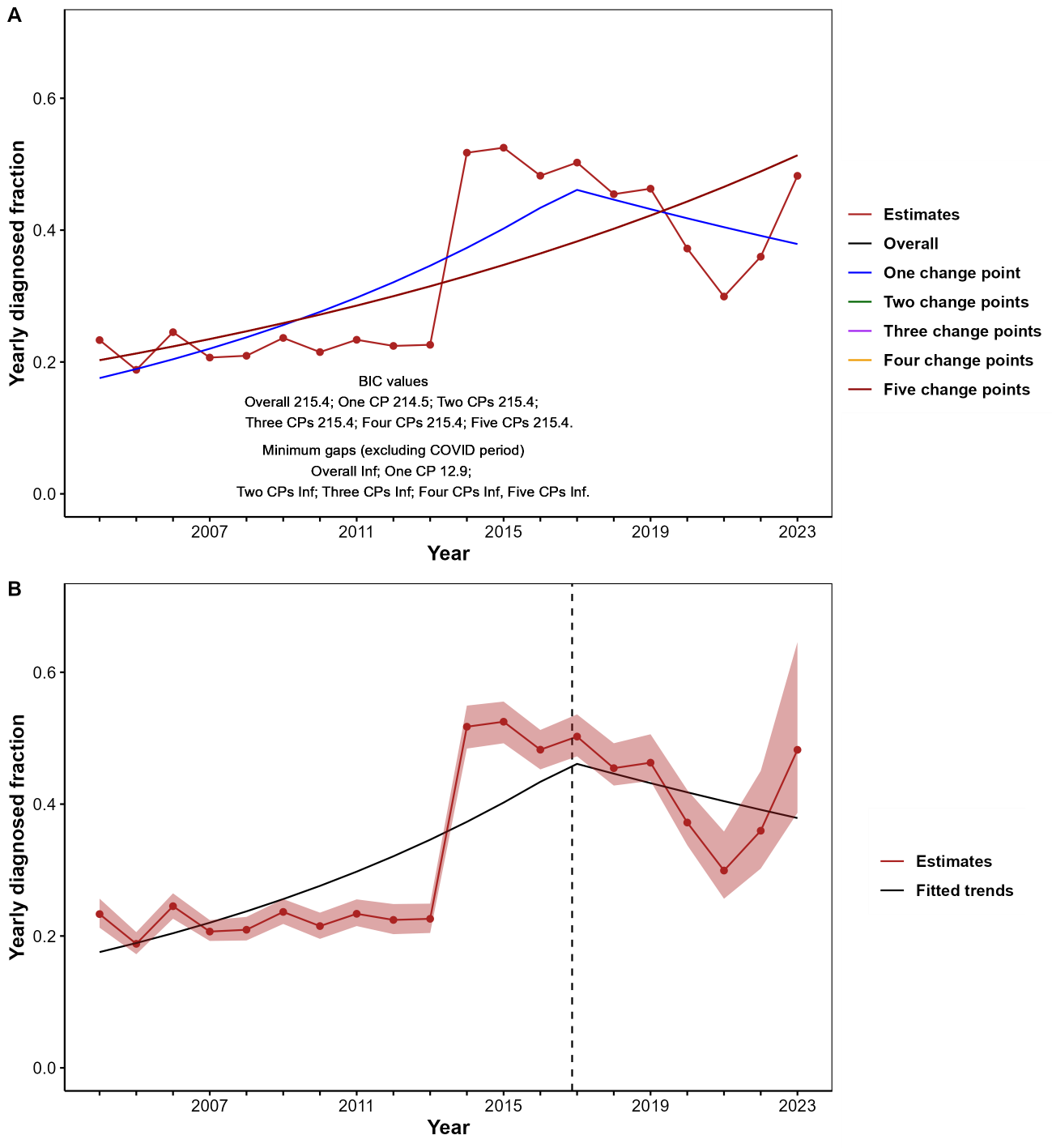


**Fitted models for the case detection rate (CDR) (A) and the best fitting two change point (CP) model (B) with the estimated change points (vertical dashed lines)**
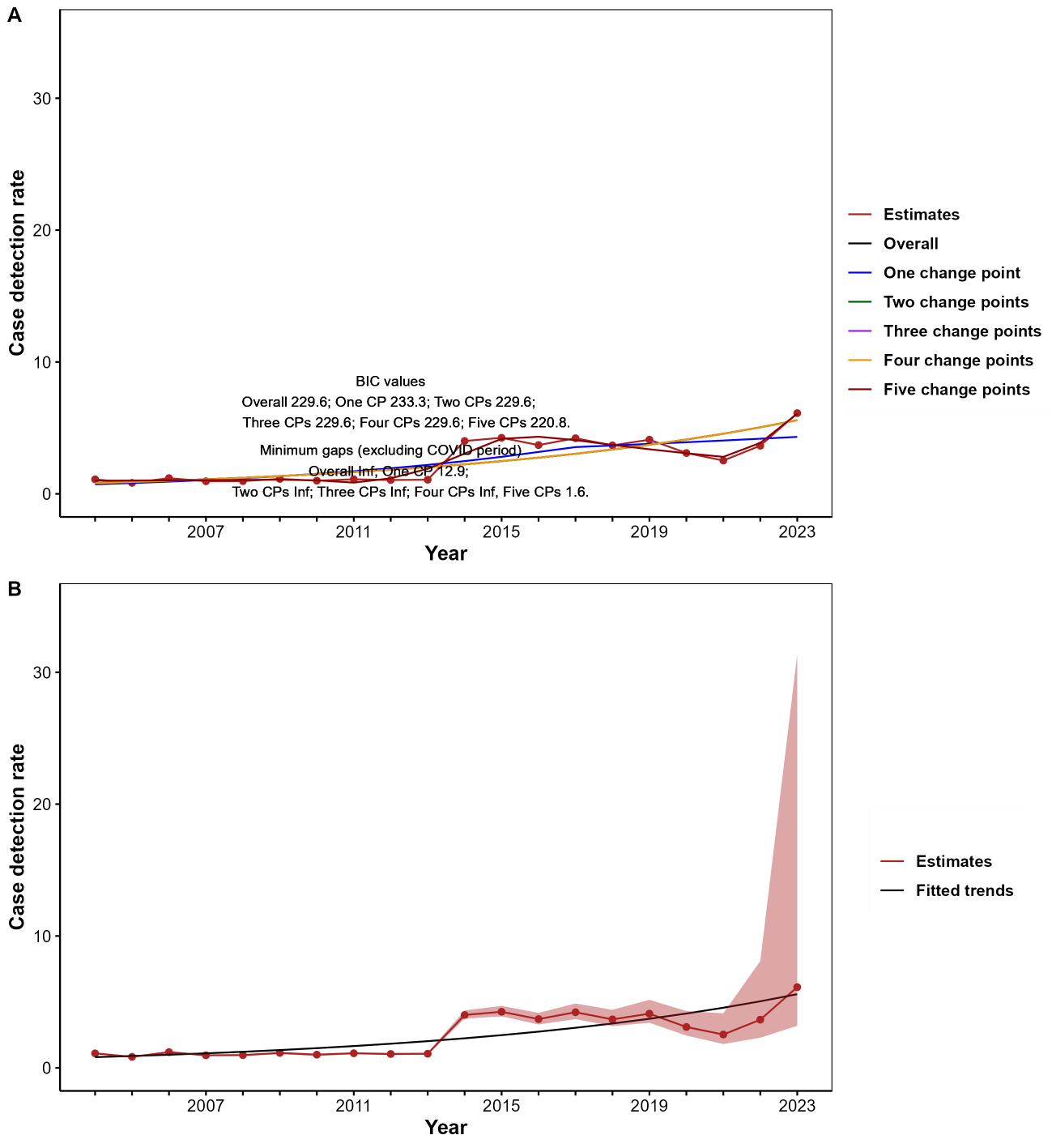


**Fitted models for the incidence prevalence ratio (IPR) (A) and the best fitting two change point (CP) model (B) with the estimated change points (vertical dashed lines)**
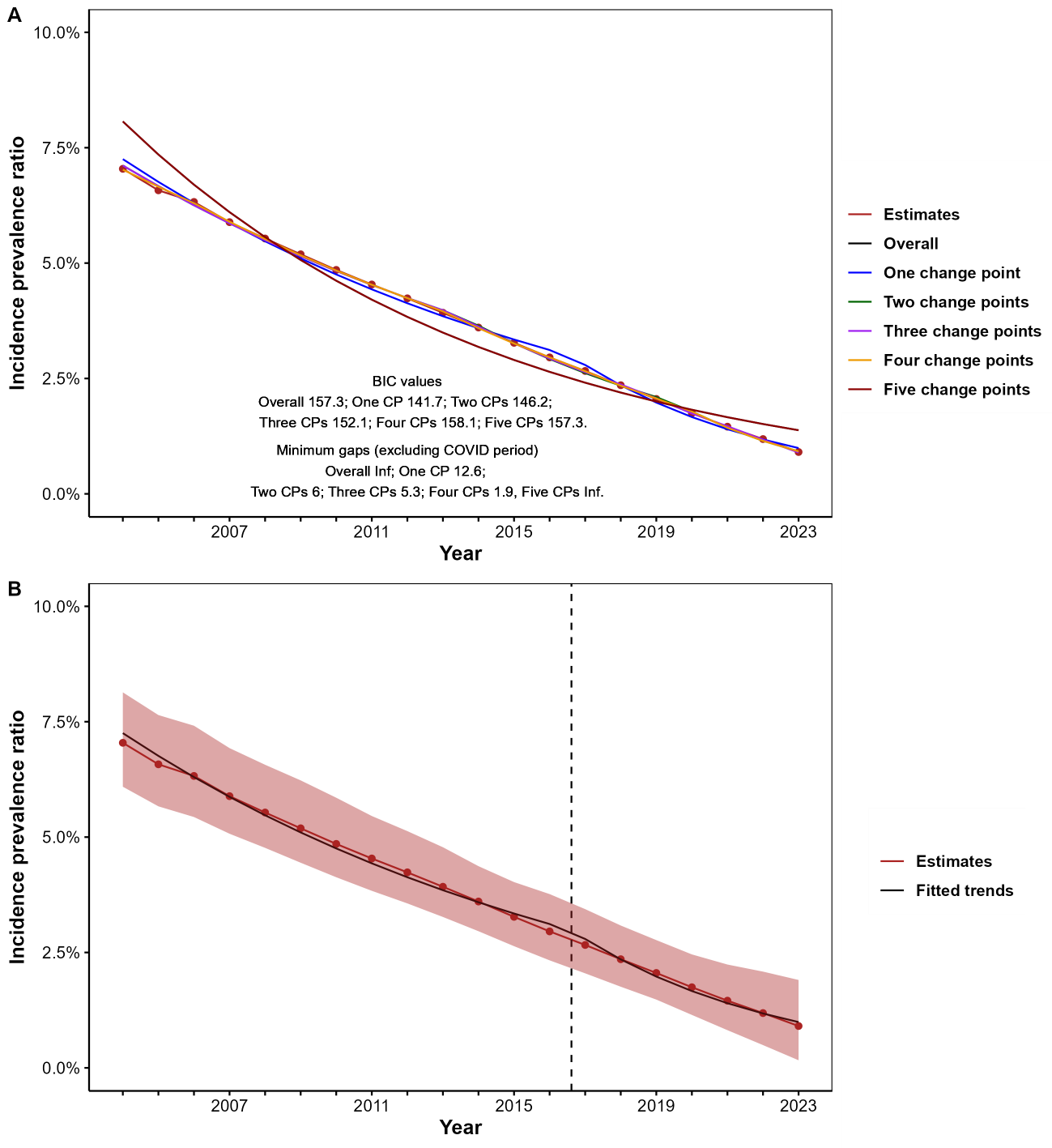


**Fitted models for the incidence mortality ratio (IMR) (A) and the best fitting one change point (CP) model (B) with the estimated change point (vertical dashed line)**
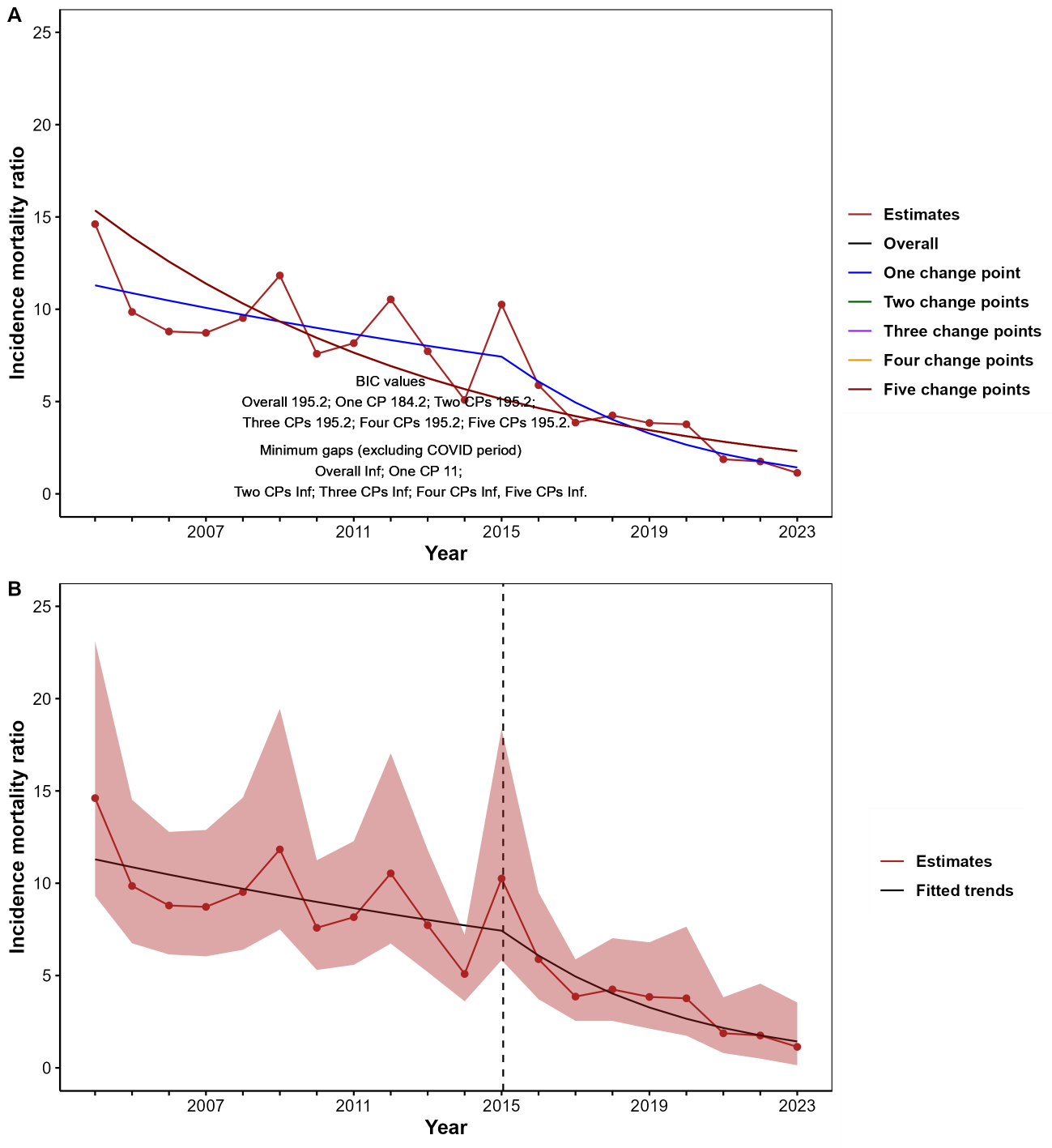


# Software used

All the cascade indicator estimates and trend analysis results were produced using the R (version: 4.3.2)^26^ and RStudio (version 2024.12.0+467) with the following packages (version in brackets):

| - AER (v 1.2.12) - aod (v 1.3.3) ^27^ - broom (v 1.0.5) - captioner (v 2.2.3.9)^28^ - car (v 3.1.2) - carData (v 3.0.5) - changepoint (v 2.2.4)^29,30^ - cowplot (v 1.1.3)^31^ - dials (v 1.2.0) - dplyr (v 1.1.4) - forcats (v 1.0.0) - forecast (v 8.21.1)^32,33^ - ggfortify (v 0.4.16)^34^ - ggplot2 (v 3.4.4) - gridExtra (v 2.3)^35^ - Hmisc (v 5.1.1) - infer (v 1.0.6) - knitr (v 1.45) - lmtest (v 0.9.40) - lubridate (v 1.9.3) - MASS (v 7.3.60) - mice (v 3.16.0)^3^ - minpack.lm (v 1.2.4) - modeldata (v 1.3.0) - nlme (v 3.1.163) - parsnip (v 1.1.1) - purrr (v 1.0.2) - RColorBrewer (v 1.1.3) - readr (v 2.1.5) - readxl (v 1.4.3) - recipes (v 1.0.9) - rsample (v 1.2.0) | - sandwich (v 3.1.0) - scales (v 1.3.0)^36^ - segmented (v 2.0.2)^24,25^ - stringr (v 1.5.1) - strucchange (v 1.5.3)^37,38^ - survival (v 3.5.7) - svglite (v 2.1.3) - tibble (v 3.2.1) - tidymodels (v 1.1.1) - tidyr (v 1.3.1) - tidyverse (v 2.0.0)^39^ - tseries (v 0.10.55)^40^ - tune (v 1.1.2) - workflows (v 1.1.3) - workflowsets (v 1.0.1) - yardstick (v 1.3.0) - zoo (v 1.8.12) |
| --- | --- |

The following scripts within the Australian diagnosis and care cascades GitHub repository (<https://github.com/The-Kirby-Institute/Cascade_calculations/>) are the primary scripts for the generation of the HIV cascade and metric estimates.^1^

| **Script within HIV folder** | **Description** |
| --- | --- |
| 0-GenerateAdjustments.Rmd | Code for cleaning and merging all the data inputs for the cascade calculations |
| 1-HivTreatment.Rmd | Specific script for producing the number of people on ART and the number with a suppressed viral load (last two steps of the cascade) |
| 1-PldhivCalculations.Rmd; code/CalculatePldhiv.R | Script and function to calculate the number of people living with diagnosed HIV and the number retained in care (second and third steps of the cascade) |
| 2-UndiagnosedCalculations.Rmd | Script to copy and read in the results from the ECDC HIV Modelling Tool and calculate the percentage undiagnosed, the number of new infections and the overall number of people living with HIV (the first step of the cascade) |
| 3-HivCascadeMerge.Rmd | This script is used to merge the estimates for each step of the cascade into one results file for the whole cascade |
| 4-HivCascadeIndicators.Rmd | Script to produce the estimates for the YDF, CDR, IPR, IMR |
| 4-HivCascadeTrends.Rmd | This script contains all the code used to explore the trends in each of the metrics. It produces the final trend analysis results and plots |

# STROBE checklist for observational studies

|  | Item No. | Recommendation | Status | Location/Notes/Relevant text from manuscript |
| --- | --- | --- | --- | --- |
| **Title and abstract** | 1 | (*a*) Indicate the study’s design with a commonly used term in the title or the abstract | Yes | Title states this is “a retrospective analysis” |
|  |  | (*b*) Provide in the abstract an informative and balanced summary of what was done and what was found | Yes | The abstract includes methods summary and key results |
| Introduction | | | |  |
| Background/rationale | 2 | Explain the scientific background and rationale for the investigation being reported | Yes | Introduction summarises HIV cascades and other metrics for understanding HIV epidemics and the characteristics of Australia’s HIV epidemic. |
| Objectives | 3 | State specific objectives, including any prespecified hypotheses | Yes | Third paragraph of Introduction states the aim of the study: “The aim of our study was to conduct an epidemiological retrospective analysis of Australia’s HIV epidemic and to assess how it has changed over time. To do this we estimated each step of the HIV cascade, the “gaps” in the cascade, annual new HIV infections, and other epidemiological metrics over the 20-year period from 2004 to 2023 using data from Australia’s multi-tiered HIV surveillance system” |
| Methods | | | |  |
| Study design | 4 | Present key elements of study design early in the paper | Yes | Third paragraph of Introduction and first section of the Methods. |
| Setting | 5 | Describe the setting, locations, and relevant dates, including periods of recruitment, exposure, follow-up, and data collection | Yes | Australia national, 2004-2023, data from multi-tiered HIV surveillance system. Third paragraph of Introduction. |
| Participants | 6 | (*a*) *Cohort study*—Give the eligibility criteria, and the sources and methods of selection of participants. Describe methods of follow-up  *Case-control study*—Give the eligibility criteria, and the sources and methods of case ascertainment and control selection. Give the rationale for the choice of cases and controls  *Cross-sectional study*—Give the eligibility criteria, and the sources and methods of selection of participants | NA |  |
|  |  | (*b*) *Cohort study*—For matched studies, give matching criteria and number of exposed and unexposed  *Case-control study*—For matched studies, give matching criteria and the number of controls per case | NA |  |
| Variables | 7 | Clearly define all outcomes, exposures, predictors, potential confounders, and effect modifiers. Give diagnostic criteria, if applicable | NA |  |
| Data sources/ measurement | 8* | For each variable of interest, give sources of data and details of methods of assessment (measurement). Describe comparability of assessment methods if there is more than one group | Yes | HIV notifications from Australia’ National HIV Registry Australian Bureau of Statistics (ABS), Pharmaceutical Benefits Scheme (PBS), Australian HIV Observational Database (AHOD). HIV Data Linkage Study 2015–2022. Described throughout the Methods and Supplementary Appendix Section 1. |
| Bias | 9 | Describe any efforts to address potential sources of bias | Yes | All assumptions described for the calculations, missing data and imputation methods noted, and limitations of representatives of data acknowledged (including used of general population movement data) |
| Study size | 10 | Explain how the study size was arrived at | NA | All HIV notifications used in Australia; no sample size calculations needed |

| Quantitative variables | 11 | Explain how quantitative variables were handled in the analyses. If applicable, describe which groupings were chosen and why | Yes | HIV notifications: year diagnosis, state or territory, region, age, gender, mode of exposure, country of birth, previous diagnosis overseas, and CD4 count at diagnosis. ABS: proportion of general population who permanently moved overseas. PBS: number receiving treated in calendar year. AHOD: proportion who died, proportion with suppressed viral load last test. HIV Data Linkage: proportion linked and linked who were on ART. |
| --- | --- | --- | --- | --- |
| Statistical methods | 12 | (*a*) Describe all statistical methods, including those used to control for confounding | Yes | Deduplication method based on date of birth. Continuous piecewise negative binomial regression models (using segmented R package). Described in the Methods section “Statistical Analysis” and Supplementary Appendix 1.1, 1.6, and 3. Other calculations performed using R scripts and the ECDC HIV Modelling tool. |
|  |  | (*b*) Describe any methods used to examine subgroups and interactions | NA | Overall population and males and females used same methods. |
|  |  | (*c*) Explain how missing data were addressed | Yes | Missing data in HIV notifications was filled using multivariate imputation with the MICE R package (version 3.16.0). Supplementary Appendix 1.1 |
|  |  | (*d*) *Cohort study*—If applicable, explain how loss to follow-up was addressed  *Case-control study*—If applicable, explain how matching of cases and controls was addressed  *Cross-sectional study*—If applicable, describe analytical methods taking account of sampling strategy | NA |  |
|  |  | (*e*) Describe any sensitivity analyses | NA |  |
| Results | | | | |
| Participants | 13* | (a) Report numbers of individuals at each stage of study—eg numbers potentially eligible, examined for eligibility, confirmed eligible, included in the study, completing follow-up, and analysed | Yes | Overall population studied will all HIV notifications including people previously diagnosed overseas. Estimates for Tans and gender diverse people not included because the low number of HIV notifications preclude production of robust estimates. Second paragraph in Methods. |
|  |  | (b) Give reasons for non-participation at each stage | NA |  |
|  |  | (c) Consider use of a flow diagram | NA |  |
| Descriptive data | 14* | (a) Give characteristics of study participants (eg demographic, clinical, social) and information on exposures and potential confounders | Yes | All people notified with HIV in Australia. The analyses were applied to the overall population and males and females separately using the same method and population specific data. |
|  |  | (b) Indicate number of participants with missing data for each variable of interest | Yes | For the HIV notifications data: year of diagnosis (complete), state or territory (complete), region of diagnosis within Australia (26.4% missing overall; 3.3% missing since 2004), age when diagnosed (0.41% missing overall), gender (0.54% missing overall, only n =6 since 2004), mode of exposure group (13.0% missing overall; 3.5% missing since 2004), and country of birth (26.2% missing overall; 1.4% missing since 2004). Supplementary Appendix 1.1 |
|  |  | (c) *Cohort study*—Summarise follow-up time (eg, average and total amount) | NA |  |
| Outcome data | 15* | *Cohort study*—Report numbers of outcome events or summary measures over time | NA |  |
|  |  | *Case-control study—*Report numbers in each exposure category, or summary measures of exposure | NA |  |
|  |  | *Cross-sectional study—*Report numbers of outcome events or summary measures | NA |  |
| Main results | 16 | (*a*) Give unadjusted estimates and, if applicable, confounder-adjusted estimates and their precision (eg, 95% confidence interval). Make clear which confounders were adjusted for and why they were included | Yes | HIV Cascade step estimates: best estimate and range (undiagnosed proportion = 95% CI from ECDC HIV Modelling Tool).  Trends: time of changepoint reported with 95% CIs and the annual rate ratio (ARR) for each segment between change points. Bayesian information criterion (BIC) used to select best fitting model. Described throughout the Methods section. |
|  |  | (*b*) Report category boundaries when continuous variables were categorized | Yes | Excluded change points within four years of each other or 2004. Allowed change points within four years of 2023 given the potential impact of the COVID-19 pandemic. Methods “Statistical analysis” section. |
|  |  | (*c*) If relevant, consider translating estimates of relative risk into absolute risk for a meaningful time period | NA |  |

| Other analyses | 17 | Report other analyses done—eg analyses of subgroups and interactions, and sensitivity analyses | Yes | New infections estimated using ECDC modelling tool. Yearly Diagnosed Fraction (YDF), Case Detection Rate (CDR), Incidence-prevalence ratio (IPR), Incidence-mortality ratio (IMR) where also calculated for the population overall and males and females. Results described in Results in “Overall trends in other epidemiological metrics” section. Supplementary Appendix 2 and 3. |
| --- | --- | --- | --- | --- |
| Discussion | | | | |
| Key results | 18 | Summarise key results with reference to study objectives | Yes | Described in the first paragraph of the Discussion. |
| Limitations | 19 | Discuss limitations of the study, taking into account sources of potential bias or imprecision. Discuss both direction and magnitude of any potential bias | Yes | Described in the second last paragraph of the Discussion. |
| Interpretation | 20 | Give a cautious overall interpretation of results considering objectives, limitations, multiplicity of analyses, results from similar studies, and other relevant evidence | Yes | Results contextualised against comparable high-income countries, potential impact of interventions such as treatment as prevention and PrEP, the impact of COVID-19, and the changing nature of Australia’s HIV epidemic. Results for males and females compared. |
| Generalisability | 21 | Discuss the generalisability (external validity) of the study results | Yes | Implications of results on ending HIV as a public health threat and importance of sustaining programs to ensure appropriate care and quality of life for people living with HIV and to avoid a reversal in progress. Last paragraph of Discussion. |
| Other information | |  | | |
| Funding | 22 | Give the source of funding and the role of the funders for the present study and, if applicable, for the original study on which the present article is based | Yes | Specific funding noted below the abstract and in the Acknowledgements. Role of funding source section at end of Methods. |

**Status key:** Yes = fully reported; N/A = not applicable to this study design.

# References

1 Gray RT, Neil Bretana P. The-Kirby-Institute/Cascade_calculations: Version for Australian HIV Cascade Manusript. 2026; published online 30 March 2026. DOI:10.5281/zenodo.19324612.

2 King, J, McManus, H, Kwon, A, Gray, R, McGregor, S. HIV, viral hepatitis and sexually transmissible infections in Australia: Annual surveillance report 2024. The Kirby Institute, UNSW Sydney, Sydney, Australia, 2024.

3 Buuren S van, Groothuis-Oudshoorn K. mice: Multivariate Imputation by Chained Equations in R. J Stat Softw 2011; 45: 1–67.

4 Law MG, McDonald AM, Kaldor JM. Estimation of cumulative HIV incidence in Australia, based on national case reporting. Aust N Z J Public Health 1996; 20: 215–7.

5 Nakhaee F, Black D, Wand H, McDonald A, Law M. Changes in mortality following HIV and AIDS and estimation of the number of people living with diagnosed HIV/AIDS in Australia, 1981–2003. Sex Health 2009; 6: 129–34.

6 McManus H, O’Connor CC, Boyd M, et al. Long-term survival in HIV positive patients with up to 15 Years of antiretroviral therapy. PLOS One 2012; 7: e48839.

7 NSW Health. NSW HIV Strategy 2021-2025: Annual data report 2024. Sydney, Australia: NSW Health, 2024. https://www.health.nsw.gov.au/endinghiv/pages/tools-and-data.aspx (accessed Sept 29, 2025).

8 Australian Bureau of Statistics (ABS). 3401.0 - Overseas Arrivals and Departures, Australia, Jun 2017 (Archived issue); published online Aug 15 2017. https://www.abs.gov.au/AUSSTATS/abs@.nsf/Lookup/3401.0Main+Features1Jun%202017?OpenDocument= (accessed Sept 29, 2025).

9 Overseas Arrivals and Departures, Australia | Australian Bureau of Statistics. https://www.abs.gov.au/statistics/industry/tourism-and-transport/overseas-arrivals-and-departures-australia (accessed Sept 29, 2025).

10 Overseas Arrivals and Departures, Australia, Latest Release | Australian Bureau of Statistics. https://www.abs.gov.au/statistics/industry/tourism-and-transport/overseas-arrivals-and-departures-australia/latest-release (accessed Sept 29, 2025).

11 National, state and territory population | Australian Bureau of Statistics. https://www.abs.gov.au/statistics/people/population/national-state-and-territory-population (accessed Sept 29, 2025).

12 van Sighem A, Nakagawa F, De Angelis D, et al. Estimating HIV incidence, time to diagnosis, and the undiagnosed HIV epidemic using routine surveillance data: Epidemiology 2015; 26: 653–60.

13 ECDC HIV modelling tool [software application]. Version 1.3.0. European Centre for Disease Prevention and Control, 14 Sep 2015 https://www.ecdc.europa.eu/en/publications-data/hiv-modelling-tool (accessed Feb 2, 2025).

14 King J, Petoumenos K, Dobbins TA, et al. A population level application of a novel method for estimating the timing of HIV acquisition among migrants to Australia. JIAS 2022; 26: e26127.

15 Holt M, Lea T, Asselin J, et al. The prevalence and correlates of undiagnosed HIV among Australian gay and bisexual men: results of a national, community-based, bio-behavioural survey. J Int AIDS Soc 2015; 18: 20526.

16 McMahon J, Moore R, Eu B, et al. Clinic network collaboration and patient tracing to maximize retention in HIV care. PloS One 2015; 10: e0127726.

17 Bhatt S, Bryant M, Lau H, et al. Successful expanded clinic network collaboration and patient tracing for retention in HIV care. AIDS Res Ther 2022; 19: 61.

18 UNAIDS. Global AIDS Monitoring 2025: Indicators and questions for monitoring progress on the 2021 Political Declaration on HIV and AIDS. Geneva UNAIDS 2024.

19 The Pharmaceutical Benefits Scheme: Section 100–Highly Specialised Drugs Program. Australian Government, Department of Health, Disability and Aging https://www.pbs.gov.au/info/browse/section-100/s100-highly-specialised-drugs (accessed Sept 29, 2025).

20 Petoumenos K, Watson J, Whittaker B, et al. Subsidized optimal ART for HIV-positive temporary residents of Australia improves virological outcomes: results from the Australian HIV Observational Database Temporary Residents Access Study. J Int AIDS Soc 2015; 18.

21 NAPWHA. Medicare ineligible PLHIV in Australia: An analysis of new data with recommendations for systemic improvements. NAPWHA, 2019 https://napwha.org.au/wp-content/uploads/2019/07/2019_NAPWHA_MedicareIneligiblesPLHIVinAustralia_Analysis.pdf (accessed Sept 29, 2025).

22 The Kirby Institute. The Australian HIV Observational Database Temporary Residents Access Study (ATRAS) One year follow-up. Sydney, NSW: The Kirby Institute, UNSW Australia; 2013.

23 Mellish L, Karanges EA, Litchfield MJ, et al. The Australian Pharmaceutical Benefits Scheme data collection: a practical guide for researchers. BMC Res Notes 2015; 8: 634..

24 Muggeo VMR. segmented: An R Package to Fit Regression Models with Broken-Line Relationships. R News 2008; 8: 20–5.

25 Muggeo VMR. segmented: An R Package to Fit Regression Models with Broken-Line Relationships. 2017; published online Sept 18. https://cran.r-project.org/web/packages/segmented/index.html (accessed Feb 16, 2024).

26 R Core Team. R: A Language and Environment for Statistical Computing. 2017. https://www.R-project.org/..

27 Lesnoff, M., Lancelot, R. aod: Analysis of Overdispersed Data. 2012. http://cran.r-project.org/package=aod (accessed Feb 16, 2024).

28 Alathea L. captioner: Numbers Figures and Creates Simple Captions. 2015. https://github.com/adletaw/captioner (archived repository; accessed Sept 29, 2025).

29 Killick R, Eckley IA. changepoint: An R Package for Changepoint Analysis. J Stat Softw 2014; 58: 1–19.

30 Killick R, Eckley IA. changepoint: An R package for changepoint analysis. 2022. https://CRAN.R-project.org/package=changepoint (accessed Sept 29, 2025).

31 Wilke CO. cowplot: Streamlined Plot Theme and Plot Annotations for ‘ggplot2’. 2016. https://CRAN.R-project.org/package=cowplot (accessed Sept 29, 2025).

32 Hyndman RJ. forecast: Forecasting functions for time series and linear models. 2017. http://github.com/robjhyndman/forecast.

33 Hyndman RJ, Khandakar Y. Automatic time series forecasting: the forecast package for R. J Stat Softw 2008; 26: 1–22.

34 Horikoshi M, Tang Y. ggfortify: Data Visualization Tools for Statistical Analysis Results. 2018. https://CRAN.R-project.org/package=ggfortify.

35 Auguie B. gridExtra: Miscellaneous Functions for ‘Grid’ Graphics. 2022. https://CRAN.R-project.org/package=gridExtra (accessed Sept 29, 2025).

36 Wickham H. scales: Scale Functions for Visualization. 2023; published online Nov 28. https://CRAN.R-project.org/package=scales (accessed Feb 16, 2024).

37 Zeileis A, Leisch F, Hornik K, Kleiber C. strucchange: An R Package for Testing for Structural Change in Linear Regression Models. J Stat Softw 2002; 7: 1–38.

38 Zeileis A, Leisch F, Hornik K, Kleiber C, Hansen B, Merkle EC. strucchange: An R Package for Testing for Structural Change in Linear Regression Models. 2022; published online June 15. https://cran.r-project.org/web/packages/strucchange/index.html (accessed Feb 16, 2024).

39 Wickham H. tidyverse: Easily Install and Load the ‘Tidyverse’. 2023; published online Feb 22. https://CRAN.R-project.org/package=tidyverse (accessed Feb 16, 2024).

40 Trapletti A, Hornik K. tseries: Time Series Analysis and Computational Finance. 2023; published online Dec 6. https://CRAN.R-project.org/package=tseries (accessed Feb 16, 2024).
